# Dynamic Screening and the Chemical Inductor of Perovskite Solar Cells: From *J*–*V* Transients to Impedance Spectroscopy

**DOI:** 10.1021/acs.jpclett.5c01916

**Published:** 2025-10-15

**Authors:** Enrique H. Balaguera, Elnaz Ghahremani Rad, Alexander R. Uhl, Antonio Guerrero, Juan Bisquert

**Affiliations:** † Escuela Superior de Ciencias Experimentales y Tecnología (ESCET), 16776 Universidad Rey Juan Carlos, 28933 Móstoles, Madrid, Spain; ‡ Laboratory for Solar Energy & Fuels (LSEF), School of Engineering, University of British Columbia, Kelowna V1V 1V7, Canada; § Institute of Advanced Materials (INAM), Universitat Jaume I, 12006 Castelló, Spain; ∥ Instituto de Tecnología Química (Universitat Politècnica de València-Agencia Estatal Consejo Superior de Investigaciones Científicas), Av. dels Tarongers, 46022 València, Spain

## Abstract

The operation of metal halide perovskite solar cells is governed by the interplay between electronic transport and recombination processes strongly modulated by ionic dynamics. We present a unified framework that disentangles the photocurrent into distinct ionic–electronic conductivity pathways, incorporating recombination, polarization, and charge-collection effects. Using simplified dynamic models, we describe how electric-field screening and slow ionic relaxation shape the hysteresis of the photocurrent. From the nonlinear differential equations, we derive the corresponding impedance response and show that the characteristic low-frequency “double inductor” feature arises from two separate mechanisms: ion-controlled recombination and ionic screening of the internal electric field. This approach offers a general route to connect transient photocurrent phenomena with impedance signatures, providing new tools to identify and classify degradation pathways in perovskite solar cells.

## Graphical abstract

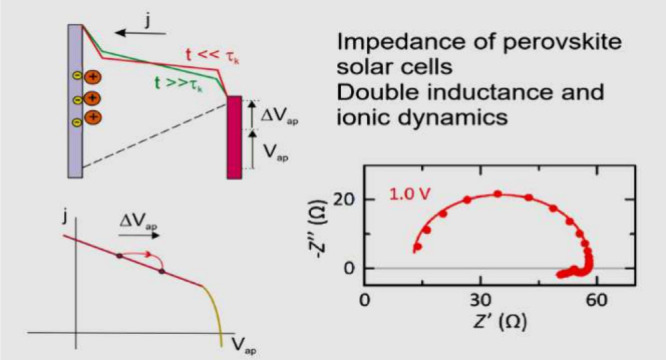

Although nowadays perovskite solar cells exhibit astonishing values of power conversion efficiency, this photovoltaic technology still faces long-term operational stability issues.^1–3^ Before mass implementation and commercialization of perovskite photovoltaics becomes a reality, it is therefore essential to develop a comprehensive understanding of the physical processes involved in device aging and their impact on performance parameters. Among the multitude of factors contributing to the efficiency losses, the presence of mobile ions appears to be the general reason that negatively affect the stability of perovskites.

Ionic redistribution can produce large carrier accumulation, leading to, for instance, electric field screening, interfacial recombination, surface polarization, and charge collection losses,^4–6^ ultimately reducing photocurrent and photovoltage. Mobile ions also facilitate reactions with water and oxygen molecules,^7^ light-induced phase segregation^8,9^ and diffusion of metal contacts atoms into the perovskite layer,^10,11^ which are the main causes of decreased performance and stability. The effects of ions producing slow recombination and polarization components have been amply described in the recent years, especially by application of impedance spectroscopy (IS) techniques.^12–18^ Here, we extend the previous methodologies by providing a description of charge collection issues that appear when the electrical field is substantially influenced by mobile ions.

There has been an intense debate in the literature regarding ion-induced field screening of the electric field as a key factor affecting the operational stability of perovskite devices.^19–26^ This phenomenon leads to a significant decrease in short-circuit current density (*j*_sc_) and fill factor (FF), making it difficult to evaluate the device efficiency due to complex hysteresis effects. Ionic charge accumulation can be experimentally determined based on capacitance measurements.^27–29^ By detailed interfacial models it is possible to establish the influence of ions on surface recombination, which can be beneficial for performance in some circumstances.^30,31^ Beyond open-circuit voltage (*V*_oc_) variations adequately described by advanced theory of ion-driven recombination and polarization,^15^ it is therefore necessary to formulate a complete model including the idea that mobile ions also screen the internal electric field,^20–26^ causing anomalous current–voltage hysteresis patterns around *j*_sc_ and impedance spectra with multiple inductor features.

The aim of this paper is to establish a compact set of equations that can address several experimental methods, such as IS response and current–voltage scans,^13–17^ combining both dominant surface recombination, and charge collection limitation effects. We report a distinction between two types of inductive processes: the first is attributed to surface recombination governed by mobile ions, while the second is associated with limitations in charge collection due to field screening effects. Depending on the leading degradation mechanism affecting the device, one can differentiate between these phenomena—or, in certain cases, observe both— along with surface polarization of a capacitive-inductive nature, by employing a combination of impedance and current–voltage analysis methods. We show experimental examples of the connection of impedance patterns to hysteresis effects, including a notorious double inductor feature, in addition to the standard double capacitive arcs. We summarize the impact of the theoretical analysis with numerical simulations of current–voltage curves, followed by the presentation of an equivalent circuit estimated through small-perturbation approximations for use in the characterization of impedance responses, and also the associated hysteresis effects in current–voltage curves. Our results support the mathematical model by the combination of different device operating regimes, reproducing the shape of current–voltage characteristics with complex hysteresis and impedance spectra with multiple facets observed commonly in experimental measurements.

Hysteresis and light-induced negative capacitance effects have been amply observed and discussed in perovskite solar cells.^32,33^ A majority of approaches coincide in attributing the primary cause to a sluggish ionic dynamic that in the end impacts the externally measured currents. The crucial method of analysis of perovskite solar cells by IS consists of tracking the evolution over a succession of steady states, obtaining the model parameters, and observing how they change under different external conditions. Most models have in common that internal state variables are defined, which describe the ionic delay and its impact on recombination and charge accumulation phenomena.^12–16^ These methods have been applied by different groups.^34–37^

To summarize the connection of impedance and hysteresis during current-voltage cycling, we discuss some elementary models that contain essential physical ingredients of the perovskite solar cell dynamics. We assume the following equation for the current in the solar cell

1 $$j=J_{rec}\left(V\right)+\frac{dQ_{s}\left(x\right)}{dt}$$

Here *J*_*rec*_(*V*) is recombination current that is a direct function of the external voltage *V* and *Q*_*s*_(*x*) is a charge function that depends on an internal variable *x*, such as a surface voltage *v*_*s*_. This internal variable adapts to the changes of applied voltage *V* by the equation

2 $$\tau_{s}\frac{dx}{dt}=F\left(V\right)-x$$

where *F*(*V*) is a nonlinear function and *τ*_*s*_ is a relaxation time. In the Appendix, we show that the impedance due to the charge derivative in eq 1 is

3 $$Z=\frac{1}{g_{C}}+\frac{1}{C_{s}i\omega }$$

where *C*_*s*_ = *f* d*Q*_*s*_/d*x* is a capacitance, *f* = d*F*/d*V*, and *g*_*C*_ = *C*_*s*_/*τ*_*s*_ is a conductance. Equation 3 describes a series *RC* circuit.

If, however, the current has the expression

4 $$j=j_{a}\left(x\right)$$

for a nonlinear function *j*_*a*_ of the variable *x*, that is also controlled by eq 2, then the impedance

5 $$Z=\frac{1}{g_{L}}+L_{b}i\omega$$

is a series connection of a resistor and an inductor, where *g*_*L*_ = *mf* is a conductance, *m*(*x*) = d*j*_*a*_/d*x*, and *L*_*b*_ = *τ*_*s*_/*mf* is an inductance.

Note that the variable *x* in eq 2 can have many different interpretations according to the specific system. The eq 2 is first found in the Hodgkin-Huxley (HH) model for neuronal behavior.^38^ This model enables a description of the transients of the ionic current that produce the action potential^39,40^ by using several *x*-type variables that represent the gating of ionic channels.^41^ Equations 2 and 4 also form the fundamental framework of the theory of memristors^42,43^ where *x* indicates the state of a conducting filament. In perovskite solar cells, the variable *x* usually represents an internal voltage *v* or current *j*.^13,16^

The above demonstration, presented in further details in the Appendix, shows that the type of electrical response of the slow variable *x* depends on the form of the current. If the current is a time derivative of *x*, the response is capacitive. But if the current is a direct function of *x* in eq 4 then the response is inductive. Equations 2 and 4 can be obtained by a large variety of specific mechanisms, and the set of two equations was termed a chemical inductor,^44^ which is just a generic denomination. In the framework of perovskite solar cells, a surface polarization model can be formulated in which both the capacitive current and the recombination current depend on the surface ionic charge, *Q*_*s*_(*v*_*s*_).^17^ Then, both time constants for the inductor and the capacitor are the same, which is observed experimentally.^45^

The same approach we have presented provides an explanation of basic hysteresis features. Hysteresis can be analyzed by observing the current–voltage in stable voltage cycling, or in response to a step voltage Δ*V*_*ap*_.^46^ Any delayed response in the device, such as described by eq 2, produces a temporary departure from the equilibrium line which gives a hysteresis effect. Typically, a capacitive process in the system produces a temporary charging current. As we show in the Appendix, under a voltage step, the current response determined by eqs 1 and 2 is^13^

6 $$\Delta j\left(t\right)=g_{C}\Delta V_{ap}e^{-t/\tau_{s}}$$

The current decreases from an initial spike until the capacitance is fully charged when Δ*j* = 0. In the *jV* (current-voltage) diagram for solar cell under illumination the total current lays below the equilibrium line *J*_*rec*_(*V*).^46^ This is normal or capacitive hysteresis.^47,48^ Conversely, for the inductive system ([Disp-formula eq2], 4) we obtain

7 $$\Delta j\left(t\right)=g_{L}\Delta V_{ap}\left(1-e^{-t/\tau_{s}}\right)$$

Now the current increases at long time, until the final value *g*_*L*_Δ*V*. In a *jV* diagram the forward current occurs above the equilibrium current. In circuit terms, it resembles an inductor, which produces a voltage opposing the change in current. This is inverted or inductive hysteresis.^46^ A capacitor resists changes in voltage while an inductor resists changes in current. Both capacitor and inductor store energy similar to a water tank or flywheel, respectively. However, here the origin of the inductor is not magnetic but chemical,^44^ caused by slow ionic reorganization coupled to electronic recombination.

Our analysis shows that mechanistic explanations for inverted hysteresis that occurs under sustained voltage cycling, conceal a correspondent inductor mechanism in IS. We remark that a popular denominator of the inductive process is a “negative capacitance”. However, capacitances and inductors are very different. The impedance of a capacitance has the expression *Z* = 1/(i*ωC*) that becomes infinite at low frequencies. An inductor impedance *Z* = i*ωL* becomes short-circuit at low frequency. The feature observed in perovskite solar cells is modeled with a positive inductor, not a physical negative capacitor. Negative capacitance could be desired for certain applications, as it could lower the power consumption in field-effect transistors, by reducing the subthreshold swing factor below the thermodynamic limit of 60 mV per decade, with enormous technological impacts.^49^ Notably, we know of no case of a stable negative capacitance reported in the literature. In contrast, the chemical inductor is a common object observed across many kinds of fields.

The transients of the two kinds ([Disp-formula eq6], 7) are easily observed in perovskite solar cells,^16,50^ and the correlation of currents with IS is well established.^51,52^ Note that the time constants of the impedance branches in eq 3 containing a capacitor is *g*_*C*_^–1^ *C*_*s*_ = *τ*_*s*_ and in eq 5 containing the inductor is g_*L*_ *L*_*a*_ = *τ*_*s*_. Both correspond to the time constant *τ*_*s*_ of the delayed eq 2.

These simple models provide a basic outline that is useful for a primary classification of dynamics responses. One is aware, of course, that in the real experiments many internal variables may be coupled and more complex situations emerge.^53^ This is commented in the final part of the paper.

The most common effect observed in current–voltage curves of perovskite solar cells involves the occurrence of either inverted hysteresis at high voltages (around open-circuit voltage), or capacitive hysteresis, or both, corresponding to inductive and capacitive features identified through impedance analysis, respectively.^45^ These hysteresis effects can be attributed to the contacts, as small tuning of the transport layers can change the type of observed hysteresis.^54^ Furthermore, in these cases the separation of the current between forward and backward scans occurs only close to *V*_*oc*_, while in the region of low voltages the current remains horizontal, meaning that the charge collection region is not affected. Hence, models can be developed based on surface effects on the electronic carriers, for instance when ionic charge creates surface accumulation and influences the electronic current based on the influence of surface recombination. This effect is modeled in surface polarization models and related approaches,^17,34,55,56^ using slow recombination mechanisms for surface phenomena characterized by ideality exponents.^57^ An extensive survey of the literature by Nemnes et al.^34^ establishes a clear distinction of capacitive and inductive recombination processes, in addition to the reference recombination current.

It is also frequently reported in the literature that hysteresis can arise at relatively low voltages, below the MPP (maximum power point) region of the current–voltage curve,^4,25^ and when the current remains close to the short-circuit value.^24,32,33,58,59^ This behavior leads to an obvious slope of the photocurrent close to 0 V and separation of the current in forward and backward,^24^ as shown in the experimental results of Figure 1a for a solar cell under light, and in Figure SI1 for a solar cell in the dark, which is accompanied in both cases by the observation of a double inductor feature at low frequencies, Figure 1(d,e) and Figure SI2.

**Figure 1:**
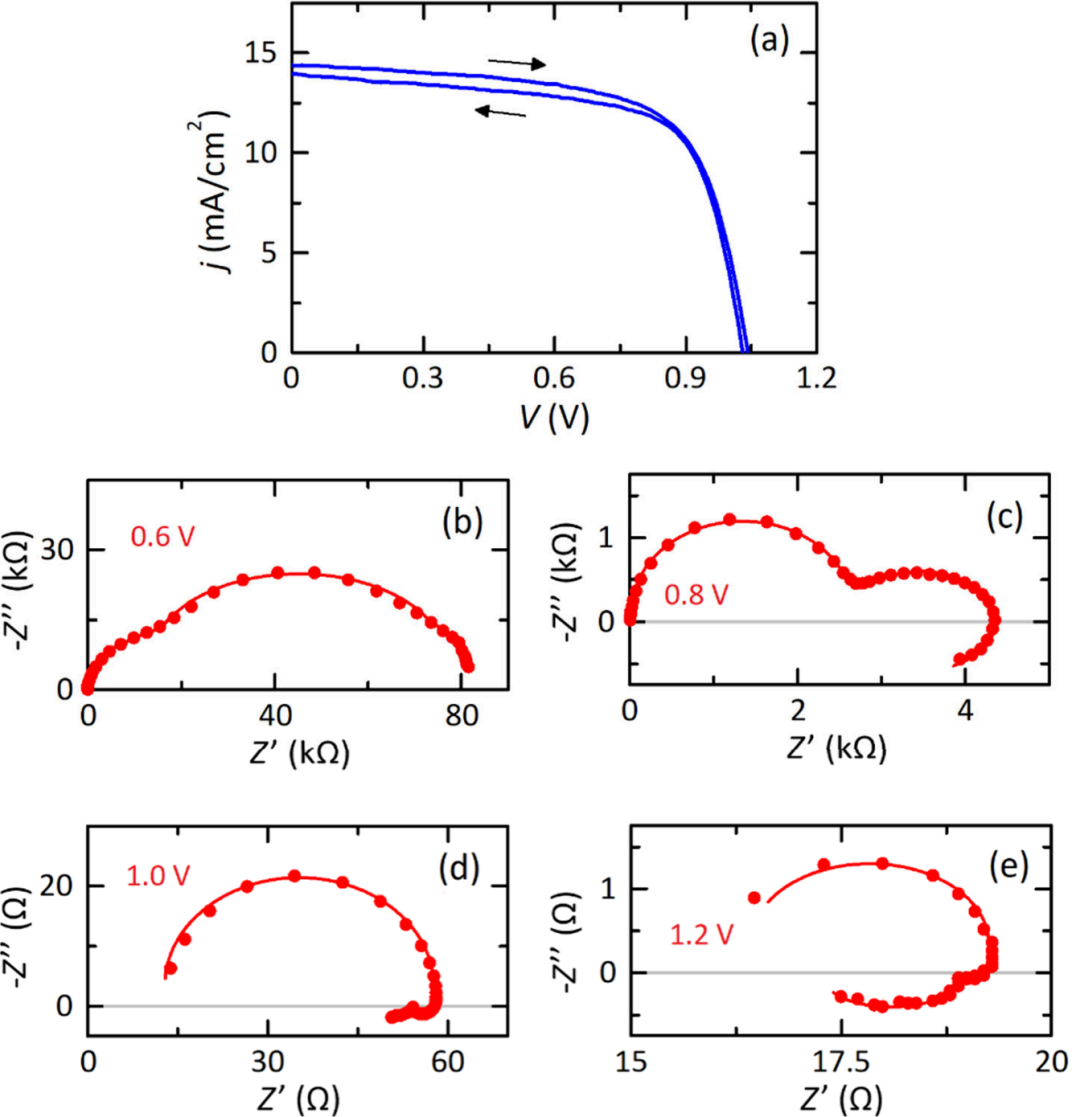
Experimental responses of an inverted perovskite solar cell with a device layer architecture consisting of FTO/NiO/MeO-2PACz/perovskite (Cs_0.05_FA_0.8_MA_0.15_PbI_2.75_Br_0.25_)/PCBM/BCP/Au/Ag. (a) Current–voltage curve obtained under a scan rate of 100 mV/s. The device was measured from −0.2 to 1.2 V in forward and reverse directions under AM1.5 G spectrum in ambient conditions (22 °C and 30% RH). Impedance spectra at (b) 0.6 V, (c) 0.8 V, (d) 1.0 V, and (e) 1.2 V under a frequency range from 1 MHz to 100 mHz.

This type of hysteresis of the photocurrent is often interpreted according to slow ionic interfacial polarization that affects the bulk electrical field and modifies the photocurrent altering the charge collection. This mechanism has been described many times.^20–26^ It is well-known that the drift field can be manipulated with slow polarization of ions. One example is the famous “preconditioning” treatment by applying a specific voltage or illumination protocol to the device before measurement, to influence ion distribution.^25^ This can lead to improved performance by enhancing built-in electric fields.^60^ In general, the light soaking can modify the ion distribution and change significantly the internal field.^61,62^ Here, we provide a summary explanation based on the elementary mechanism of Figure 2, explained in more detail in Section 4 of the Supporting Information.

**Figure 2:**
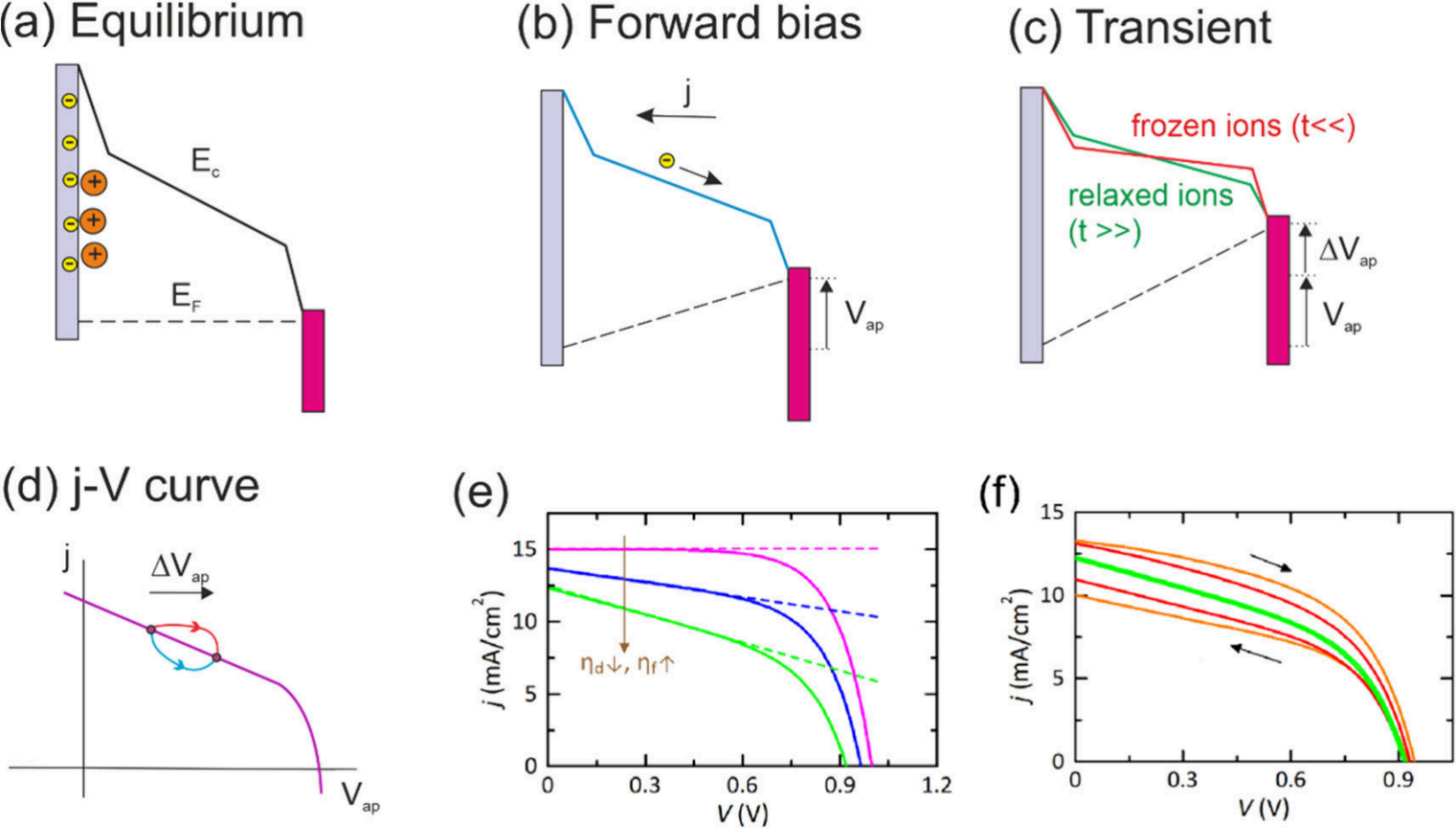
(a,b) Basic model of a solar cell where carrier transport is driven by drift in the electrical field, with different biasing conditions. The electron selective contact is the pink layer at the right, and the hole selective contact is the gray layer at the left. The valence band is parallel to the conduction band level, and it is not shown. The dipole layer in the hole selective contact is formed by electrons (yellow) and cations (orange). (c) Effect of a change of voltage. Initially (red) the dipole layers remain in the previous configuration, since the ion rearrangement is slow. When the ions relax at *t* ≫ *τ*_*k*_, the dipole layers are reduced (green), and the current increases. (d). The model generates an undershoot (blue) or an overshoot (red) of photocurrent, according to the dominant effect of charge collection or recombination, respectively. (f, g) Simulation of model curves. (e) Steady state current–voltage characteristics. The vertical arrow indicates the changes of collection efficiencies: *η*_*d*_ = 1, 0.85, 0.7; *η*_*f*_ = 0, 0.1, 0.2, and the dashed straight lines show *j*_ph_(*V*,Φ). (f) Hysteresis in the green curve of (e) by increasing the sweep velocity, *f* = 1 Hz (orange),0.5 Hz (red). Parameters *V*_m_ = −0.2 *V*, *V*_p_ = 1.2 V.

In the model, mobile ions in mixed ionic–electronic solar cells accumulate at the selective contacts and form Helmholtz double layers (HL), which act as nanocapacitors storing part of the built-in potential. The voltage is shared between semiconductor and Helmholtz layers according to their capacitances. This electrostatic screening reduces the fraction of the voltage that drops across the semiconductor bulk, weakening the internal field, which affects charge collection and recombination, provided that the diffusion length *L*_*D*_ is short. The surface ion distribution requires some time *τ*_*k*_ for the excess ions to adapt to the new voltage,^62^ and we make a distinction of relaxed and “frozen” ionic states.^19,52^ If ions are mobile, the Helmholtz voltage decreases proportionally with applied forward bias (relaxed case); if ions are immobile, the Helmholtz voltage remains fixed and the semiconductor takes the full change (frozen case). This is shown in Figure SI4.

After a sudden voltage step Δ*V*_*ap*_, the difference between these two regimes becomes explicit, as shown in Figure 2c. Immediately, the ionic configuration is frozen (red line), so the semiconductor experiences the entire perturbation and the current drops below its steady-state value. With time, ions redistribute, the HLs readjust their voltage, reaching the new equilibrium green line, and the current recovers.

To illustrate the results of this model in more detail, it is solved by drift-diffusion simulation in Section 4 of the Supporting Information. Two situations are explored.

(a) *Band-to-band recombination*: Here the illuminated current is collection-limited: When the HLs are frozen after a forward step, part of the contact potential remains trapped in the interfacial double layers, so less of the bias drops across the semiconductor than in the relaxed partition. The device sees a smaller collection field. With *L*_*D*_ < *L*, drift aids extraction; reducing the field decreases charge collection, thus *j* falls below its steady value giving a current undershoot. This is observed in Figure SI5.
(b) *Shockley-Read-Hall (RH) recombination*: Now the current is recombination-limited in the same bias window. Freezing the HL rearranges the band bending compared with the relaxed partition causing narrower high-field zones. In the regions that dominate recombination, minority-carrier densities are lower, so the recombination loss is smaller. Even though the collection field is slightly reduced by the frozen HL, the drop in SRH recombination dominates, making the total current larger than the relaxed steady state, Figure SI6. This is a current overshoot, as shown in Figure SI7.

Thus, the sign of the effect depends on what limits performance, as shown in the scheme of Figure 2d. In collection-limited (band-to-band) operation, frozen HLs reduce the field and produce an undershoot, whereas in recombination-limited (SRH) operation, frozen HLs reduce the recombination rate and produce an overshoot. These two examples based on a simplified model of the interfaces do not aim at a definite mechanistic characterization, but rather show that two types of hysteresis in the photocurrent are possible under voltage cycling, with the current in the forward scan being higher (the overshoot case), or the converse (the undershoot case).^19,63^

To characterize the effect of a constant electrical field along the bulk of the absorber layer, as in Figure 2, we suggest that the transport current *j*_ph_ can be described, starting from the fundamental basis, as

8 $$j_{\mathrm{ph}}\left(V,\Phi \right)=J_{\mathrm{ph}0}\left(\Phi \right)\left[\eta_{d}+\eta_{f}\left(\frac{V_{0}-V}{V_{0}}\right)\right]$$

Here, *V*_0_ is an effective built-in voltage under stationary operation. It corresponds to a specific steady state of bias voltage, illumination, etc. This *V*_0_ may further depend nonlinearly on ionic components, as described by Nemnes et al.^64^ in a model imported from ferroelectric systems that show large hysteresis effects.^65^ *J*_ph0_(Φ) is the charge generated under the incoming photon flux Φ, η_d_ is the efficiency of charge collection by diffusion, η_f_ is the charge collection efficiency by the drift field. Then, the photocurrent at short circuit is *j*_ph_(0,Φ) = *J*_ph0_(Φ)­(η_d_ + η_f_) where 0 ≤ (η_d_ + η_f_) ≤ 1. In the dark, *J*_ph0_(Φ = 0) is a constant that depends on the mobility and the dark carrier density.

Equation 8 describes the usual stationary situation. Of interest here is the transient behavior, that is closely related to the impedance methods. For a simple approach to these situations, we aim to describe the overshoot of photocurrent case, that matches the observed hysteresis trend in Figure 1a.^19^ We replace eq 8 with the new form

9 $$j_{\mathrm{ph}}\left(v_{b},\Phi \right)=j_{\mathrm{ph}0}\left(\Phi \right)\left[\eta_{d}+\eta_{f}\left(1-\frac{v_{b}}{V_{0}}\right)\right]$$

The internal voltage *v*_b_ is the instantaneous bulk voltage that will equilibrate as *v*_b_ → *V* in the long time, according to the eq 2

10 $$\tau_{b}\frac{dv_{b}}{dt}=V-v_{b}$$

Taking into account the dipole layers, one can introduce a more complete field distribution *F*(*V*),^37,64^ as described in Section S4 of the Supporting Information, but here we use a linear approximation. As shown in the Appendix, *v*_b_ generates another chemical inductor structure^66^ by eqs 9 and 10, that explains modification of charge collection in current–voltage responses.

The current–voltage curves and hysteresis characteristics of the model are shown in Figure 2(e,f). In Figure 2e, the standard curve for high diffusion length (magenta curve) shows a flat current up to the MPP so that the *V*_oc_ is dominated by recombination. When diffusion collection is replaced by electrical field effect on charge extraction, the current becomes linearly increasing at lower voltages. Then, the current shape is severely affected by the field collection efficiency. For the description of hysteresis effects, we apply a sinusoidal variation over the whole range of relevant voltages, as described in Section S5 of the Supporting Information. As shown in Figure 2f, the cycling produces an opening of the photocurrent curve at zero voltage, as in the experimental data of Figure 1a and Figure SI1.

We next formulate a complete impedance model able to explain the formation of a double arc alongside the occurrence of a double inductor as shown in the impedance spectra of Figure 1 and already reported in the literature.^67,68^ The surface recombination effects use two main slow variables: A voltage *v*_s_ for surface polarization (producing a variable capacitance) and a current *j*_d_ for surface controlled recombination (producing an inductor).^12–17^ This well-known model is summarized in Section S3 of the Supporting Information. We also include the new model eqs 9 and 10 for the charge collection limitations, with delay variable *v*_b_.

All the expressions and properties of the general model are listed in Table 1, including the fixed parameters used in the Figures. Note that uppercase currents *J*_i_ represent the values in equilibrium. Briefly, *v*_s_ causes delay in the low frequency capacitance *C*_s_(*v*_s_), with a relaxation time τ_s_ (eqs [Disp-formula eqT1], [Disp-formula eqT5], and [Disp-formula eqT15]), *J*_f_(*V*) is a “fast” recombination current, without kinetic limitations (eqs [Disp-formula eqT1] and [Disp-formula eqT6]); and *j*_d_ is a “slow” recombination current with stationary value *J*_d_(*V*) and the relaxation time τ_d_ (eqs [Disp-formula eqT1], [Disp-formula eqT3], and [Disp-formula eqT7]). Additionally, the charge collection expressions presented above controlled by *v*_*b*_ are shown for completeness in eqs [Disp-formula eqT1], [Disp-formula eqT2], and [Disp-formula eqT4]. Then, the model is finalized and allows the exploration of experimental current–voltage curves under working operational conditions.

**Table 1:** Variables, Parameters, and Transient and Stationary Equations of the Model

**Variables**	
External	*j*_tot_ (mA), *V* (*V*), Φ (cm^–2^ s^–1^)
Internal	*j*_d_ (mA), *v*_s_, *v*_b_(*V*)
**Simulation parameters**	
Prefactors	*J*_f0_ = 1 × 10^–9^ mA, *J*_d0_ = 5 × 10^–11^ mA, *J*_ph0_(Φ) = 15 mA
Capacitors	*C*_g_ = 10^–8^ F, *C*_s0_ = 10^–10^ F
Ideality coefficients	*n*_f_ = 4.23, *n*_d_ = 3.27, *n*_s_ = 3.85, *k*_B_ *T* = 0.026 eV
Charge collection	*V*_0_ = 0.5 V, η_d_, η_f_
Relaxation times	τ_d_, τ_b_, τ_s_ = *R*_C_ *C*_s_, τ_g_ = *R*_rf_ *C*_g_
**Time dependent equations**	
T1 $j_{\mathrm{tot}}=C_{g}\frac{dV}{dt}+J_{f}\left(V\right)+j_{d}+C_{s}\left(v_{s}\right)\frac{dv_{s}}{dt}-j_{\mathrm{ph}}\left(v_{b},\Phi \right)$	
T2 $j_{\mathrm{ph}}\left(v_{b},\Phi \right)=j_{\mathrm{ph}0}\left(\Phi \right)\left[\eta_{d}+\eta_{f}\left(1-\frac{v_{b}}{V_{0}}\right)\right]$	
T3 $\tau_{d}\frac{dj_{d}}{dt}=J_{d}\left(V\right)-j_{d}$	
T4 $\tau_{b}\frac{dv_{b}}{dt}=V-v_{b}$	
T5 $\tau_{s}\frac{dv_{s}}{dt}=V-v_{s}$	
**Stationary currents**	
T6 $J_{f}\left(V\right)=J_{f0}e^{qV/n_{f}k_{B}T}$	
T7 $J_{d}\left(V\right)=J_{d0}e^{qV/n_{d}k_{B}T}$	
**Steady-state currents**	
T8 $j_{\mathrm{tot}}=J_{f}\left(V\right)+J_{d}\left(V\right)-j_{\mathrm{ph}}\left(V,\Phi \right)$	
T9 $j_{\mathrm{ph}}\left(V,\Phi \right)=j_{\mathrm{ph}0}\left(\Phi \right)\left[\eta_{d}+\eta_{f}\left(1-\frac{V}{V_{0}}\right)\right]$	
**Impedance elements:**	*g* conductance, *R* resistance, *C* capacitance, *L* inductance
T10 $g_{\mathrm{rf}}\equiv \frac{1}{R_{\mathrm{rf}}}=\frac{qJ_{f}\left(V\right)}{n_{f}k_{B}T}$	
T11 $g_{\mathrm{rs}}\equiv \frac{1}{R_{\mathrm{rs}}}=\frac{qJ_{d}\left(V\right)}{n_{d}k_{B}T}$	
T12 $g_{b}\equiv \frac{1}{R_{b}}=\frac{J_{\mathrm{ph}0}\eta_{f}}{V_{0}}$	
T13 $L_{d}=\tau_{d}R_{\mathrm{rs}}$	
T14 $L_{b}=\tau_{b}R_{b}$	
T15 $C_{s}\left(v_{s}\right)=C_{s0}e^{qv_{s}/n_{s}k_{B}T}$	
T16 $g_{ion}=\frac{C_{s}}{\tau_{s}}\equiv \frac{1}{R_{C}}$	
**Impedance expressions**	
T17 $Z\left(\omega \right)=\left[\frac{1}{R_{\mathrm{rf}}}+i\omega C_{g}+\frac{1}{R_{C}+\frac{1}{i\omega C_{s}}}+\frac{1}{R_{\mathrm{rs}}+i\omega L_{d}}+\frac{1}{R_{b}+i\omega L_{b}}\right]$	
T18 $R_{\mathrm{DC}}={\left(\frac{1}{R_{\mathrm{rf}}}+\frac{1}{R_{\mathrm{rs}}}+\frac{J_{\mathrm{ph}0}\eta_{f}}{V_{0}}\right)}^{-1}$	

With the aim of analyzing the combination of kinetic processes, we find the expression of the IS model by the methods previously explained.^12–17^ We assume small signal variables and thus, we convert the model equations to linearized expressions using the Laplace transform method, added to the steady-state characteristic. We arrive at the expression given by eq [Disp-formula eqT17], where the admittances of recombination and polarization have been derived in Section S3 of the Supporting Information. Note that we consider, for simplicity, that the parasitic series resistance effects are negligible (*R*_s_ → 0) in comparison to the conductance states of the perovskite over the voltage range of the measurement.

The model has the structure indicated in Figure 3a. The two branches *R*_rf_ and *C*_g_ produce “fast” response, with the standard time constant *τ*_*g*_ = *R*_rf_ *C*_*g*_ of the high frequency arc, while the three internal variables generate additional parallel branches in longer time scales: two chemical inductors for *j*_*d*_, *v*_*b*_, and one RC line for *v*_*s*_. The separate components are amply reported in the literature^12–17^ and here we provide an integrated approach to all these processes that occur in halide perovskite photovoltaic devices.

**Figure 3:**
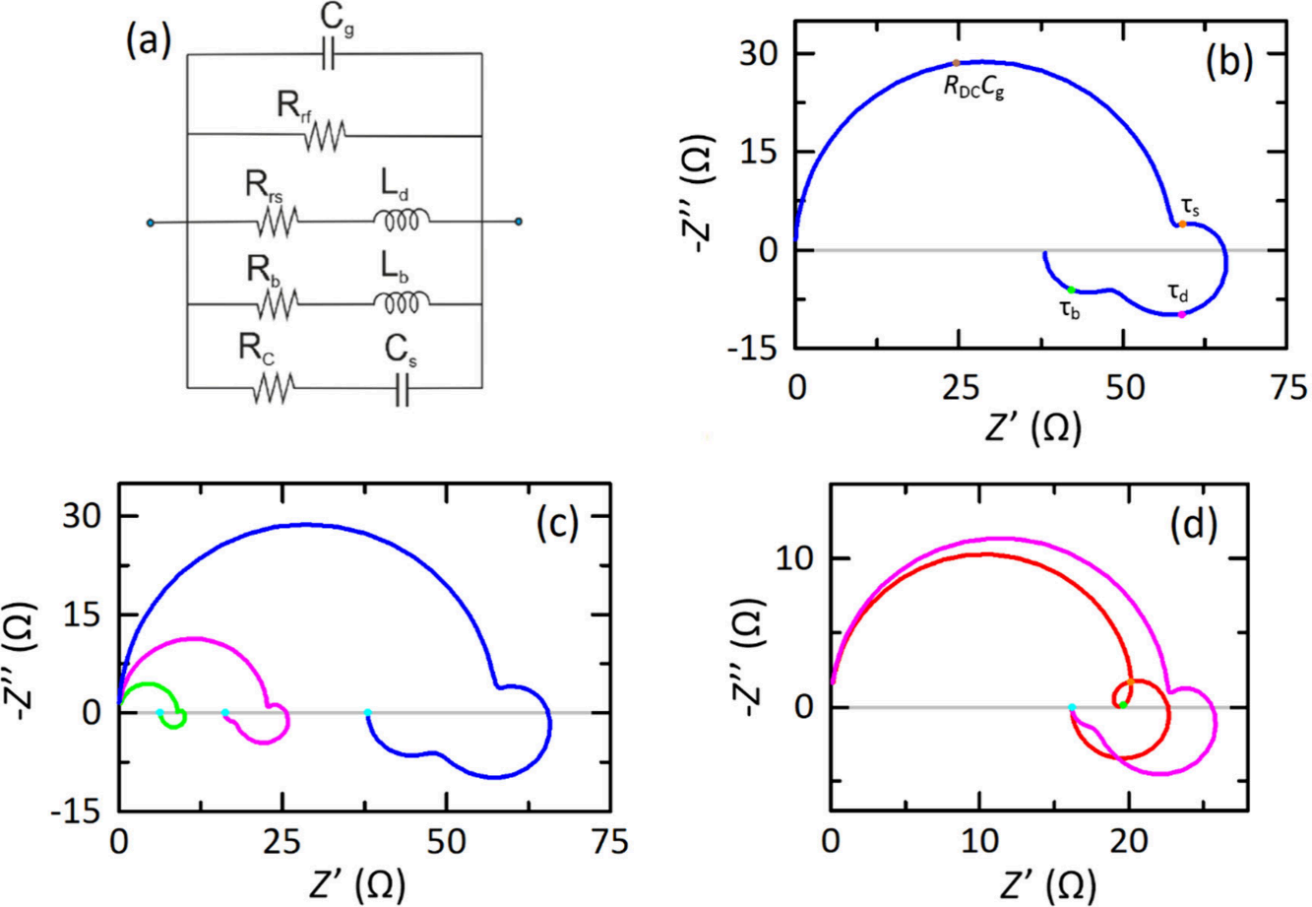
(a) Equivalent circuit model for the impedance. (b) Simulation of impedance spectrum with time constants τ_*s*_ = 0.5 ms, τ_d_ = 3 ms, τ_b_ = 60 ms and parameters of Table 1 with *J*_ph0_ (Φ) = 15 mA and *V* = 0.8 V. The time constants corresponding to different characteristic frequencies are indicated. (c) Impedance spectra resulting from modification of voltage: *V* = 0.9 V (pink) and *V* = 1.0 V (green), compared to (b) in blue. (d) Spectrum for τ_*s*_ = 0.5 ms, τ_d_ = 3 ms, τ_b_ = 60 μs (red), compared to the impedance plot of (c) obtained with *V* = 0.9 V. The cyan point is the DC value of the impedance (*R*_DC_) that is the same in both cases.

On the one hand, *R*_rs_-*L*_d_ branch of the equivalent circuit shown in Figure 3a corresponds to the classical chemical inductor structure associated with a delayed-controlled surface recombination current.^44^ The branch formed by *R*_b_ and *L*_b_ is, on the other hand, a new arrangement that describes ion-induced charge collection efficiency losses, connected to bulk recombination. One spectrum containing all the processes is simulated in Figure 3b. In addition to the usual double arc feature associated with the capacitances *C*_g_ and *C*_s_, in the first quadrant, the salient feature is the appearance of two chemical inductor processes in the fourth quadrant, beyond the simple and unitary feature described in literature. As already mentioned, the double inductor is experimentally observed,^67,68^ as shown in Figure 1e and Figure SI2. The double inductor is obtained only at moderate voltages due to the limitation of measurement at lower voltages; see Section 2 of the Supporting Information.

The spectral shape of the impedances is determined by the ordering of the characteristic times,^69^ that in Figure 3b is {τ_g_, τ_s_, τ_d_, τ_b_}, in increasing sequence. Figure 3c shows the changes of impedance spectra with the applied voltage. In the red spectrum of Figure 3d, the ordering has been swapped to {τ_g_, τ_b_, τ_s_, τ_d_}. Now the inductor corresponding to τ_b_ occurs at higher frequency than the low frequency capacitive arc, causing the inductive loop that is frequently observed in halide perovskite devices, as shown in Figure 3d.^59^ When the voltage is changed, parameters are modified and spectra can change drastically. If *L*_*b*_ = 0 only a resistor remains so that τ → 0. For the capacitor branch, usually interpreted as ionic conductance and polarization, *τ*_*s*_ = g_C_^–1^ *C*_*s*_, so that τ → 0 is obtained by large conductance or small capacitance.

In the following, we briefly discuss the limitations and possible improvements of the present model. Experimental impedance spectra of halide perovskite solar cells display a wide variety of behaviors. This diversity is natural, given the enormous range of compositions, morphologies, contact architectures, and environmental conditions that govern device operation and enable chemical exchanges. In this work we interpreted the bulk inductive contribution, added to surface recombination components, using a simplified single-voltage model. In practice, specific experiments often require different combinations of series and parallel subcircuits that separately capture bulk and interfacial effects, as illustrated in Figure SI9 and in earlier studies.^17,45,59,70^ Furthermore, the situation is often more complex than a simple series ordering of elements, and the experimental identification of mechanisms relies on correlations among fitted parameters. For example, the surface ionic accumulation may extend into the bulk; the seminal study of Garcia-Belmonte^71^ showed that the low-frequency interfacial capacitance combines double-layer charging with ionic transport. Transport layers may also introduce overlapping time constants that interfere with interpretation.^72,73^ Ionic buildup can either reinforce the internal drift field or create unfavorable reversed fields at interfaces, where carriers become trapped and recombine through surface states.^74^ Such situations can only be captured by spatially resolved device models,^75–79^ which describe Fermi-level distributions and carrier transport using drift–diffusion and Poisson equations with proper boundary conditions to the transport layers. These approaches provide a consistent picture of ionic–electronic mixed conduction, charge compensation, and their impact on recombination, as further discussed in Section S7 of the Supporting Information. In addition, electrochemical and photochemical reaction pathways—including the rich defect chemistry of halide perovskites^80–82^ —must be considered to fully understand the dynamics. These methods are particularly valuable for degradation studies, as they enable exploration of the full landscape of hysteresis phenomena where contacts, recombination, and ionic motion are tightly coupled.

In summary, mobile ions in perovskite solar cells affect stationary performance through two primary mechanisms: (i) enhanced recombination, lowering the photovoltage and (ii) reduced charge collection, lowering photocurrent. The first mechanism has been widely modeled in the literature; here, we focused on the second. By combining theoretical modeling and numerical simulations, we demonstrated the signatures of ion-induced field-screening in both current–voltage scans and impedance spectra. In *jV* scans, these appear as normal or inverted or hysteresis near *j*_*sc*_. In impedance spectroscopy, they manifest as low-frequency capacitive and inductive features: recombination-driven arcs (*R*_rs_-*L*_d_) and field-screening arcs (*R*_b_-*L*_b_). Classification and ordering of relaxation times, together with resistances, provide general criteria for predicting impedance spectra and associated hysteresis. This methodology connects distinct hysteresis types in perovskite solar cells with their impedance signatures, and offers a diagnostic framework for identifying degradation pathways that most critically affect device performance.

## Supplementary Material





## Data Availability

The data presented here can be accessed at 10.5281/zenodo.16880157 (Zenodo) under the license CC BY 4.0 (Creative Commons Attribution 4.0 International).

## Appendix

Consider the expression of the current in a photovoltaic device

11
$$j=\frac{dQ_{s}}{dt}$$

Here *Q*_*s*_(*x*) is a charge function that depends on an internal variable *x*. Hence

12
$$j=C_{a}\left(x\right)\frac{dx}{dt}$$

where *C*_a_ = d*Q*_*s*_/d*x*. This internal variable depends on the applied voltage *V* by the equation

13
$$\tau_{s}\frac{dx}{dt}=F\left(V\right)-x$$

In equilibrium it is *x̅* = *F*(*V̅*). The *x* depends on voltage through the nonlinear function *F*. The small signal ac version of ([Disp-formula eq12]) and ([Disp-formula eq13]) is

14
$$ĵ=i\omega C_{a}\mathrm{x̂}$$

15
$$\tau_{s}i\omega \mathrm{x̂}=f\left(\mathrm{V̅}\right)\mathrm{V̂}-\mathrm{x̂}$$

Here *f* = d*F*/d*V*, *x̅* is the steady state value, and *x̂* the small expansion value. Hence the impedance is

16
$$Z=\frac{\mathrm{V̂}}{ĵ}=\frac{1}{g_{C}}+\frac{1}{C_{s}i\omega }$$

This is a series *RC* circuit with the conductance and capacitance

17
$$g_{C}=\frac{C_{s}}{\tau_{s}},,C_{s}=fC_{a}$$

If we add to eq 11 the recombination current *J*_*rec*_(*V*), a parallel recombination resistance *R*_*rec*_ = (d*J*_*rec*_/d*V*)^−1^ will be obtained.

Now we make a different assumption with respect to ([Disp-formula eq11]). The current has the form

18
$$j=j_{a}\left(x\right)$$

for a nonlinear function *j*_*a*_ and an internal variable *x* also controlled by eq 13. For a small expansion we have

19
ĵ=m(x̅)x̂

where *m*(*x*) = d*j*_*a*_/d*x*. In combination with ([Disp-formula eq15]), we find the impedance expression

20
$$Z=\frac{1}{g_{L}}+L_{b}i\omega$$

This is a series of a resistor and inductor, with the values

21
$$g_{L}=mf,,L_{b}=\frac{\tau_{s}}{mf}$$

The delay equation ([Disp-formula eq13]) causes hysteresis, which is associated with the transient behavior of the current under a voltage step *V̅* → *V̅* + Δ*V*_*ap*_.^46^ Integration of eq 13 gives^13^

22
$$\Delta v_{s}=f\Delta V_{ap}\left(1-e^{-t/\tau_{s}}\right)$$

For the capacitive system ([Disp-formula eq11]) we obtain

23
$$\Delta j\left(t\right)=g_{C}\Delta V_{ap}e^{-t/\tau_{s}}$$

For the inductive system ([Disp-formula eq18]) it is

24
$$\Delta j\left(t\right)=g_{L}\Delta V_{ap}\left(1-e^{-t/\tau_{s}}\right)$$

## Notes

Experimental section; double inductor in the dark, *J–V* curves, impedance plot; derivation of the surface recombination model, *J–V* curves, impedance spectra, conductances, relaxation times; relaxed and unrelaxed current–voltage characteristics, conduction band profiles, current–voltage characteristics, model parameters, effect of ramp speed on the *j*–*V* trajectory; voltage cycling and hysteresis, numerical simulation plots, model parameters; interpretation of voltages and model assumptions, impedance models, energy band diagrams; discussion on numerical methods (PDF)
Transparent Peer Review report available (PDF)
