# Supplementary material for: Dynamic Screening and the Chemical Inductor of Perovskite Solar Cells: From J–V Transients to Impedance Spectroscopy
Source: J Phys Chem Lett. 2025 Oct 15;16(42):11053–63. doi: 10.1021/acs.jpclett.5c01916 (PMC12557368; doi:10.1021/acs.jpclett.5c01916)
Supplement: Supplementary file 1 [file jz5c01916_si_001.pdf]

Supporting information

Dynamic Screening and the Chemical Inductor of Perovskite Solar Cells: From J–V Transients to Impedance Spectroscopy

Enrique H. Balaguera[1,*] Elnaz Ghahremani Rad[2], Alexander R. Uhl[2], Antonio Guerrero[3] and Juan Bisquert[4,*]

[1] Escuela Superior de Ciencias Experimentales y Tecnología (ESCET), Universidad Rey Juan Carlos, 28933 Móstoles, Madrid, Spain

[2]Laboratory for Solar Energy & Fuels (LSEF), School of Engineering, University of British Columbia, Kelowna, V1V 1V7, Canada

[3]Institute of Advanced Materials (INAM), Universitat Jaume I, 12006 Castelló, Spain

[4]Instituto de Tecnología Química (Universitat Politècnica de València-Agencia Estatal Consejo Superior de Investigaciones Científicas), Av. dels Tarongers, 46022 València, Spain

**Corresponding author e-mail:* enrique.hernandez@urjc.es, jbisquer@itq.upv.es

Contents

S1. Experimental..1
S2. Double inductor in the dark..2
S3. Derivation of the surface recombination model..5
S4. Relaxed and unrelaxed current-voltage characteristics. 6
S5. Voltage cycling and hysteresis..13
S6. Interpretation of voltages and model assumptions..15
S7. Discussion on numerical methods..19

S1. Experimental

Fig. 1: The fabrication of FTO/NiO/MeO-2PACz/($Cs_{0.05}FA_{0.8}MA_{0.15}PbI_{2.75}Br_{0.25}$) /PCBM/BCP/Au/Ag perovskite solar cells involves multiple precise steps. First, FTO glass substrates are cleaned, blow-dried, and treated with UV-ozone. A NiO_x hole transport layer is spin-coated and annealed in two stages. In a nitrogen-filled glovebox, a MeO-2PACz interfacial layer is applied and annealed. Then, a mixed-cation, mixed-halide perovskite solution is prepared, filtered, and deposited via a three-step spin-coating process with chlorobenzene as an anti-solvent, followed by annealing. An electron transport layer (PCBM) and a BCP layer are then sequentially spin-coated. Finally, a metal electrode layer (Au/Ag) is thermally evaporated under high vacuum to complete the

device. The current density-voltage curves for the perovskite solar cells were measured using a Keithley 2401 source meter under a standard AM1.5G Sciencetech Solar Simulator (Class AAA solar simulation). The lamp was calibrated using a standard Si photodiode. The mask area was 0.16 cm^2 and the cells were measured at room temperature. The scan rate of 100 mV/s was initially used but the samples were measured at different scan rates as well.

S2. Double inductor in the dark

For scan rate dependence *J-V* curves (Fig. SI1) and impedance spectroscopy measurements (Fig. SI2) the devices were fabricated following the protocol of reference [1] with the configuration FTO/SnO_2/Perovskite/Spiro-OMeTAD/Au with the SnO_2 layer prepared by atomic layer deposition (ALD). The perovskite layer presents a mixed formulation to enhance absorption and stability containing Cs/MA/FA/Pb/I/Br. These devices show power conversion efficiency η = 18.3 %. Measurements under dark conditions were carried out using a potentiostat equipped with a frequency analyzer module (Autolab PGSTAT30). Devices were measured according to the following sequence of experiments: *J-V* as a function of the scan rate under dark conditions, *J-V* as a function of the scan rate under 1 sun, illumination at Voc conditions during 20 minutes and IS under dark conditions.

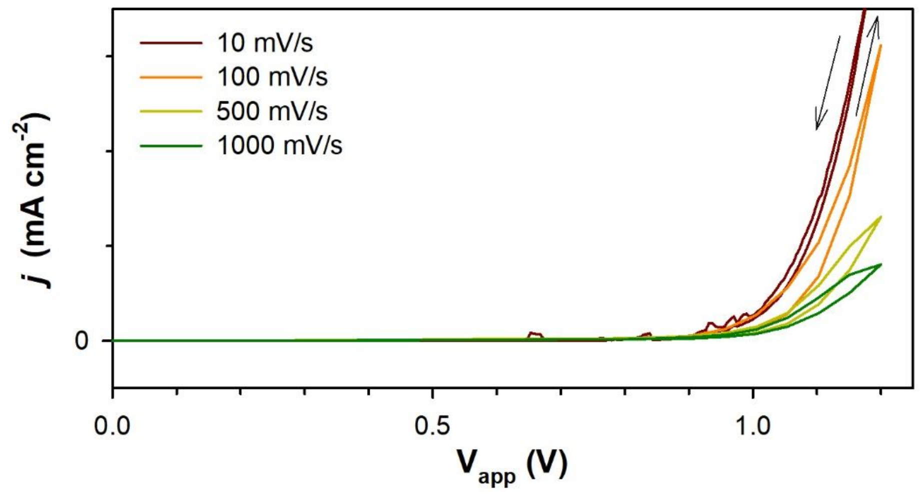

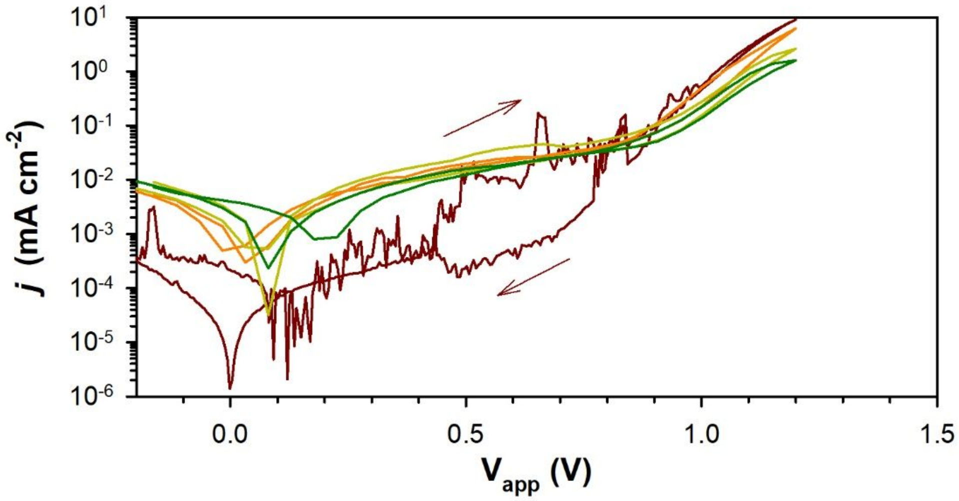

Fig. SI1. *J-V* response of a perovskite solar cell FTO/SnO_2/Perovskite/Spiro-OMeTAD/Au with the SnO_2 layer prepared by atomic layer deposition (ALD) measured under dark conditions and as a function of the scan rate. Linear (top) and semi-log (bottom) scale graphs are represented for clarity.

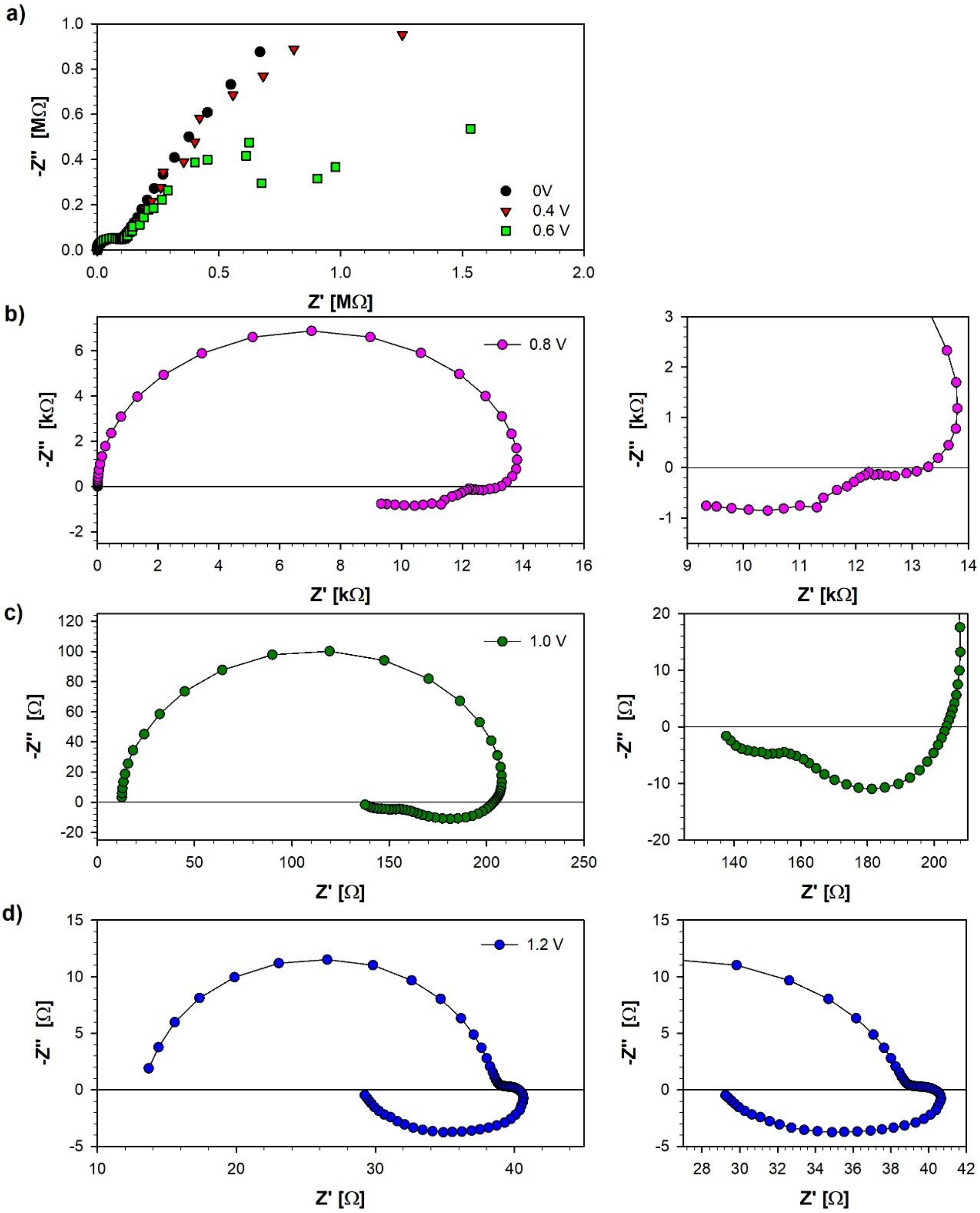

Fig. SI2. Complex impedance plot of measurements of a perovskite solar cell FTO/SnO_2/Perovskite/Spiro-OMeTAD/Au with the SnO_2 layer prepared by atomic layer deposition (ALD) measured in the dark under different DC voltages with. a) V_{DC} = 0 V, 0.4 V and 0.6 V, b) V_{DC} = 0.8 V, c) V_{DC} = 1.0 V and d) V_{DC} = 1.2 V. The insets on the right focus on the inductive features.

We remark that impedance of perovskite solar cells is rarely reported below 0.5 V, since the recombination vanishes and the recombination resistance is huge. In Fig. 1, the

inductor is observed at 0.8 V, before the onset of large recombination. The same in Fig. SI2. In both cases the inductor feature in the impedance corresponds to the region of hysteresis of the photocurrent. Obtaining the inductor at lower voltages may require specific experimental methods adapted to the purpose.

S3. Derivation of the surface recombination model

The recombination-polarization part of our model is formulated as follows:[2-7]

$$\sum j_{\text{rec-pol}}(v_s, j_d) = \underbrace{J_f(V) + j_d}_{j_{\text{rec}}} + \underbrace{\frac{dQ_s(v_s)}{dt}}_{j_{\text{pol}}} \tag{1}$$

$$\tau_d \frac{dj_d}{dt} = J_d(V) - j_d \tag{2}$$

$$\tau_s \frac{dv_s}{dt} = V - v_s \tag{3}$$

that leads to the summatory of admittance functions:

$$\sum Y_{\text{rec-pol}} = \sum \frac{\hat{j}_{\text{rec-pol}}}{\hat{V}} = \underbrace{g_{\text{rf}} + \frac{1}{\frac{1}{g_{\text{rs}}} + i\omega L_d}}_{\hat{j}_{\text{rec}}/\hat{V} = (\hat{j}_f + \hat{j}_d)/\hat{V}} + \underbrace{\frac{1}{\frac{1}{g_{\text{ion}}} + \frac{1}{i\omega C_s}}}_{\hat{j}_{\text{pol}}/\hat{V}} \tag{4}$$

Based on experimental observations in literature, we assume the exponential dependences in the variables of eqs (1-3) as eqs T6, T7, and

$$\frac{dQ_s dv_s}{dv_s\, dt} = C_s(v_s)\frac{dv_s}{dt} \tag{5}$$

leading to the circuit elements of Figure 3a with the forms indicated in Table 1. The total external current j_{tot} is

$$j_{\text{tot}} = C_g \frac{dV}{dt} + \sum j_{\text{rec-pol}}(v_s, j_d) - j_{\text{ph}}(v_b, \Phi) \tag{6}$$

where the first term represents the charging of the dielectric capacitance C_g of the active layer and the summatory of recombination-polarization currents $\sum j_{\text{rec-pol}}(v_s, j_d)$ corresponds to the standard model developed previously.[2-7] This model with one capacitor and one inductor associated to surface polarization and recombination is shown in Fig. SI3.

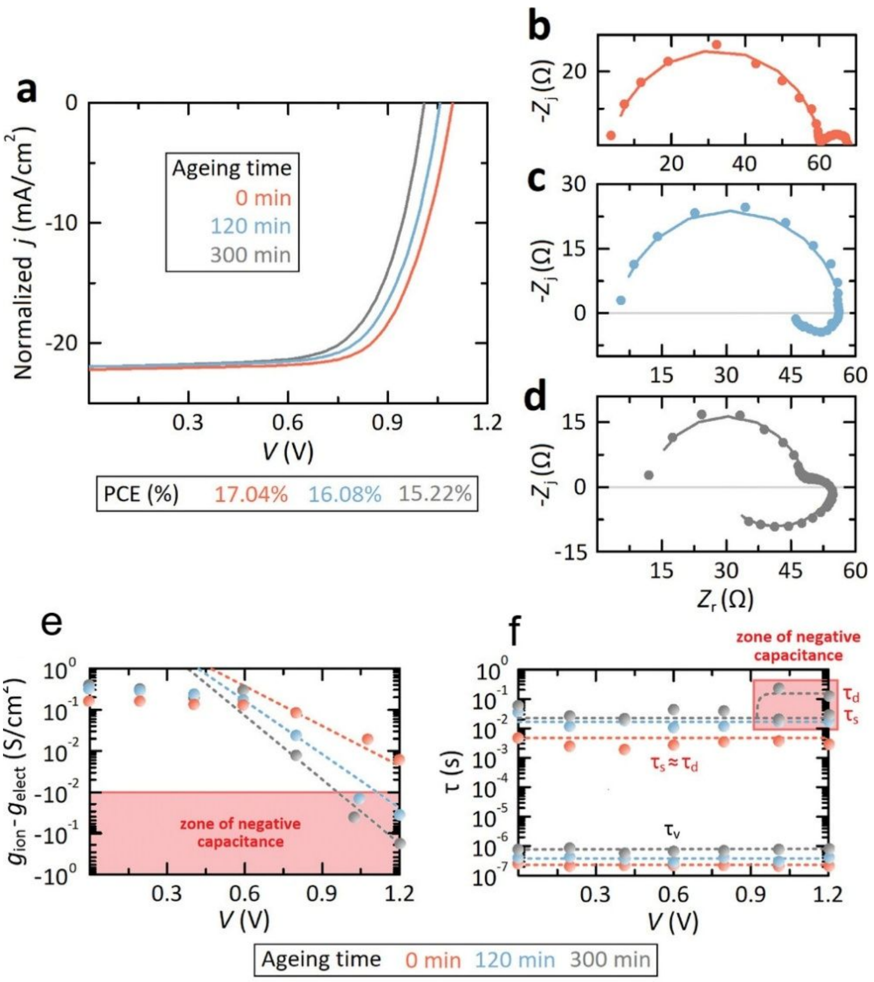

Fig. SI3. a) Representative stabilized current–voltage (*j*–*V*) curves at slow scan rates (from 5 to 1 mV s^{-1}) measured on a pristine perovskite solar cell (based on the multi-cation absorber of nominal formulation $Rb_{0.05}Cs_{0.05}FA_{0.75}MA_{0.15}Pb_{1.05}(I_{0.95}Br_{0.05})_3$ with the H2pp molecule) as well as after continuous illumination for the specified time. Note that the *j*–*V* curves are normalized at 0 V. b–d) Impedance spectra as a function of ageing time. Parameters: PCE is the power conversion efficiency, and Z_r and Z_j are the real and imaginary parts of the impedance, respectively. e) Relation of ionic/electronic conductances, $g_{ion} - g_{rec}$, and (f) relaxation times (τ_v, τ_s, and τ_d) extracted from the transient responses throughout the voltage range V for different ageing states. Note that shunt resistance effects are visualized at low voltages in (e). Reproduced from H. Balaguera, E.; Bisquert, J., *Small* **2024**, *21*, 2409534, licensed under a Creative Commons Attribution 4.0 International License (CC BY 4.0).[3]

S4. Relaxed and unrelaxed current-voltage characteristics.

In solar cells with selective contacts, the built-in potential V_{bi} arises from the difference of work functions between the contacts. If mobile ions are present in the semiconductor or at interfaces, they can redistribute and form Helmholtz (double) layers (HL) at the

interfaces. These layers behave like capacitors and can store part of the built-in electric field. As a result, only part of the total contact potential difference, V_{tot}, drops across the semiconductor bulk, V_{drift}, while the remainder appears across the HLs. This redistribution is called electrostatic screening.

The semiconductor layer and the two HLs can be modeled as three capacitors in series. The same charge Q flows through all three elements. If their capacitances per unit area are $C_{HL,L}$, C_{sc} and $C_{HL,R}$ respectively, the voltage across each element is:

$$V_i = \frac{Q}{C_i} \tag{7}$$

$$V_{tot} = V_{HL,L} + V_{drift} + V_{HL,R} \tag{8}$$

It follows that the voltage across the semiconductor is only a fraction f_{sem} of the total electrostatic voltage:

$$f_{sem} = \frac{\frac{1}{C_{sc}}}{\frac{1}{C_{HL,L}} + \frac{1}{C_{sc}} + \frac{1}{C_{HL,R}}} \tag{9}$$

As the applied voltage increases in forward bias, the total electrostatic drop $V_{tot} = V_{bi} - V_{app}$ decreases. If the capacitances are constant, the fraction f_{sem} is constant, so the voltage across the Helmholtz layers scales down proportionally with V_{tot} This means that the voltage on the Helmholtz layers decreases as the applied voltage increases.

Undershoot case

The division of the total built-in potential affects the internal electric field inside the semiconductor, caused by V_{drift}. A weaker internal field reduces carrier drift, which in turn lowers carrier collection, especially if the diffusion length is shorter than the layer thickness, $L_d < L$, i.e. when collection depends strongly on drift. Therefore, screening by Helmholtz layers reduces the short-circuit current j_{sc} and the open-circuit voltage V_{oc}.

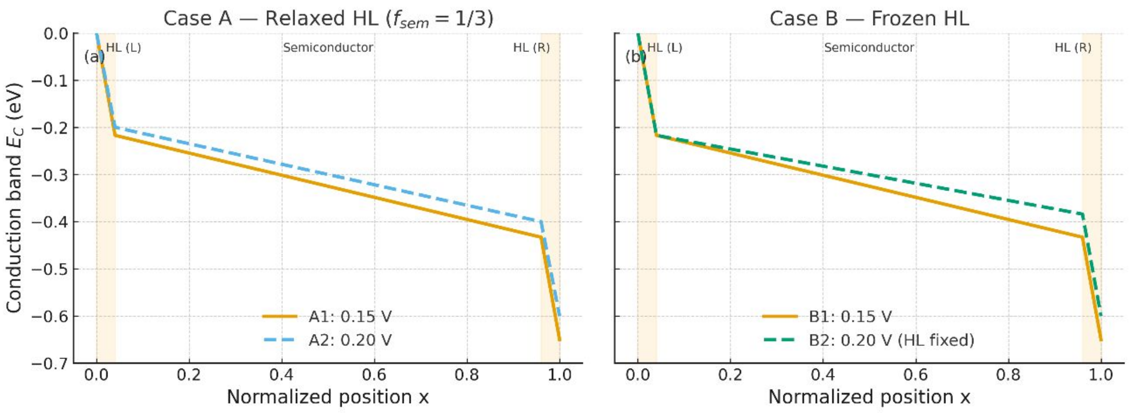

Fig. SI4. Conduction band profiles of a selective-contact solar cell including Helmholtz layers at the interfaces. The horizontal axis shows the normalized device thickness, with shaded regions indicating the left and right Helmholtz layers (HL) and the central region

corresponding to the semiconductor bulk. The vertical axis is the conduction band energy E_C (eV), plotted between –1.0 and 0.0 eV. (a) Case A — Relaxed HL (capacitive partition with $f_{sem} = 1/3$): profiles are shown for applied voltages V_{app} = 0.15 V (solid line, A1) and 0.20 V (dashed line, A2). Both the HL and semiconductor drops are reduced proportionally when the applied voltage increases. (b) Case B — Frozen HL (HL potential fixed at its 0.15 V value): profiles are shown for V_{app} = 0.15 V (solid line, B1) and 0.20 V (dashed line, B2). The HL potential remains constant while the semiconductor absorbs the entire additional applied voltage.

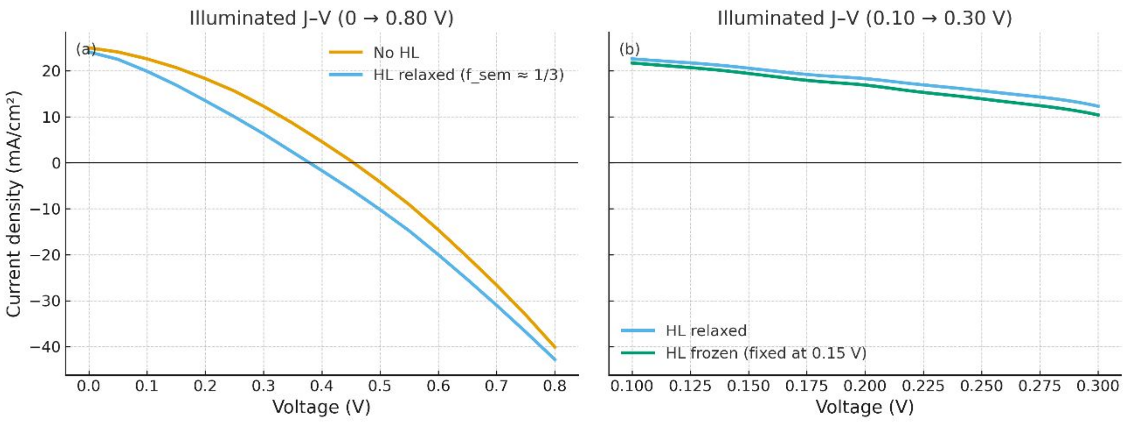

Fig. SI5**.** Illuminated current–voltage characteristics of a selective-contact solar cell under different assumptions for the Helmholtz layers (HL). All currents are given in mA cm^{-2}. (a) Full voltage range (0 → 0.80 V), comparing the case without Helmholtz layers (orange), in which the full electrostatic drop is inside the semiconductor, and the case with relaxed Helmholtz layers (blue), where the applied bias is shared between the HL and the semiconductor according to capacitive partition ($f_{sem} \approx 1/3$). (b) Zoomed voltage range (0.10 → 0.30 V), comparing relaxed HL (blue) with frozen HL (green). In the relaxed case, the HL potential adapts at each voltage, while in the frozen case the HL potential is held fixed to its value at 0.15 V, so the semiconductor absorbs the entire additional voltage increment. The curves are smoothed interpolations of the calculated data.

We can distinguish two limiting cases regarding the response of the Helmholtz layers to changes in applied voltage:

- **Relaxed Helmholtz layers:** the ions are mobile and respond instantly to applied bias. At every applied voltage, they rearrange so that the same capacitive division applies. As forward bias increases, the total internal drop decreases, and the voltage across the Helmholtz layers decreases proportionally. The semiconductor sees only a fraction f_{sem} of the total voltage change.
- **Frozen Helmholtz layers:** the ionic distribution cannot change on the timescale of the voltage change. The Helmholtz voltage stays fixed at its initial value (for example, the value at 0.15 V). Any further change in applied voltage drops entirely across the semiconductor. This causes a smaller electrical field than before.

Let us consider the transient response.

When the applied voltage is changed suddenly (for example, a step from 0.15 V to 0.20 V, as in Fig. SI5), the response of the current depends on whether the Helmholtz layers can adapt:

- Immediately after the step (frozen ions): the voltage across the HLs does not change. The semiconductor takes the full step, its internal field decreases more strongly. As a result, the photocurrent drops below the steady-state value in Fig. SI5b.
- Over time (ion relaxation): mobile ions rearrange and take up part of the applied voltage. This reduces the share of the step falling across the semiconductor, the internal field partially recovers, and recombination is reduced. Consequently, the current rises gradually to the relaxed steady-state value.

The net result is an undershoot of the current below the equilibrium (relaxed) jV line.

Model Parameters

Geometry & Electrostatics

Parameter	Value	Notes
Film thickness L	3.0 μm	chosen > L_D
Relative permittivity ε_r	11.7	silicon-like
Vacuum permittivity ε_0	8.85×10^{-12} F/m	constant
Semiconductor capacitance $C_{sc} = \varepsilon/L$	$\approx 1.0\times10^{-8}$ F/cm²	used for partitioning
Helmholtz capacitances $C_{HL,L} = C_{HL,R}$	same as C_{sc}	gives $f_{sem} \approx 1/3$
Built-in potential V_bi	0.8 V	difference of contact work functions

Charge Transport

Parameter	Value
Temperature T	300 K
Intrinsic carrier density n_i	1×10^{10} cm^{-3} (converted to m^{-3})
Electron mobility μ_n	10 cm²/V·s
Hole mobility μ_p	5 cm²/V·s
Electron lifetime τ_n	50 ns
Hole lifetime τ_p	50 ns
Diffusion constants	from Einstein relation $D = \mu V_T$
Electron diffusion length L_{Dn}	≈1.1 μm
Hole diffusion length L_{Dp}	≈0.8 μm
Note	$L_D < L$ so collection depends on electric field

Contacts	
Parameter	Value
Majority surface recombination velocity S_{maj}	10^7 cm/s
Minority surface recombination velocity S_{min}	10^2 cm/s
Left contact	electron-selective ($S_n = S_{maj}$, $S_p = S_{min}$)
Right contact	hole-selective ($S_n = S_{min}$, $S_p = S_{maj}$)
**Optical Generation**	
Parameter	Value
Profile	Beer–Lambert: $G(x) \propto e^{-\alpha x}$ from left
Absorption coefficient α	10^5 cm^{-1}
Calibration	Adjusted so $j_{sc} \approx 25$ mA/cm² at V=0 in relaxed case, reused unchanged
**Two Helmholtz Scenarios**	
Case	Description
Relaxed HL	At each bias, HL and semiconductor share $V_{total} = V_{bi} - V$ in fixed ratio: $f_{sem} \approx 1/3$
Frozen HL	HL voltage locked to its value at V = 0.15 V; any further voltage change goes entirely to the semiconductor

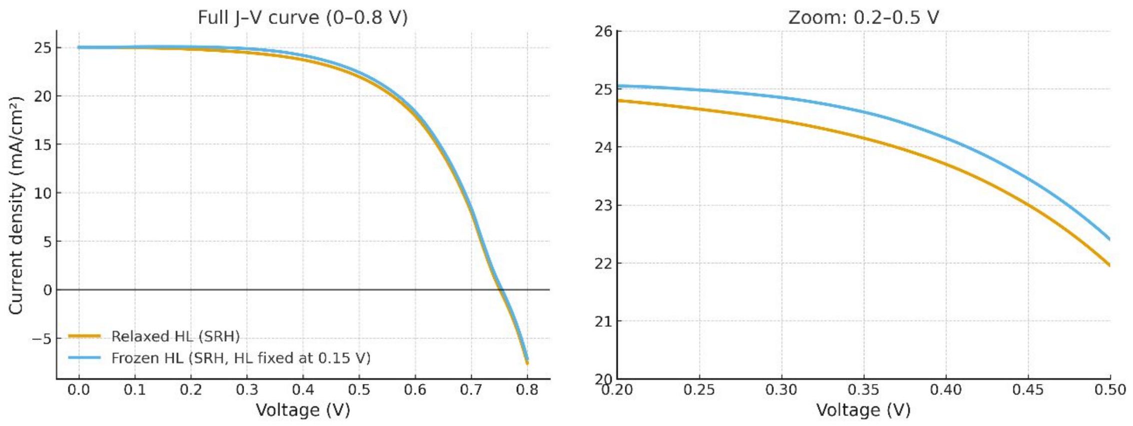

Figure SI6. Drift–diffusion current–voltage characteristics including SRH recombination and Helmholtz layers at the contacts. Cubic interpolation (PCHIP) of the simulated data. The relaxed case (orange) corresponds to mobile ions and Helmholtz layers adapting at each bias, so that the applied voltage is partitioned between the interfacial and bulk capacitances ($f_{sem} \approx 1/3$). The frozen case (green) corresponds to Helmholtz potentials

fixed at their values at 0.15 V, so the semiconductor absorbs the full incremental bias. (a) Full J–V curves over 0–0.8 V, calibrated to give $j_{sc} \approx 25$ mA cm^{-2} and $V_{oc} \approx$ 0.75 V in the relaxed case. (b) Zoomed view of the interval 0.20–0.50 V, where the frozen case lies systematically above the relaxed case, producing the characteristic current overshoot associated with the chemical inductor effect.

Overshoot case

The calculation uses the same drift–diffusion–Poisson method as in the previous case, with identical geometry, contacts, mobilities, and Helmholtz-layer partition rules. The only change is in the recombination law: instead of band-to-band (lifetime) recombination, Shockley–Read–Hall (SRH) recombination is implemented. The recombination rate is given by:

$$U_{SRH} = \frac{np - n_i^2}{\tau_p(n+n_1)+\tau_n(p+p_1)} \tag{10}$$

with $\tau_n = \tau_p$= 50 ns, $n_1 = p_1 = n_i$ and n_i = 10^{10} cm^{-3}. This expression captures the effect of trap-limited recombination kinetics, which is widely observed in perovskite and related semiconductors.

The treatment of Helmholtz layers is unchanged: in the relaxed case the semiconductor drop is a constant fraction $f_{sem}\approx$ 1/3 of the total internal potential; in the frozen case the HL voltage is fixed at its value for V_{app}= 0.15 V, and the semiconductor absorbs the entire incremental bias. The result is shown in Fig. SI6.

The physical interpretation differs depending on the recombination mechanism considered:

• Band-to-band (radiative, lifetime-limited): The operating point is collection-limited. When the HL are frozen, the collection field inside the semiconductor is reduced. Because the diffusion length is shorter than the layer thickness, drift is essential for extraction. Reducing the field lowers the collection efficiency, and the current falls below its steady-state value. This produces an undershoot transient.

• SRH recombination: The operating point is recombination-limited. Here the frozen HL alter the band bending and carrier distributions in such a way that the effective SRH rate decreases. This happens because (i) minority-carrier densities in the critical regions are lowered, reducing the numerator $(np - n_i^2)$ and/or (ii) the denominator increases via the $(n + n_1)$ or $(p + p_1)$ terms, effectively lengthening the carrier lifetime. The reduction of recombination outweighs the small reduction of the collection field, leading to a higher current in the frozen state. The transient therefore exhibits an overshoot: the current is larger immediately after the voltage step than in the relaxed steady state. This is shown in Fig. SI7.

Thus, the sign of the effect depends on what limits performance: in collection-limited (band-to-band) operation, frozen HL reduce the field and produce an undershoot, whereas in recombination-limited (SRH) operation, frozen HL reduce the recombination rate and

produce an overshoot.

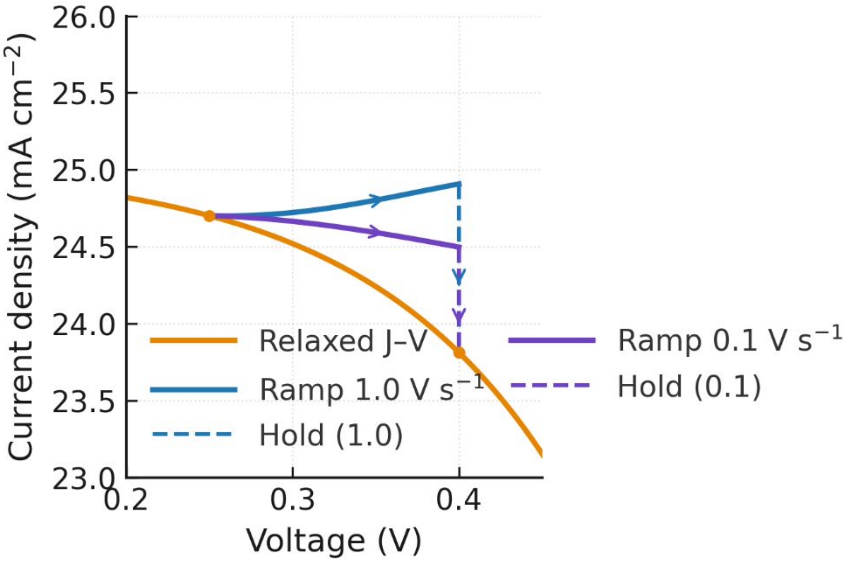

Fig. SI7. Effect of ramp speed on the j–V trajectory with Helmholtz-layer relaxation. The orange line shows the relaxed steady-state j–V curve. Blue traces: fast voltage ramp (1.0 V s^{-1}) from 0.25 to 0.40 V (solid line), followed by a hold at 0.40 V (dashed line). Purple traces: slower ramp (0.1 V s^{-1}) and subsequent hold. Arrows indicate the direction of evolution. In the fast-ramp case the Helmholtz layers lag behind the applied voltage, so the current overshoots above the relaxed curve before relaxing back during the hold. In the slower ramp, the ionic relaxation keeps pace with the voltage change, so the trajectory remains much closer to the relaxed j–V curve. This demonstrates how the apparent hysteresis depends on the timescale of the bias protocol relative to the ionic relaxation time.

Calculation of the overshoot

The calculation of Fig. SI7 extends the drift–diffusion framework by introducing a dynamic response of the Helmholtz (HL) layers under an applied voltage ramp. Instead of assuming an instantaneous voltage step, the external bias is applied as a ramp:

$$V_{app} = V_0 + s\,t\,,\ \text{for}\ \ 0 \le \mathrm{t} \le\ (\mathrm{V_F} - \mathrm{V_0})/\mathrm{s} \tag{11}$$

Where V_0 is the initial voltage, V_f is the final voltage, and s is the ramp speed. This representation allows us to capture the interplay between the timescale of the bias protocol and the relaxation of ionic charge.

The Helmholtz potential $u(t)$ follows a first-order relaxation law toward its instantaneous equilibrium value $u_{eq}(t)$

$$\tau_{HL}\frac{du}{dt} = u_{eq}(t) - u(t) \tag{12}$$

where τ_{HL} is the ionic relaxation time. The equilibrium Helmholtz potential is given by the capacitive partition:

$$u_{eq}(t) = (1 - f_{sem})(V_{bi} - V_{app}(t)) \quad (13)$$

with V_{bi} the built-in voltage and $f_{sem} \approx 1/3$ the fraction of the electrostatic potential that drops across the semiconductor bulk. The semiconductor potential drop is then $V_{sem} = V_{bi} - V_{app}(t) - u(t)$, and the effective fraction seen by the diode equation is $f_{eff}(t) = V_{sem}(t)/(V_{bi} - V_{app}(t))$,

The current density is computed using the compact diode-like proxy derived from drift–diffusion with SRH recombination:

$$j(t) = j_{ph} - j_0\left[\exp\left(\frac{f_{eff}(t)V_{app}(t)}{n\,V_T}\right) - 1\right] \quad (14)$$

where $j_{ph}$is the photocurrent, j_0 is the recombination prefactor, n is the ideality factor, and V_T is the thermal voltage. This expression captures the influence of the evolving HL partition on the recombination current.

The combined action of the external ramp and the delayed HL response produces transient deviations from the steady-state jV curve, which manifest as hysteresis-like features. The key observations are:

• For a fast ramp ($s \gg 1/\tau_{HL}$), the HL cannot keep pace with the applied bias. The semiconductor therefore experiences a larger share of the voltage, which reduces recombination and yields a higher current than the relaxed steady-state curve. This produces an apparent overshoot.

• When the voltage is held at the final value, the HL layers gradually relax, transferring part of the potential away from the semiconductor. As recombination increases, the current decays toward the steady-state value on the relaxed jV curve.

• For a slow ramp ($s \ll 1/\tau_{HL}$), the HL distribution has sufficient time to adapt at each bias point. The trajectory remains close to the relaxed J–V line, and the overshoot is negligible.

These results explain the dependence of hysteresis on the bias scan rate observed in perovskite solar cells and other ionic semiconductors. At fast scans, the ionic double layers cannot follow the voltage changes, leading to transient current deviation. At slow scans, the system remains closer to equilibrium and hysteresis is suppressed.

S5. Voltage cycling and hysteresis

For the description of hysteresis effects, we apply a sinusoidal variation over the whole range of relevant voltages, namely

$$V(t) = V_{\mathrm{m}} + V_{\mathrm{p}}\sin(\Omega t + \phi) \quad (6)$$

where V_{p} is the amplitude of the voltage, V_{m} is a reference value, Ω is the angular frequency in units of rad/s ($\Omega = 2\pi f_{\Omega}$ with f_{Ω} as the frequency), and ϕ is the phase to modulate the initial value of the swept voltage. This method has been fully described for the variables j_{d} and v_{s} in a recent paper.[8] Here we explore the additional effect due to the photocurrent-determining v_{b} variable. By integrating eq T4, we have the time dependence

$$v_{\mathrm{b}}(t) = V_{\mathrm{m}} + \frac{V_{\mathrm{p}}}{1+(\Omega\tau_{\mathrm{b}})^2}\left[\sin(\Omega t+\phi) - \Omega\tau_{\mathrm{b}}\cos(\Omega t+\phi) + \Omega\tau_{\mathrm{s}}e^{-t/\tau_{\mathrm{b}}}\right] \quad (7)$$

and this last expression is inserted into eq T1.

There is an intrinsic connection between the hysteresis effects obtained under large amplitude current-voltage cycling, and the small signal impedance features, as explained in the Appendix. Hysteresis is a time domain characteristic, and it can be probed by current-voltage cycling[9,10] and the corresponding transient current/voltage measurements. In many cases hysteresis and time transient patterns can be classified by the characteristic time constants of impedance spectroscopy and their associated conductances. We have shown in recent work the connection between time constants, conductance and transients[3,7] and their application to analyze the performance of solar cells.[2,4] Here we show some examples to illustrate the application of the methods, that can be complemented with the additional source of inductive hysteresis shown in Fig. 1 of the main text.

Fig. SI3 shows the change of time constants under degradation. In this model there are only two times constants, one capacitive and one inductive. Degradation enhances the inductive component, decreasing the open-circuit voltage.[11] In most cases the capacitive and inductive time constants coincide.[12,13] However the two time constant become different for the most degraded condition.

Fig. SI4 shows the classification for three time constants.[5]

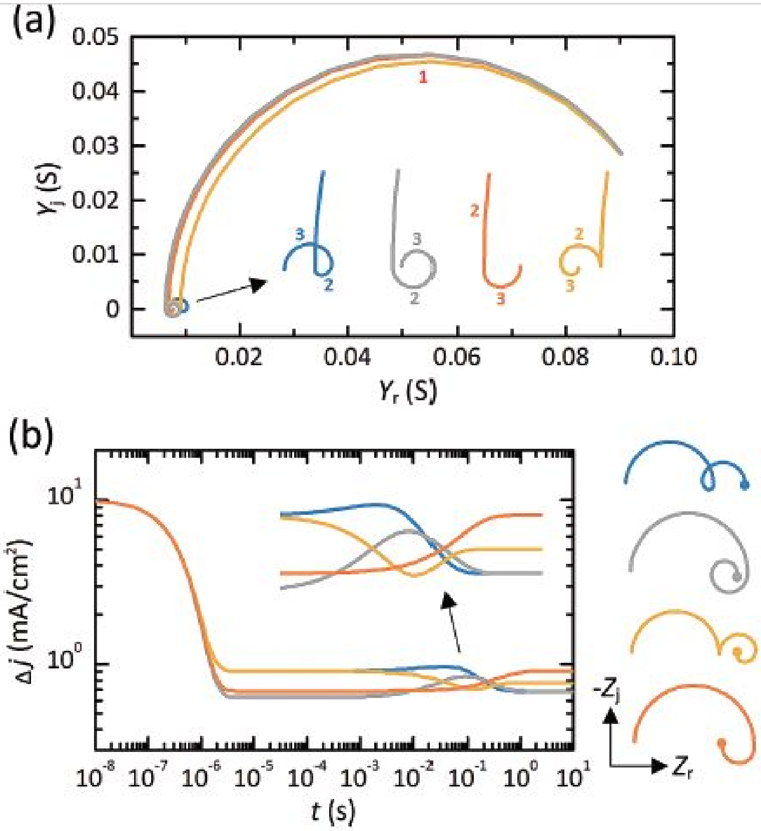

Figure 5. a) Numerical simulations of typical complex plane admittance plots in all types of perovskite semiconductors (e.g., solar cells, photodetectors, light-emitting diodes, and memristors) obtained for different kinetic relaxation constants and applied voltage biases by using the following values for the electrical elements of $R_s = 10\,\Omega$, $C_g = 50$ nF, $R_b = 300\,\Omega$ and the other ones $\{R_x, C_x, R_a, L_a\}$ as {150 Ω, 0.8 mF, 250 Ω, 10 H}, {300 Ω, 0.8 mF, 250 Ω, 10 H}, {250 Ω, 1 mF, 150 Ω, 50 H}, or {150 Ω, 0.4 mF, 200 Ω, 25 H} for the impedance patterns consisting of the inductive loop at intermediate frequencies, the spiral, the ideal chemical inductor structure, or the double capacitive arc that finalizes with inductive traces, respectively. Note that the processes have been numbered according to the order of appearance. b) Equivalent current transients in response to a voltage step of 10 mV exhibit a wide variety of dynamics with decays (capacitances), rises (inductors), and spike components. Next, we indicate the characteristic parameters obtained from Equations (11) and (12): $t_{spike,1} = 7.32\,\mu$s and $t_{spike,2} = 33.79$ ms for the inductive loop; $t_{spike} = 8.11\,\mu$s and $M_p = -24.65\%$ for the hook feature; $t_{spike,1} = 7.31\,\mu$s, $t_{spike,2} = 0.10$ s, and $M_p = -8.52\%$ for the inductive characteristic with a spiral trajectory; and $t_{spike} = 0.11$ s and $M_p = -15.38\%$ for the curl-back consisting of double capacitive arc that finalizes in a chemical inductor. Note that all these values provide excellent agreement with the simulation data. As a guide, impedance plots are also represented, where the dc conditions are marked by colored points.

Table 1. General model parameters of the time transient response depending on the inductive spectral pattern in the complex plane immittance representation.

Inductive feature	Code	τ_1	τ_2	τ_3	R_1	R_2	R_3	R_4
CLC: Loop or spiral		R_sC_g	$\frac{L_a}{R_a}$	R_xC_x	R_s	$R_s + \frac{R_bR_x}{R_b+R_x}$	$\frac{R_s + \left(\frac{1}{R_b} + \frac{1}{R_x} + \frac{1}{R_a}\right)^{-1}}{R_s + R_b}$	$R_s + \frac{R_aR_b}{R_a+R_b}$
CCL: Hook or curl-back			R_xC_x	$\frac{L_a}{R_a}$				

Fig. SI8. Classification of time transient responses for a model with three time constants, 2 capacitive and 1 inductive. Reproduced from Hernandez-Balaguera, E.; Bisquert, J., *Adv. Func. Mater.* **2023**, *34*, 2308678, licensed under a Creative Commons Attribution 4.0 International License (CC BY 4.0).[5]

S6. Interpretation of voltages and model assumptions

The model of Eqs. (T1-T5) is controlled by a single device voltage. The chemical inductor and variable capacitance terms generate the same type of circuit element: A series connection of a resistor and a reactive element (either inductor or capacitor), as explained

in the Appendix.

In our model we have made the definition that both voltage variables equilibrate to the same voltage: $v_s \to V$, $v_b \to V$. This does not mean that both internal voltages coincide with the whole external voltage V. There may be a constant relation, such that $v_s \to V - V_{s0}$, for instance. However, this will only introduce another constant parameter V_{s0} that we cannot measure, since it is absorbed by the prefactor of the capacitance. In the past, we have developed a fully consistent model for dc and ac polarization, based on the band diagram, as shown in the Fig. SI5.A.[13] This model contains the variable v_s both in the capacitance and in the slow recombination term of the current.

However, many devices contain a neat separation between the voltage in the bulk and the voltage in the contact, and this is also expressed in the diagrams of Fig. 2. Then one should distinguish a parallel mode (with the individual branches all controlled by V) or a series model, where a partition $V = V_1 + V_2$ applies to different subcircuits, or a combination of both. We have devoted discussions to this question.[14] In Fig. S7 we show a range of models that distinguish the bulk and surface effects, with the presence of inductors and capacitors in different places:

A. Impedance model from a surface polarization model. Here all the branches are in parallel.[13]

B. Model and impedance spectra of a MAPbBr perovskite solar cell.[12] The model distinguishes bulk (R_3) and surface elements, where the inductor is for surface recombination.

C. Model and impedance spectra of $FA_{0.85}$ $MA_{0.15}$ Pb(I 0.85 $Br_{0.15})_3)$ perovskite solar cell.[15] The model distinguishes bulk and surface elements, where the inductor is in the bulk component.

D. Model and measurement of a carbon-based perovskite solar cell.[16] The model distinguishes bulk (R_3) and surface components. The surface capacitor and inductor are described by **A**. An additional RC branch is added to account for the three observed capacitance plateaus.

A

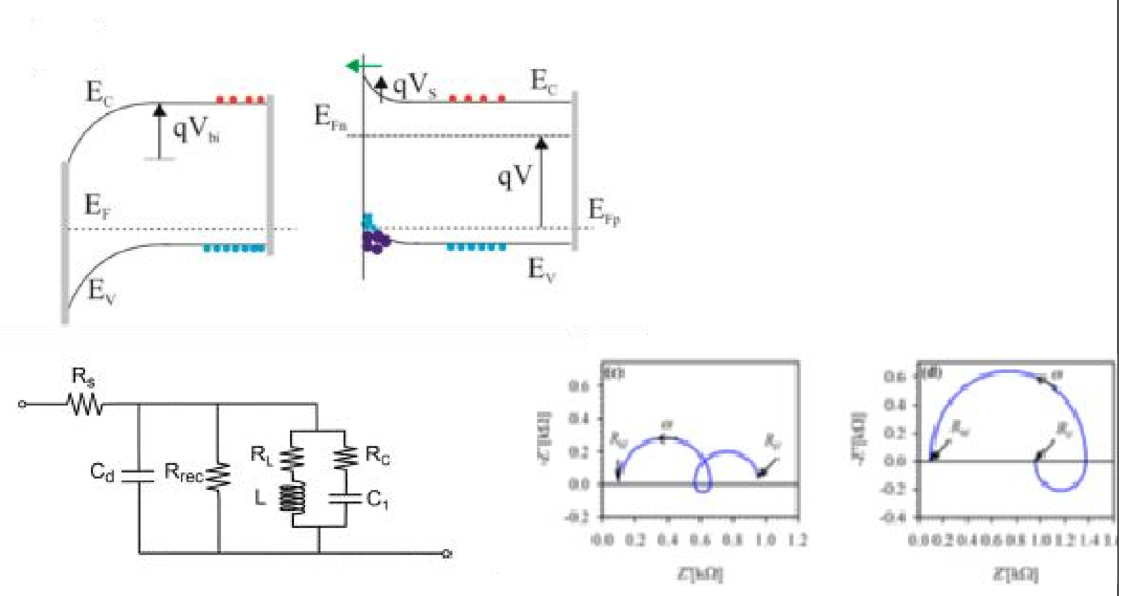
E
qV
E
E
qV
E
E
E
E
E
R
C
R
R
C
R
L
C
Z'[kΩ]
Z'[kΩ]

B

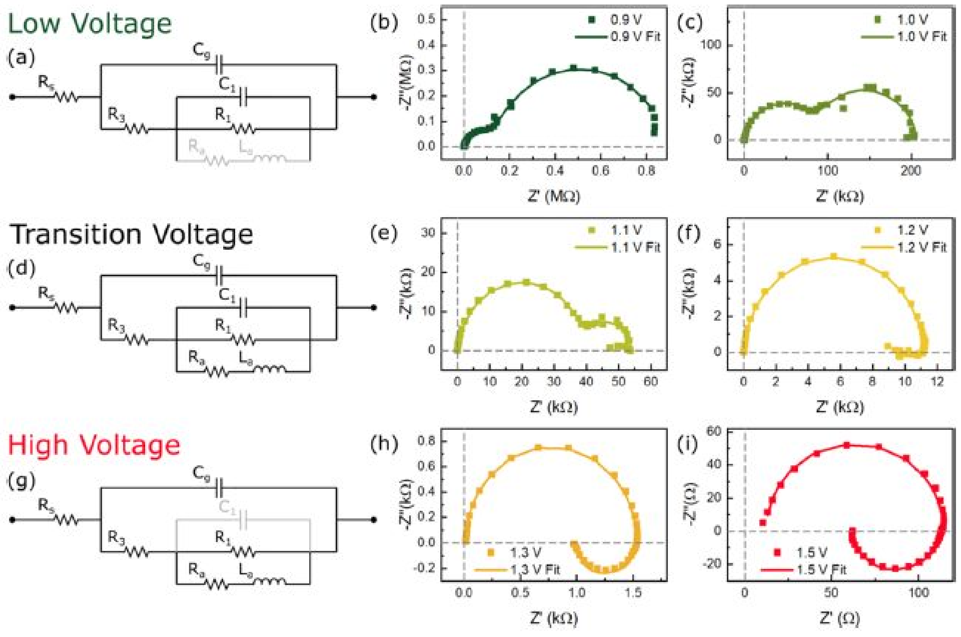
Low Voltage
(a)
(b)
(c)
0.9 V
0.9 V Fit
1.0 V
1.0 V Fit
-Z"(MΩ)
Z' (MΩ)
-Z"(kΩ)
Z' (kΩ)
Transition Voltage
(d)
(e)
(f)
1.1 V
1.1 V Fit
1.2 V
1.2 V Fit
High Voltage
(g)
(h)
(i)
1.3 V
1.3 V Fit
1.5 V
1.5 V Fit
-Z"(Ω)
Z' (Ω)

C

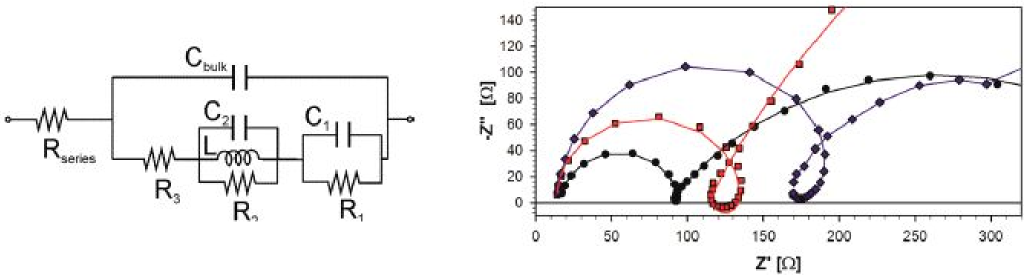

D

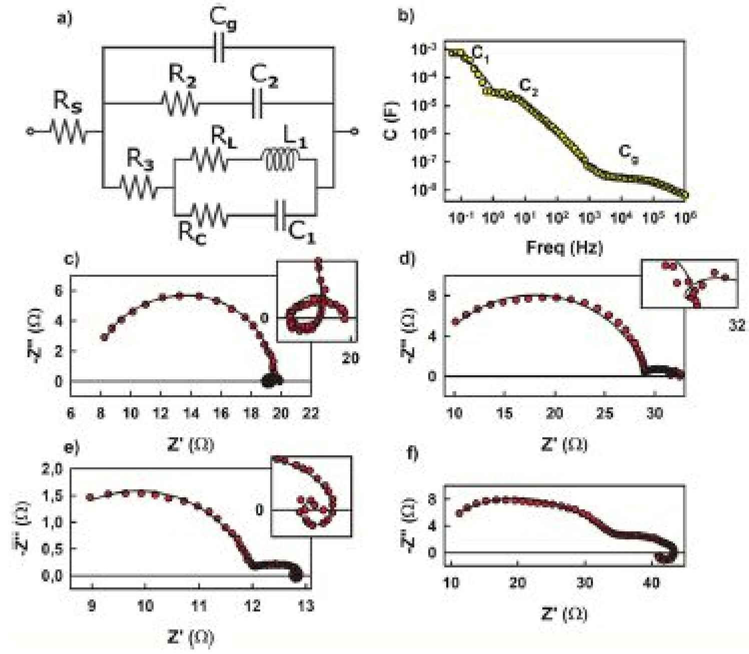

Fig. SI9. **A**. Impedance model from a surface polarization model. Reproduced with permission from Ghahremanirad, E.; Bou, A.; Olyaee, S.; Bisquert, J., *J. Phys. Chem. Lett.* **2017**, *8*, 1402–1406, copyright 2017, American Chemical Society.[13] **B.** Model and impedance spectra of a MAPbBr perovskite solar cell. Reproduced from Gonzales, C.; Guerrero, A.; Bisquert, J., *J. Phys. Chem. C* **2022**, *126*, 13560–13578, licensed under a Creative Commons Attribution 4.0 International License (CC BY 4.0)[12] **C.** Model and impedance spectra of $FA_{0.85}$ $MA_{0.15}$ Pb(I 0.85 $Br_{0.15})_3$) perovskite solar cell. Reproduced from Guerrero, A.; Garcia-Belmonte, G.; Mora-Sero, I.; Bisquert, J. et al., *J. Phys. Chem. C* **2016**, *120*, 8023–8032, licensed under a Creative Commons Attribution 4.0 International License (CC BY 4.0)[15] D. Model and measurement of a carbon-based perovskite solar cell. Reproduced from Bou, A.; Bisquert, J.; et al., *J. Phys. Chem. Lett.* **2020**, *11*, 8654–8659, licensed under a Creative Commons Attribution 4.0 International

License (CC BY 4.0)[16]

The solar cells in Fig. SI9 produce varied behaviours, which is very natural considering the enormous diversity of composition, materials, and morphology, that can be obtained with halide perovskite solar cells. At present we find it more practical for the analysis of impedance spectroscopy results to use a simpler expression of the differential equations that goes straight to the impedance parameters. This first order approximation to the problem can be completed with more detailed approaches if the situation allows more work or merits further investigation. In the present paper, we have provided an interpretation of the bulk inductor added to the surface recombination components using a single voltage model. In a specific experimental investigation, the impedance can be analyzed using different combinations as shown in Fig. SI9.

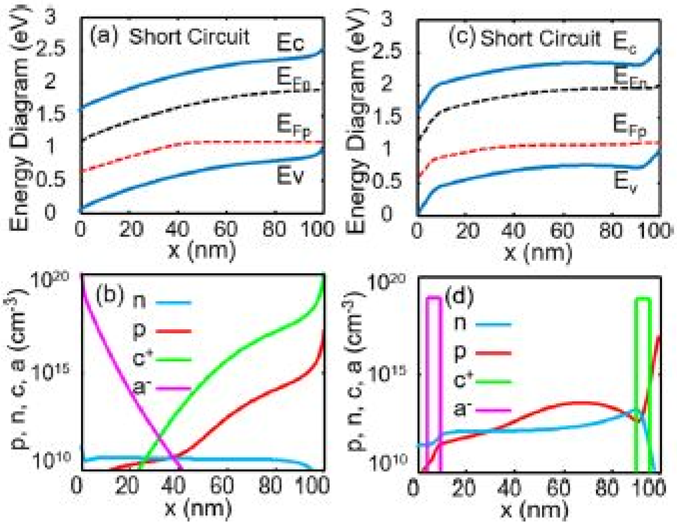

Fig. SI10. Energy band diagram and distributions of ions, electrons, and holes for a p-doped perovskite solar cell (with electron and hole selective contacts in the left and right side, respectively) under illumination in short-circuit steady state, (a, b) including drift-diffusion of ions in the numerical simulation and (c, d) assuming a fixed dipolar-distribution of ions in the numerical simulation. Reproduced with permission from Lopez-Varo, P.; Bisquert, J.; et al., *ACS Energy Lett.* **2017**, *2*, 1450–1453, copyright 2017, American Chemical Society.[17]

S7. Discussion on numerical methods

There is also another effect affecting the voltage distribution, associated to the complex mixed ionic-electronic nature of the halide perovskites. Consider the surface barrier in Fig. SI8A. It is not clear if this barrier is at the contact or penetrates the bulk region. Fig. SI10 shows the complex charge-compensation distribution that may appear (case b) instead of simple ionic-electronic charge accumulation at the interfacial Helmholtz layers

(case d, corresponding to Fig. 2). A wide variety of situations according to the ionic charge balance may occur.[18] Capacitive methods enable the study of the interface charging.[19,20] But even the simplest Mott-Schottky method to determine ion concentration and flatband potential is deeply problematic in perovskite solar cells.[21,22] In general, if there is a considerable number of injected charges, the traditional vision of a surface depletion region (or dipole layer) determining the surface capacitance collapses.[23] One finds a catalogue of complex transients and transport effects involving the ion and electron transport coupling, due to the mobility of the "doping" ions and electrical field screening.[20,24-26] Then it is not easy to define a priori a bulk and a contact potential.

Since many years the separation of bulk and surface impedance (the latter mainly determined by effects of the transport layers) has been studied through modifications of contact layers, device thickness, and light soaking conditions.[1,15,27-29] Recently, new methodologies have been developed by Ravishankar et al. to establish the influence of charge transport layers.[30,31] These works show limitations of impedance to detect different intrinsic time constants, so that transient decay analysis needs to be combined with the frequency methods.

The models based on a simple set of Eqs. like (T1-T5), only recognize a spatial region with two contacts and one voltage. One can form series models, with two distinct regions, associated to a distinction of bulk and contact impedances, as we have shown in Fig. SI7. However, the reality is that mixed ionic-electronic transport and charge compensation creates a huge variety of situations, as indicated in Fig. SI10.

The questions about ion distributions and their effect on performance are much better answered using a model that considers all the distinct points in the device, using transport equations for all the carriers, the Poisson equation, and charge transfer conditions to the transport layers.[30,32] Recently, there is progress in impedance models that are fully spatially resolved.[29,33-36] This progress is very useful, with the advantage of these methods being that they may provide information of what effects are caused in the impedance (and also hysteresis, transients, etc.) even in the complicated combinations of carriers and layer distributions, recombination, etc. We have used the drift-diffusion approach in Se. 4 above to understand the effect of relaxing/frozen Helmholtz layers. Another example is the analysis of large photoinduced capacitances.[37]

The development of simulation models has the mentioned advantages, but also – in the present form – some important limitations. In our view, the aim of the analysis of impedance spectroscopy is not just "explaining" spectra, but tracking the evolution of internal parameters over changing external parameters like current, light, voltage, gas influx, and device composition or contact layers. Large amount of the data must be fitted and the variation of parameters needs to be understood by materials and geometry modifications, that sometimes produce changes that one does not expect, due to complex internal interactions. So far, the numerical methods are often not used in this way, and they mainly "explain" results of impedance spectra. Maybe these methods can be enhanced in the future into high throughput real data treatment, since techniques are being

developed to extract parameters from experimental data.[38] However, the mainstream of impedance spectroscopy in all kinds of research fields uses equivalent circuits for such data treatment, with software that is adapted to the measuring equipment like Zview. The last generation software approaches https://rhd-instruments.de/solutions-and-products/for-eis-data-analysis/relaxis/ continue adopting equivalent circuits as the main tool, and the AI-assisted methods go in the direction of self-fitting.

A practical problem associated with drift-diffusion methods is the multiplicity of solutions. Impedance spectroscopy contains very limited information with respect to all the internal details of orthodox semiconductor modelling, since in the end only a ratio $\tilde{V}/\tilde{j}$ is calculated. Thus, there are a great range of assumptions leading to the same impedance spectra. This is not an inherent limitation of the system itself, but rather a consequence of the measurement technique. It should further serve as a caution that one should not rely solely on a specific model as the primary explanation.

For example, Refs [34,39] explain that "the switching between electron-limited and hole-limited bulk recombination causes a change in the sign of the LF feature when the cell is otherwise unchanged. This is based on the Equation[39]

$$C_{\mathrm{LF}} = \frac{n_{\mathrm{ap}}(V_{\mathrm{dc}})j_{\mathrm{rec}}(V_{\mathrm{dc}})}{G_{+}V_T n_{\mathrm{el}}\left(n_{\mathrm{ap}}(V_{\mathrm{dc}})-n_{\mathrm{el}}\right)}\left(-\frac{dQ_{\mathrm{dc}}}{dV_{\mathrm{dc}}}\right).$$

in which the low frequency capacitance can become negative.

As discussed in the main text, strictly negative capacitances have not been observed to our knowledge. The feature observed in perovskite solar cells and denoted in the popular jargon "negative capacitance" is a positive inductor, not a negative capacitor.

The inductor is explained[34,39] by "changing the bulk SRH recombination from electron-limited to hole-limited." This is one mechanism, which is enabled by drift-diffusion equations. In another explanation "the transport layer permittivities have been swapped to be consistent with an inorganic ETL and an organic HTL".[34]

Yet in another paper the authors explain inverted hysteresis (which should generate an inductor) with a charge imbalance related to ion charge, generating a substantial electrical field.[36] This is obtained by the equations

$$D_P N_0 \frac{\partial^2\phi}{\partial x^2} = \frac{d_H g_v}{g_v^H}\exp\left(\frac{V(t)-V_{bi}-2\phi}{2V_T}+\frac{E_v-E_v^H}{qV_T}\right)\frac{\partial}{\partial t}\left(\phi-\frac{V(t)}{2}\right)-\frac{\varepsilon_P V_T}{q}\frac{\partial}{\partial t}\left(\frac{\partial^2\phi}{\partial x^2} \tag{27a}$$

which couples to the perovskite boundary layer charge densities via the ODEs

$$\frac{dQ_L}{dt} = \frac{qD_P N_0}{V_T}\frac{\partial\phi}{\partial x}\Big|_{x=0^+}, \tag{27b}$$

$$\frac{dQ_R}{dt} = -\frac{qD_P N_0}{V_T}\frac{\partial\phi}{\partial x}\Big|_{x=b^-}, \tag{27c}$$

that generates a slow response of the internal electrical field. Whether these mechanisms obtained in the equations correspond to what happens in reality has not been verified in

real case experiments, for example "swapping the transport layer permittivities".

Obtaining different models that generate the same impedance response are positive developments; however, one may ask: What do these models have in common? Is there a unifying mechanism behind them? This is answered by the elementary model of the chemical inductor, that establishes fundamental conditions to obtain the inductive response in impedance and hysteresis. However, the model only provides two parameters, a resistance and a relaxation time. Then there is an important issue of obtaining an interpretation of such parameters in terms of specific physio-chemical properties of the device. This is when microscopic-based transport/reaction models become very important tools. We highlight the work by Anta and coworkers that combines full analysis of the experimental data with equivalent circuits under changing materials conditions, and the spatially-resolved simulation for understanding the parameter evolution.[29,40,41]

Another limitation is that the drift-diffusion methods tend to explain spectral shapes with the orthodox semiconductor modelling equation, based on charge compensation, transport etc. However, halide perovskites are unconventional materials from this point of view that contain other factors such as electrochemical reactions, that are hard to model from the orthodox approach, and are not described in those methods, as far as we know. These effects are very important for degradation studies. Organic layers may lose performance, contact ions may leak into the perovskite layer.[42-45] Reactivity conditions can be identified in the low frequency region of the capacitance plots.[42]

In summary, the model (T1-T5) is a zero-order model for the halide perovskite solar cells that can be completed in several respects.

(a) Correlation of techniques. Impedance sometimes cannot show relevant information that the system contains. In some cases transport features are hidden,[46,47] and in other cases nonlinear response becomes large and relevant for application.[48] Different small signal modulated techniques can be combined,[46,49] and time domain small or large perturbation methods provide essential information.[2-5,10,50]

(b) Transport layers and charge compensation. Among the many different aspects of the impedance of perovskite solar cells, we find special complexity in the analysis of transport layers and large charge densities close to them, as commented above. Here the neuron-based models give a first guidance of the observations, but other methods are needed.

(b1) Experimental methods can be developed that probe specifically interfacial ionic-electronic charging[19,20,49,51,52] and the effective time constants of the transport layers. [30,31]

(b2) Under strong inhomogeneous conditions the previous methods need to be complemented with detailed spatially resolved modelling techniques, based on drift-diffusion approaches.

(b3) Electrochemical and photochemical reactions and complex defect chemistry.[29,33-36,40,41,53,54] form a substantial part of ionic-electronic dynamics in

halide perovskites.[54] These phenomena need to be considered in the interpretation of experiments.[55,56]

SI References

(1) Correa-Baena, J.-P.; Turren-Cruz, S.-H.; Tress, W.; Hagfeldt, A.; Aranda, C.; Shooshtari, L.; Bisquert, J.; Guerrero, A. Changes from Bulk to Surface Recombination Mechanisms between Pristine and Cycled Perovskite Solar Cells, *ACS Energy Lett.* **2017**, 681–688.

(2) Balaguera, E. H.; Bisquert, J. Evolution of Performance Parameters of Perovskite Solar Cells with Current–Voltage Scan Frequency, *Energy & Fuels* **2025**, *39*, 3638–3648.

(3) H. Balaguera, E.; Bisquert, J. Mapping of Internal Ionic/Electronic Transient Dynamics in Current–Voltage Operation of Perovskite Solar Cells, *Small* **2024**, *21*, 2409534.

(4) Balaguera, E. H.; Bisquert, J. Accelerating the Assessment of Hysteresis in Perovskite Solar Cells, *ACS Energy Lett.* **2024**, *9*, 478–486.

(5) Hernandez-Balaguera, E.; Bisquert, J. Time Transients with Inductive Loop Traces in Metal Halide Perovskites, *Adv. Func. Mater.* **2023**, *34*, 2308678.

(6) Hernández-Balaguera, E.; Bisquert, J. Negative Transient Spikes in Halide Perovskites, *ACS Energy Lett.* **2022**, 2602–2610.

(7) Bisquert, J. Hysteresis, Impedance, and Transients Effects in Halide Perovskite Solar Cells and Memory Devices Analysis by Neuron-Style Models, *Adv. Energy Mater.* **2024**, *n/a*, 2400442.

(8) Balaguera, E. H.; Bisquert, J. Evolution of Performance Parameters of Perovskite Solar Cells with Current–Voltage Scan Frequency, *Energy & Fuels* **February 10, 2025**, *39*.

(9) Pellet, N.; Giordano, F.; Ibrahim Dar, M.; Gregori, G.; Zakeeruddin, S. M.; Maier, J.; Grätzel, M. Hill climbing hysteresis of perovskite-based solar cells: a maximum power point tracking investigation, *Prog. Photov.: Res. Appl.* **2017**, *25*, 942–950.

(10) Le Corre, V. M.; Diekmann, J.; Peña-Camargo, F.; Thiesbrummel, J.; Tokmoldin, N.; Gutierrez-Partida, E.; Peters, K. P.; Perdigón-Toro, L.; Futscher, M. H.; Lang, F.; Warby, J.; Snaith, H. J.; Neher, D.; Stolterfoht, M. Quantification of Efficiency Losses Due to Mobile Ions in Perovskite Solar Cells via Fast Hysteresis Measurements, *Solar RRL* **2022**, *6*, 2100772.

(11) Fabregat-Santiago, F.; Kulbak, M.; Zohar, A.; Vallés-Pelarda, M.; Hodes, G.; Cahen, D.; Mora-Seró, I. Deleterious Effect of Negative Capacitance on the Performance of Halide Perovskite Solar Cells, *ACS Energy Lett.* **2017**, *2*, 2007–2013.

(12) Gonzales, C.; Guerrero, A.; Bisquert, J. Transition from capacitive to inductive hysteresis: A neuron-style model to correlate I-V curves to impedances of metal

halide perovskites, *J. Phys. Chem. C* **2022**, *126*, 13560–13578.

(13) Ghahremanirad, E.; Bou, A.; Olyaee, S.; Bisquert, J. Inductive Loop in the Impedance Response of Perovskite Solar Cells Explained by Surface Polarization Model, *J. Phys. Chem. Lett.* **2017**, *8*, 1402–1406.

(14) Guerrero, A.; Bisquert, J.; Garcia-Belmonte, G. Impedance spectroscopy of metal halide perovskite solar cells from the perspective of equivalent circuits, *Chemical Reviews* **2021**, *121*, 14430–14484.

(15) Guerrero, A.; Garcia-Belmonte, G.; Mora-Sero, I.; Bisquert, J.; Kang, Y. S.; Jacobsson, T. J.; Correa-Baena, J.-P.; Hagfeldt, A. Properties of Contact and Bulk Impedances in Hybrid Lead Halide Perovskite Solar Cells Including Inductive Loop Elements, *J. Phys. Chem. C* **2016**, *120*, 8023–8032.

(16) Bou, A.; Pockett, A.; Raptis, D.; Watson, T.; Carnie, M. J.; Bisquert, J. Beyond Impedance Spectroscopy of Perovskite Solar Cells: Insights from the Spectral Correlation of the Electrooptical Frequency Techniques, *J. Phys. Chem. Lett.* **2020**, *11*, 8654–8659.

(17) Lopez-Varo, P.; Jiménez-Tejada, J. A.; García-Rosell, M.; Anta, J. A.; Ravishankar, S.; Bou, A.; Bisquert, J. Effects of Ion Distributions on Charge Collection in Perovskite Solar Cells, *ACS Energy Lett.* **2017**, *2*, 1450–1453.

(18) Sivadas, D.; Singareddy, A.; Vinod, C. G.; Nair, P. R. Ionic Charge Imbalance in Perovskite Solar Cells, *J. Phys. Chem. C* **2023**, *127*, 22766–22774.

(19) Almora, O.; Guerrero, A.; Garcia-Belmonte, G. Ionic charging by local imbalance at interfaces in hybrid lead halide perovskites, *Appl. Phys. Lett.* **2016**, *108*, 043903.

(20) Diethelm, M.; Lukas, T.; Smith, J.; Dasgupta, A.; Caprioglio, P.; Futscher, M.; Hany, R.; Snaith, H. J. Probing ionic conductivity and electric field screening in perovskite solar cells: a novel exploration through ion drift currents, *Energy Environ. Sci.* **2025**, *18*, 1385–1397.

(21) Almora, O.; Aranda, C.; Garcia-Belmonte, G. Do Capacitance Measurements Reveal Light-Induced Bulk Dielectric Changes in Photovoltaic Perovskites?, *J. Phys. Chem. C* **2018**, *122*, 13450–13454.

(22) Almora, O.; Aranda, C.; Mas-Marzá, E.; Garcia-Belmonte, G. On Mott-Schottky analysis interpretation of capacitance measurements in organometal perovskite solar cells, *Appl. Phys. Lett.* **2016**, *109*, 173903.

(23) Ravishankar, S.; Bisquert, J.; Kirchartz, T. Interpretation of Mott–Schottky plots of photoanodes for water splitting, *Chemical Science* **2022**, *13*, 4828–4837.

(24) García-Batlle, M.; Mayén Guillén, J.; Chapran, M.; Baussens, O.; Zaccaro, J.; Verilhac, J.-M.; Gros-Daillon, E.; Guerrero, A.; Almora, O.; Garcia-Belmonte, G.

Coupling between Ion Drift and Kinetics of Electronic Current Transients in MAPbBr3 Single Crystals, *ACS Energy Lett.* **2022**, *7*, 946–951.

(25) Almora, O.; Matt, G. J.; These, A.; Kanak, A.; Levchuk, I.; Shrestha, S.; Osvet, A.; Brabec, C. J.; Garcia-Belmonte, G. Surface versus Bulk Currents and Ionic Space-Charge Effects in CsPbBr3 Single Crystals, *J. Phys. Chem. Lett.* **2022**, 3824–3830.

(26) Almora, O.; Lopez-Varo, P.; Cho, K. T.; Aghazada, S.; Meng, W.; Hou, Y.; Echeverría-Arrondo, C.; Zimmermann, I.; Matt, G. J.; Jiménez-Tejada, J. A.; Brabec, C. J.; Nazeeruddin, M. K.; Garcia-Belmonte, G. Ionic dipolar switching hinders charge collection in perovskite solar cells with normal and inverted hysteresis, *Sol. En. Mater. Sol. Cell* **2019**, *195*, 291–298.

(27) Zarazua, I.; Han, G.; Boix, P. P.; Mhaisalkar, S.; Fabregat-Santiago, F.; Mora-Seró, I.; Bisquert, J.; Garcia-Belmonte, G. Surface Recombination and Collection Efficiency in Perovskite Solar Cells from Impedance Analysis, *J. Phys. Chem. Lett.* **2016**, *7*, 5105–5113.

(28) Yoo, S.-M.; Yoon, S. J.; Anta, J. A.; Lee, H. J.; Boix, P. P.; Mora-Seró, I. An Equivalent Circuit for Perovskite Solar Cell Bridging Sensitized to Thin Film Architectures, *Joule* **2019**, *3*, 2535–2549.

(29) Riquelme, A. J.; Valadez-Villalobos, K.; Boix, P. P.; Oskam, G.; Mora-Seró, I.; Anta, J. A. Understanding equivalent circuits in perovskite solar cells. Insights from drift-diffusion simulation, *Phys. Chem. Chem. Phys.* **2022**, *24*, 15657–15671.

(30) Ravishankar, S.; Liu, Z.; Wang, Y.; Kirchartz, T.; Rau, U. How Charge Carrier Exchange between Absorber and Contact Influences Time Constants in the Frequency Domain Response of Perovskite Solar Cells, *PRX Energy* **2023**, *2*, 033006.

(31) Ravishankar, S.; Kruppa, L.; Jenatsch, S.; Yan, G.; Wang, Y. Discerning rise time constants to quantify charge carrier extraction in perovskite solar cells, *Energy Environ. Sci.* **2024**, *17*, 1229–1243.

(32) Nandal, V.; Nair, P. R. Predictive Modeling of Ion Migration Induced Degradation in Perovskite Solar Cells, *ACS Nano* **2017**, *11*, 11505–11512.

(33) Neukom, M. T.; Schiller, A.; Züfle, S.; Knapp, E.; Ávila, J.; Pérez-del-Rey, D.; Dreessen, C.; Zanoni, K. P. S.; Sessolo, M.; Bolink, H. J.; Ruhstaller, B. Consistent Device Simulation Model Describing Perovskite Solar Cells in Steady-State, Transient, and Frequency Domain, *ACS Appl. Mat. Int.* **2019**, *11*, 23320–23328.

(34) Clarke, W.; Richardson, G.; Cameron, P. Understanding the Full Zoo of Perovskite Solar Cell Impedance Spectra with the Standard Drift-Diffusion Model, *Adv. Energy Mater.* **2024**, *14*, 2400955.

(35) Clarke, W.; Cameron, P.; Richardson, G. Predicting Long-Term Stability from Short-Term Measurement: Insights from Modeling Degradation in Perovskite Solar

Cells during Voltage Scans and Impedance Spectroscopy, *J. Phys. Chem. Lett.* **2024**, *15*, 11730–11736.

(36) Clarke, W.; Cowley, M. V.; Wolf, M. J.; Cameron, P.; Walker, A.; Richardson, G. Inverted hysteresis as a diagnostic tool for perovskite solar cells: Insights from the drift-diffusion model, *J. Appl. Phys.* **2023**, *133*, 095001.

(37) Schiller, A.; Jenatsch, S.; Blülle, B.; Torre Cachafeiro, M. A.; Ebadi, F.; Kabir, N.; Othman, M.; Wolff, C. M.; Hessler-Wyser, A.; Ballif, C.; Tress, W.; Ruhstaller, B. Assessing the Influence of Illumination on Ion Conductivity in Perovskite Solar Cells, *J. Phys. Chem. Lett.* **2024**, *15*, 11252–11258.

(38) Zhan, H.; Ahmad, V.; Mayon, A.; Dansoa Tabi, G.; Bui, A. D.; Li, Z.; Walter, D.; Nguyen, H.; Weber, K.; White, T.; Catchpole, K. Physics-based extraction of material parameters from perovskite experiments via Bayesian optimization, *Energy Environ. Sci.* **2024**, *17*, 4735–4745.

(39) Bennett, L. J.; Riquelme, A. J.; Anta, J. A.; Courtier, N. E.; Richardson, G. Avoiding Ionic Interference in Computing the Ideality Factor for Perovskite Solar Cells and an Analytical Theory of Their Impedance-Spectroscopy Response, *Phys. Rev. Appl.* **2023**, *19*, 014061.

(40) Riquelme, A.; Bennett, L. J.; Courtier, N. E.; Wolf, M. J.; Contreras-Bernal, L.; Walker, A. B.; Richardson, G.; Anta, J. A. Identification of recombination losses and charge collection efficiency in a perovskite solar cell by comparing impedance response to a drift-diffusion model, *Nanoscale* **2020**, *12*, 17385–17398.

(41) Almora, O.; López-Varo, P.; Escalante, R.; Mohanraj, J.; Marsal, L. F.; Olthof, S.; Anta, J. A. Instability analysis of perovskite solar cells via short-circuit impedance spectroscopy: A case study on NiOx passivation, *J. Appl. Phys.* **2024**, *136*, 094502.

(42) Aranda, C.; Bisquert, J.; Guerrero, A. Impedance spectroscopy of perovskite/contact interface: Beneficial chemical reactivity effect, *J. Chem. Phys.* **2019**, *151*, 124201.

(43) Zohar, A.; Kedem, N.; Levine, I.; Zohar, D.; Vilan, A.; Ehre, D.; Hodes, G.; Cahen, D. Impedance Spectroscopic Indication for Solid State Electrochemical Reaction in (CH3NH3)PbI3 Films, *J. Phys. Chem. Lett.* **2016**, *7*, 191–197.

(44) Guerrero, A.; You, J.; Aranda, C.; Kang, Y. S.; Garcia-Belmonte, G.; Zhou, H.; Bisquert, J.; Yang, Y. Interfacial degradation of planar lead halide perovskite solar cells, *ACS Nano* **2016**, *10*, 218–224.

(45) Domanski, K.; Correa-Baena, J.-P.; Mine, N.; Nazeeruddin, M. K.; Abate, A.; Saliba, M.; Tress, W.; Hagfeldt, A.; Grätzel, M. Not All That Glitters Is Gold: Metal-Migration-Induced Degradation in Perovskite Solar Cells, *ACS Nano* **2016**, *10*, 6306–

6314.

(46) Bou, A.; Pockett, A.; Cruanyes, H.; Raptis, D.; Watson, T.; Carnie, M. J.; Bisquert, J. Limited information of impedance spectroscopy about electronic diffusion transport: The case of perovskite solar cells, *APL Materials* **2022**, *10*, 051104.

(47) Ravishankar, S.; Aranda, C.; Sanchez, S.; Bisquert, J.; Saliba, M.; Garcia-Belmonte, G. Perovskite Solar Cell Modeling Using Light and Voltage Modulated Techniques, *J. Phys. Chem. C* **2019**, *123*, 6444–6449.

(48) Lopez-Richard, V.; Pradhan, S.; Wengenroth Silva, R. S.; Lipan, O.; Castelano, L. K.; Höfling, S.; Hartmann, F. Beyond equivalent circuit representations in nonlinear systems with inherent memory, *J. Appl. Phys.* **2024**, *136*, 165103.

(49) Gillespie, S. C.; Alvarez, A. O.; Thiesbrummel, J.; Gevaerts, V. S.; Geerligs, L. J.; Ehrler, B.; Coletti, G.; Garnett, E. C. Intensity-Modulated Photoluminescence Spectroscopy for Revealing Ionic Processes in Halide Perovskites, *ACS Energy Lett.* **2025**, *10*, 3122–3131.

(50) Thiesbrummel, J.; Shah, S.; Gutierrez-Partida, E.; Zu, F.; Peña-Camargo, F.; Zeiske, S.; Diekmann, J.; Ye, F.; Peters, K. P.; Brinkmann, K. O.; Caprioglio, P.; Dasgupta, A.; Seo, S.; Adeleye, F. A.; Warby, J.; Jeangros, Q.; Lang, F.; Zhang, S.; Albrecht, S.; Riedl, T.; Armin, A.; Neher, D.; Koch, N.; Wu, Y.; Le Corre, V. M.; Snaith, H.; Stolterfoht, M. Ion-induced field screening as a dominant factor in perovskite solar cell operational stability, *Nat. Energy* **2024**, *9*, 664–676.

(51) Schmidt, M. C.; Ehrler, B. How Many Mobile Ions Can Electrical Measurements Detect in Perovskite Solar Cells?, *ACS Energy Lett.* **2025**, *10*, 2457–2460.

(52) Schmidt, M. C.; Alvarez, A. O.; de Boer, J. J.; van de Ven, L. J. M.; Ehrler, B. Consistent Interpretation of Time- and Frequency-Domain Traces of Ion Migration in Perovskite Semiconductors, *ACS Energy Lett.* **2024**, 5850–5858.

(53) Meggiolaro, D.; Mosconi, E.; De Angelis, F. Modeling the Interaction of Molecular Iodine with MAPbI3: A Probe of Lead-Halide Perovskites Defect Chemistry, *ACS Energy Lett.* **2018**, *3*, 447–451.

(54) Meggiolaro, D.; De Angelis, F. First-Principles Modeling of Defects in Lead Halide Perovskites: Best Practices and Open Issues, *ACS Energy Lett.* **2018**, *3*, 2206–2222.

(55) Kim, G. Y.; Senocrate, A.; Wang, Y.-R.; Moia, D.; Maier, J. Photo-Effect on Ion Transport in Mixed Cation and Halide Perovskites and Implications for Photo-Demixing, *Angew. Chem. Int. Ed.* **2021**, *60*, 820–826.

(56) Senocrate, A.; Moudrakovski, I.; Kim, G. Y.; Yang, T.-Y.; Gregori, G.; Grätzel, M.; Maier, J. The Nature of Ion Conduction in Methylammonium Lead Iodide: A Multimethod Approach, *Angew. Chem. Int. Ed.* **2017**, *56*, 7755–7759.
