# Supplementary material for: Dynamic Screening and the Chemical Inductor of Perovskite Solar Cells: From J–V Transients to Impedance Spectroscopy
Source: J Phys Chem Lett. 2025 Oct 15;16(42):11053–63. doi: 10.1021/acs.jpclett.5c01916 (PMC12557368; doi:10.1021/acs.jpclett.5c01916)
Supplement: Supplementary file 2 [file jz5c01916_si_002.pdf]

jz-2025-01916q.R1

Name: Peer Review Information for "Impedance Spectroscopy of Perovskite Solar Cells: Linking Double Inductance to Ionic Dynamics"

First Round of Reviewer Comments

Reviewer: 1

Comments to the Author
In this manuscript, the authors present a model that incorporates the effects of collection efficiency and different types of recombination in perovskite solar cells. They simulate the device response under both current-voltage scans at varying frequencies and impedance spectroscopy measurements. The model successfully reproduces a wide range of observed features, particularly the double low-frequency inductive loops and intermediate inductive responses.

While the ability to reproduce these features is notable and constitutes a valuable contribution, several key aspects of the model require clarification and refinement before the manuscript can be considered for publication:

1. Internal Voltages (v_b and v_s):
The model introduces two internal voltages: v_b, associated with bulk ion field screening, and v_s, linked to ionic accumulation at the surface. Both voltages follow similar relaxation dynamics and relax toward the applied voltage. However, this raises significant concerns. From a phenomenological standpoint, it is unclear why two distinct internal voltages would independently relax to the same external value. If v_b and v_s both equal the applied voltage, their distinction becomes ambiguous. A schematic representation of the internal voltage distribution within the device is necessary to clarify how these voltages are defined and how they relate to each other and to the applied bias. Although for impedance analysis the specific relaxation value may not change the equivalent circuit, from a physical modeling perspective, this formulation needs better justification.

2. Surface Recombination vs. Surface Voltage:
The manuscript title emphasizes the combined effect of collection efficiency and surface recombination. However, the model implements two types of recombination: a fast and a slow component, with the slow one governed by an additional relaxation equation. The connection between this slow recombination process and the previously defined surface voltage v_s is not made clear. If v_s is intended to represent surface phenomena, how does it not couple directly to surface recombination? The model appears to treat v_s as purely capacitive, which contradicts the interpretation that it should modulate recombination processes. The authors should clarify the physical basis for associating the slow recombination term with surface effects and explicitly relate it to the dynamics of v_s, or revise the terminology if this association is not intended.

3. Model Partitioning – Collection vs. Recombination:
The model is partitioned into two separate components: one governing collection efficiencies due to diffusion and transport, and another describing recombination dynamics. While this separation may simplify impedance analysis, it potentially overlooks the fact that collection efficiency inherently depends on recombination processes, particularly through diffusion-limited transport. The manuscript should address how this decoupling is justified, and whether it leads to internal inconsistencies in the physical interpretation of the model.

4. Inductive Features and Illumination Conditions:
One of the inductive features attributed to the bulk voltage appears in the photocurrent term of the model. However, Figure 3a is reproduced from a reference in which the measurements were performed under dark conditions. This raises concerns because the corresponding inductive feature in the model depends on the photocurrent, which would not be present in the dark. Empirically, it is common to observe inductive features in perovskite devices without illumination. Furthermore, the references cited by the authors to support the existence of two inductive features are based on simulations rather than experimental data. While one cited work (https://doi.org/10.1021/acsenergylett.7b00542) does report a double inductive response under illumination, the model should be adapted to account for such features independently of the photocurrent, particularly if it aims to describe dark conditions accurately.

5. Novelty and Prior Work:
While the model presents novel aspects, the manuscript cites two prior works as experimental evidence when they are in fact theoretical studies that have already reproduced similar features. The authors should clearly delineate what is new in their approach compared to the models in https://doi.org/10.1002/aenm.202400955 and https://doi.org/10.1021/acs.jpclett.4c02343. This includes highlighting either improved physical insight, broader feature reproduction, or experimental validation.

Reviewer: 2

Comments to the Author

Report	Review paper JPCL
Title:	***Combined action of collection efficiency and surface recombination effects in halide perovskite solar cells by impedance spectroscopy model***
Date:	25 07 10

1. What is the major advance reported in the paper?

This manuscript provides a mathematical model to explain an unusual experimental observation: two inductor-like features at low frequencies in perovskite devices. The model proposes that these inductive characteristics are the result of two distinct physical processes occurring within the system: ion-mediated charge recombination and electric field screening by those same charges, claiming the utility of the findings as a tool to identify degradation processes in perovskite solar cells.

2. What is the immediate significance of this advance?

This referee recognizes the importance of clarifying the various impedance features observed in perovskite solar cells, especially those related to ionic motion, given their critical role in degradation mechanisms.

However, while the mathematical model presented is robust, there's a notable lack of experimental evidence to sustain the findings. Simulations are an excellent tool for predicting behaviors, but without experimental confirmation, the reliability of the claims is not high enough.

3. Technical suggestions

I suggest the authors confirm their findings with targeted experiments. Alternatively, they could review the existing literature to see how ionic effects in perovskite solar cells have been mitigated through additives or passivation strategies, and how this has manifested in distinct impedance responses.

Additionally, some highly pertinent studies relevant to this context are missing from the references. This reviewer believes their inclusion is essential.

Overall, this referee considers the proposed model interesting and promising, but related experimental evidence supporting the findings needs to be provided prior to publication.

Reviewer: 3

Comments to the Author
The electro-ionic response of the perovskite solar cell is analyzed by proposing a unified model of recombination, polarization, electric field screening and charge collection effects. The unified model allows to calculate the impedance response to explain the low-frequency double inductor feature, linked separately to ion driven recombination and electric field screening effects. Interestingly, by comparing with impedance spectroscopy experimental data, this compact model can provide insights into the physical/degradation mechanisms hindering device performance. Due to the importance of the work, I highly recommend the publication of this work in JPCL after major and minor modifications.

1. The definition of V0 is quite interesting. The manuscript refers to it both as “a constant related to the built-in voltage” and as “the effective built-in voltage under stationary operation.” Could the authors please clarify what is meant by "stationary condition" in this context? Specifically, when is V0 defined—under equilibrium, in the dark, and at zero applied bias?

2. Interpretation of Eq. (3): “Here, the internal voltage vb is the instantaneous bulk voltage that will equilibrate as vb→V in the long time, according to the equation.” Based on this formulation, it appears that the voltage drop across the transport layers (TLs) is considered negligible. Is this assumption correct?
If so, I would suggest making this assumption explicit in the main text. In particular, under conditions where the transport layers are lightly doped and the ionic concentration in the perovskite is high, the voltage drop across the TLs can be non-negligible.

3. In the Table 1, I was surprised by the value of the geometrical capacitance Cg = 10-2 F. Could the authors please provide more detail on how this value was determined?

4.In the conclusions, there is an interesting claim: “mobile ions in perovskite solar cells can lead to two primary effects affecting the stationary performance: an increase of recombination (lowering photovoltage) …”. I agree that, mobile ions screen the total electric field—comprising both internal and externally applied components—and can indeed lead to increased recombination and reduced photovoltage. However, this is not always the case. There are conditions, as when the TLs are lowly doped, in which ions can increase the quasi-fermi level splitting increasing the open circuit voltage. (for example: https://doi.org/10.1002/solr.202101087)

5. In Figure 1. Please, check the format of the variables; current density and voltage which should be in italic.

6. Figure 2.(b), there is also (a) inside the figure.

7. In the abstract you claim that : “We show that the ordering of characteristic capacitive and inductive relaxation times provides a convenient criterion to classify impedance spectra and hysteresis effects.” Where is in the text the classification between IS and hysteresis effect?

8. “In Figure 2c we show that removing one time, $\tau \rightarrow 0$, the corresponding spectral feature disappears while the DC resistance is the same, since the resistors occur equally, although they turn into a fast response, when the respective τ can be neglected.” What do you mean with “they turn into a fast response”?

9. In equation T11, Is the current Js the actual current Jd in T7?

10. At the end of the manuscript, there is an interesting analysis of the impact of the order of the different characteristic times. τg will be always smaller than τs. However, how can you distinguish the order between τd and τb? Both mechanisms, recombination and charge extraction, are slow. Or even, if you have only one inductive mechanism, how can you distinguish if it is coming from τd or τb?

Author's Response to Peer Review Comments:

Response letter
Manuscript ID: jz-2025-01916q

Reply to the reviewers

We sincerely thank the reviewers for their thoughtful evaluation of our manuscript entitled "Transient charging of mixed ionic-electronic conductors by anomalous diffusion." We are very grateful for the insightful comments and constructive suggestions, which have significantly improved the clarity, rigor, and overall quality of our work.

Below, we describe the main modifications of the manuscript, and provide a detailed, point-by-point response to each reviewer comment. Reviewer comments appear in standard font, our replies are shown in blue, and modifications to the manuscript are highlighted in yellow in the revised version.

We first highlight the main changes in the manuscript and Supporting Information

1. The paper has been largely rewritten to make a better presentation of the main points. We show a general demonstration of the capacitive and inductive hysteresis, and we show with physical diagrams the new aspect presented here, which the generation of inductive hysteresis by charge collection in the electrical field.

Hysteresis and light-induced negative capacitance effects have been amply observed and discussed in perovskite solar cells.[1,2] A majority of approaches coincide in attributing the primary cause to a sluggish ionic dynamic that in the end impacts the externally measured currents. The crucial method of analysis of perovskite solar cells by IS consists of tracking the evolution over a succession of steady states, and obtain the model parameters, and how they change under different external conditions. Most models have in common that internal state variables are defined, which describe the ionic delay and its impact on recombination and charge accumulation phenomena.[3-7] These methods have been applied by different groups.[8-11]

To summarize the connection of impedance and hysteresis in voltage cycling of the current, we assume the following equation for the current in the solar cell

$$j = J_{rec}(V) + \frac{\mathrm{d}Q_s(x)}{\mathrm{d}t} \tag{1}$$

Here $J_{rec}(V)$ is a recombination current that is a direct function of the external voltage V and $Q_s(x)$ is a charge function that depends on an internal variable x, such as a surface voltage v_s. This internal variable adapts to the changes of applied voltage V by the equation

$$\tau_s \frac{dx}{dt} = F(V) - x \tag{2}$$

where $F(V)$ is a nonlinear function and τ_s is a relaxation time. In the Appendix we show that the impedance due to the charge derivative in Eq. (1) is

$$Z = \frac{1}{g_C} + \frac{1}{C_s\, i\omega} \tag{3}$$

where $C_s = f\, dQ_s/dx$ is a capacitance, $f = dF/dV$, and $g_C = C_s/\tau_s$ is a conductance. Eq. (3) describes a series RC circuit.

If, however, the current has the expression

$$j = j_a(x) \tag{4}$$

for a nonlinear function j_a of the variable x, that is also controlled by Eq. (2), then the impedance

$$Z = \frac{1}{g_L} + L_b\, i\omega \tag{5}$$

is a series connection of a resistor and inductor, where $g_L = m\, f$ is a conductance, $m(x) = dj_a/dx$, and $L_b = \tau_s/m\, f$ is an inductance.

Note that the variable x in Eq. (2) can have a very many different interpretations according to the specific system. The Eq. (2) is first found on the Hodgkin-Huxley (HH) model for neuronal behaviour.[12] This model enables a description of the transients of the ionic current that produce the action potential[13,14] by using several x-type variables that represent the gating of ionic channels.[15] Eqs. (2, 4) also form the fundamental framework of the theory of memristors[16,17] where x indicates the state of a conducting filament. In perovskite solar cells, the variable x usually represents an internal voltage v or current j .[4,7]

The above demonstration, presented in further detail the Appendix, shows that the type of electrical response of the slow variable x depends on the form of the current. If the current is a time derivative of x the response is capacitive. But if the current is a direct function of x in Eq. (4) then the response is inductive. Eqs. (2, 4) can be obtained by a large variety of specific mechanisms, and the set of two equations was termed a chemical inductor,[18] which is just a generic denomination. In the framework of perovskite solar cells, a surface polarization model can be formulated in which both the capacitive current and the recombination current depend on the surface ionic charge, $Q_s(v_s)$.[19] Then both time constants for the inductor and the capacitor are the same, Fig. SI5A, which is observed experimentally.[20]

The same approach provides an explanation of basic hysteresis features. Hysteresis can be analyzed by observing the current in stable voltage cycling, or in response to a step voltage ΔV_{ap}.[21] Any delayed response in the device, such as described by Eq. (2), produces a temporary departure from the equilibrium line which gives a hysteresis effect. Typically, a capacitive process in the system produces a temporary charging current. As we show in the Appendix, under a voltage step, the current response determined by Eqs. (1, 2) is[4]

$$\Delta j(t) = g_C\, \Delta V_{ap}\, e^{-t/\tau_s} \tag{6}$$

The current decreases from an initial spike until the capacitance is fully charged when $\Delta j = 0$. In the jV diagram the total current lays below the equilibrium line $J_{rec}(V)$. This is normal or capacitive hysteresis.[22,23] Conversely, for the inductive system (2, 4) we

obtain

$$\Delta j(t) = g_L \, \Delta V_{ap} \left(1 - e^{-t/\tau_s}\right) \tag{7}$$

Now the current increases at long time, until the final value $g_L \, \Delta V$. In a jV diagram the forward current occurs above the equilibrium current. This is inverted or inductive hysteresis.[21]

Our analysis shows that any mechanistic explanation for inverted hysteresis that occurs under sustained voltage cycling, has a correspondent inductor mechanism in IS. We remark that a popular denominator of the inductive process is a "negative capacitance". However, capacitances and inductors are very different. The impedance of a capacitance has the expression Z=1/($i\omega C$) that becomes infinite at low frequencies. An inductor impedance Z=$i\omega$L becomes short-circuit at low frequency. The feature observed in perovskite solar cells is modelled with a positive inductor, not a physical negative capacitor. Negative capacitance could be desired for applications, as it could lower the power consumption in field-effect transistors, by reducing the subthreshold swing factor below the thermodynamic limit of 60 mV per decade, with enormous technological impacts.[24] Notably, we know of no case of a stable negative capacitance reported in the literature. In contrast, the chemical inductor is a common object observed across many kinds of fields.

The transients of the two kinds (6, 7) are easily observed in perovskite solar cells,[7,25] and the correlation of currents with IS is well established.[26] Note that the time constants of the impedance branches in Eq. (3) containing a capacitor is $g_C^{-1} C_s = \tau_s$ and in Eq. (5) containing the inductor it is $g_L L_a = \tau_s$. Both correspond to the time constant τ_s of the delay equation (2).

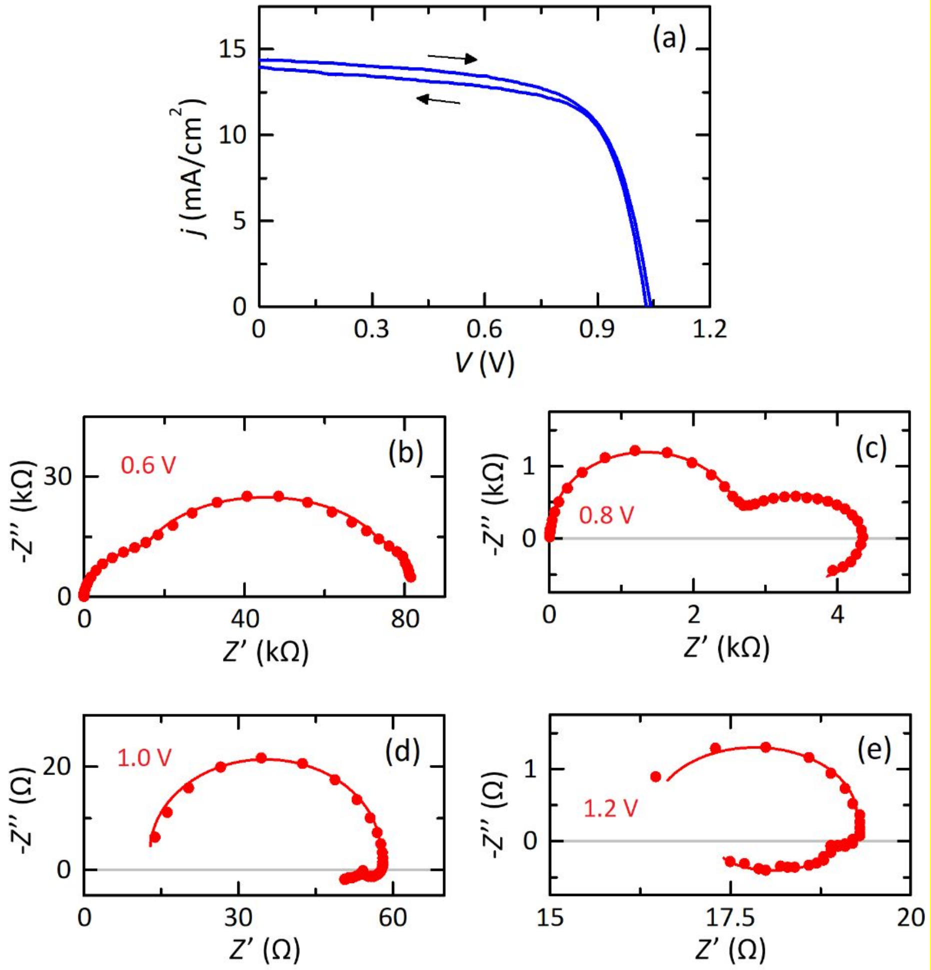

Fig. 1. Experimental responses of an inverted perovskite solar cell with a device layer architecture consisting of FTO/NiO/MeO-2PACz/perovskite ($Cs_{0.05}FA_{0.8}MA_{0.15}PbI_{2.75}Br_{0.25}$)/PCBM/BCP/Au/Ag. (a) Current-voltage curve obtained under a scan rate of 100 mV/s. Impedance spectra at (b) 0.6 V, (c) 0.8 V, (d) 1.0 V, and (e) 1.2 V under a frequency range from 1 MHz to 100 mHz.

The most common effect observed in current-voltage curves involves the occurrence of inverted hysteresis at high voltages (beyond open-circuit voltage) and capacitive hysteresis at lower voltages, corresponding, respectively, to inductive and capacitive features identified through impedance analysis.[20] These hysteresis effects can be attributed to the contacts, as small tuning of the transport layers can change the type of observed hysteresis.[27] Furthermore, in these cases the separation of the current between forward and backward scans occurs only close to V_{oc}, while in the region of low voltages the current remains horizontal, meaning that the charge collection region is not affected. Hence, models can be developed based on surface effects on the electronic carriers, for instance when ionic charge creates surface accumulation and influences the electronic current via modified surface recombination. This effect is modelled in surface polarization models and related approaches,[8,19,28,29] using slow recombination mechanisms for surface

phenomena characterized by ideality exponents.[30] An extensive survey of the literature by Nemnes et al.[8] establishes a clear distinction of capacitive and inductive recombination processes, in addition to the reference recombination current.

In other cases, however, it is possible to obtain a significant separation of the current in forward and backward close to the $V = 0\ V$ region of the current-voltage curve,[31,32] as shown in the experimental results of Fig. 1a for a solar cell under light, and in Fig. SI1 for a solar cell in the dark, which is accompanied in both cases by the observation of a double inductor feature at low frequencies, Fig. 1(d, e) and Fig. SI2. Hence, it is also frequently reported in the literature that inductive hysteresis can arise at relatively low voltages, when the current remains close to the short-circuit value.[1,2,33-35] This behavior leads to a more pronounced inverted hysteresis in the current-voltage curve, often characterized by a significant slope and forward-reverse current separation around 0 V.[35] This type of hysteresis is often interpreted according to slow ionic interfacial polarization that affects the bulk electrical field and modifies the photocurrent altering the charge collection. This mechanism has been described many times.[31,35-40] Here we provide a summary explanation based on the elementary mechanism of Fig. 2.

Fig. 2a shows a standard model for ionic-electronic diodes and solar cells.[41] The semiconductor layer, sandwiched between contact layers of different work function, contains mobile ions, so that the bulk electrical field that drives the carrier transport is reduced by dipole layers at the contacts formed by ionic accumulation at the interface. The effects of screening or augmented fields have already explained in early references[36] and it was shown that the polarity of the photovoltaic device has been switched.[42] When the device is biased out of equilibrium, a drift current occurs, Fig. 2b.

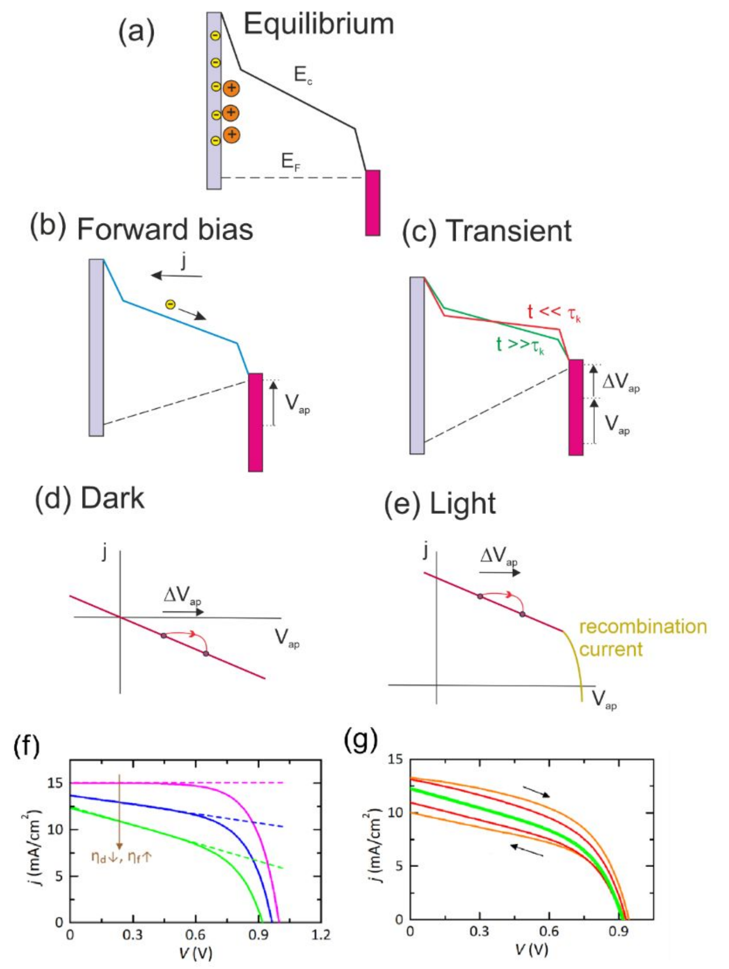

Fig. 2. (a-b) Basic model of a solar cell where carrier transport is driven by drift in the electrical field, with different biasing conditions. The dipole layer in the hole selective contact is formed by electrons (yellow) and cations (orange). (c) Effect of a change of voltage. Initially (red) the dipole layers remain in the previous configuration, since the ion rearrangement is slow. When the ions relax at $t \gg \tau_k$, the dipole layers are reduced (green), and the current increases. Thus, the model generates inverted (inductive) hysteresis in the dark, as shown in (d). (e) shows the added photogeneration, the presence of hysteresis, and the recombination current that is onset at higher voltages. (f, g) Simulation of model curves. (f) Steady state current-voltage characteristics. The vertical arrow indicates the changes of collection efficiencies: $\eta_{\mathrm{d}} = 1, 0.85, 0.7; \eta_{\mathrm{f}} = 0, 0.1, 0.2$, and the dashed straight lines show $j_{\mathrm{ph}}(V, \Phi)$. (g) Hysteresis in the green curve of (a) by increasing the sweep velocity, $f = 1$ Hz (orange), 0.5 Hz (red). Parameters $V_{\mathrm{m}} = -0.2$ V, $V_{\mathrm{p}} = 1.2$ V.

To describe hysteresis, we analyze the response to a sudden forward step voltage ΔV_{ap} shown in Fig. 2c. Immediately after the voltage step the dipole layers remain frozen (red line), as it takes some time τ_k for the excess ions to adapt to the new voltage,[43] reaching

the new equilibrium green line. The initial current is smaller than the equilibrated current. This is the footprint of an inductor behaviour. The trajectory of the current is shown in Fig, 2d. When the photocurrent is added, the same effect persists in Fig. 2e. At high voltage the recombination current (yellow) becomes dominant in Fig. 2e.

Appendix

Consider the expression of the current in a photovoltaic device

$$j = \frac{dQ_s}{dt} \tag{8}$$

Here $Q_s(x)$ is a charge function that depends on an internal variable x. Hence

$$j = C_a(x)\frac{dx}{dt} \tag{9}$$

where $C_a = dQ_s/dx$. This internal variable depends on the applied voltage V by the equation

$$\tau_s\frac{dx}{dt} = F(V) - x \tag{10}$$

In equilibrium it is $x = F(\bar{V})$. The x depends on voltage through the nonlinear function F. The small signal ac version of (12, 13) is

$$\hat{j} = i\omega\, C_a\, x \tag{11}$$

$$\tau_s i\omega\, x = f(\bar{V})\hat{V} - x \tag{12}$$

Here $f = dF/dV$, x is the steady state value, and x the small expansion value. Hence the impedance is

$$Z = \frac{\hat{V}}{\hat{j}} = \frac{1}{g_C} + \frac{1}{C_s\, i\omega} \tag{13}$$

This is a series RC circuit with the conductance and capacitance

$$g_C = \frac{C_s}{\tau_s}, \qquad C_s = f\, C_a \tag{14}$$

If we include in Eq. (11) the recombination current $J_{rec}(V)$, a parallel recombination resistance $R_{rec} = (dJ_{rec}/dV)^{-1}$ will be obtained.

Now we make a different assumption with respect to (11). The current has the form

$$j = j_a(x) \tag{15}$$

for a nonlinear function j_a and an internal variable x also controlled by Eq. (13). For a small expansion we have

$$\hat{j} = m(\,x)\, x \tag{16}$$

where $m(x) = dj_a/dx$. In combination with (15), we find the impedance expression

$$Z = \frac{1}{g_L} + L_b\, i\omega \tag{17}$$

This is a series of a resistor and inductor, with the values

$$g_L = m\, f, \qquad L_b = \frac{\tau_s}{m\, f} \tag{18}$$

The delay equation (13) causes hysteresis, which is associated with the transient

behaviour of the current under a voltage step $\overline{V}\rightarrow\overline{V}+\Delta V_{ap}$.[21] Integration of Eq. (13) gives[4]

$$\Delta v_s = f\,\Delta V_{ap}\,(1-e^{-t/\tau_s}) \tag{19}$$

For the capacitive system (11) we obtain

$$\Delta j(t) = g_C\,\Delta V_{ap}\,e^{-t/\tau_s} \tag{20}$$

For the inductive system (18) it is

$$\Delta j(t) = g_L\,\Delta V_{ap}\,(1-e^{-t/\tau_s}) \tag{21}$$

2. New experimental evidence showing the double inductor in connection with inductive hysteresis in the current is shown, under illumination (Fig. 1) and in the dark (Fig. SI1 and SI2).

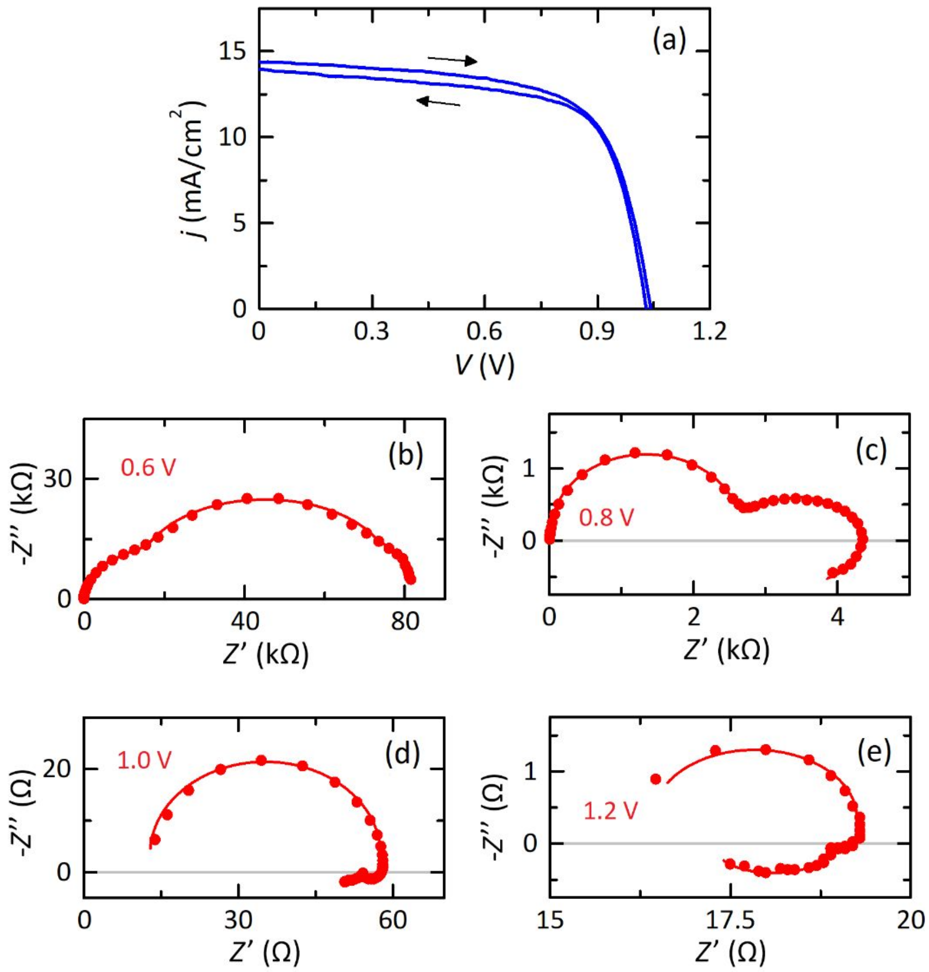

Fig. 1. Experimental responses of an inverted perovskite solar cell with a device layer architecture consisting of FTO/NiO/MeO-2PACz/perovskite ($Cs_{0.05}FA_{0.8}MA_{0.15}PbI_{2.75}Br_{0.25}$)/PCBM/BCP/Au/Ag. (a) Current-voltage curve obtained

under a scan rate of 100 mV/s. Impedance spectra at (b) 0.6 V, (c) 0.8 V, (d) 1.0 V, and (e) 1.2 V under a frequency range from 1 MHz to 100 mHz.

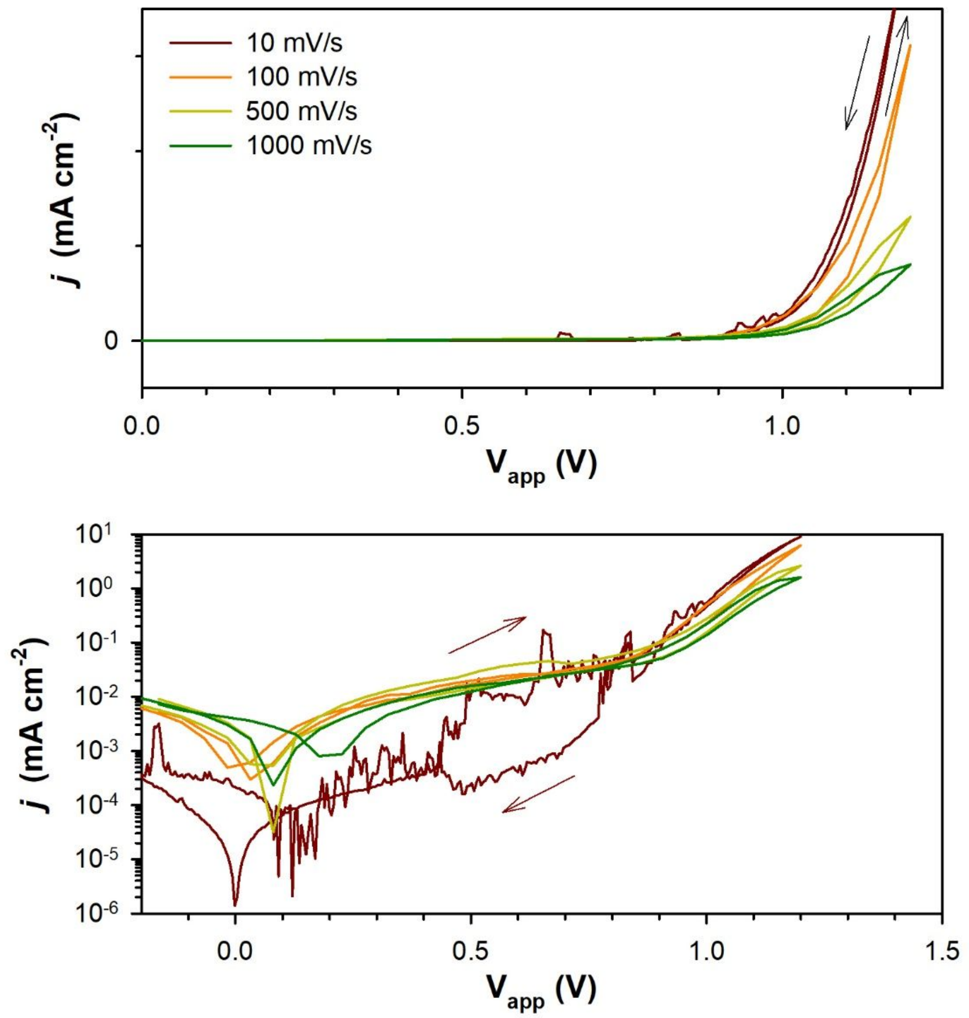

Fig. SI1. *J-V* response of a perovskite solar cell FTO/SnO_2/Perovskite/Spiro-OMeTAD/Au with the SnO_2 layer prepared by atomic layer deposition (ALD) measured under dark conditions and as a function of the scan rate. Linear (top) and semi-log (bottom) scale graphs are represented for clarity.

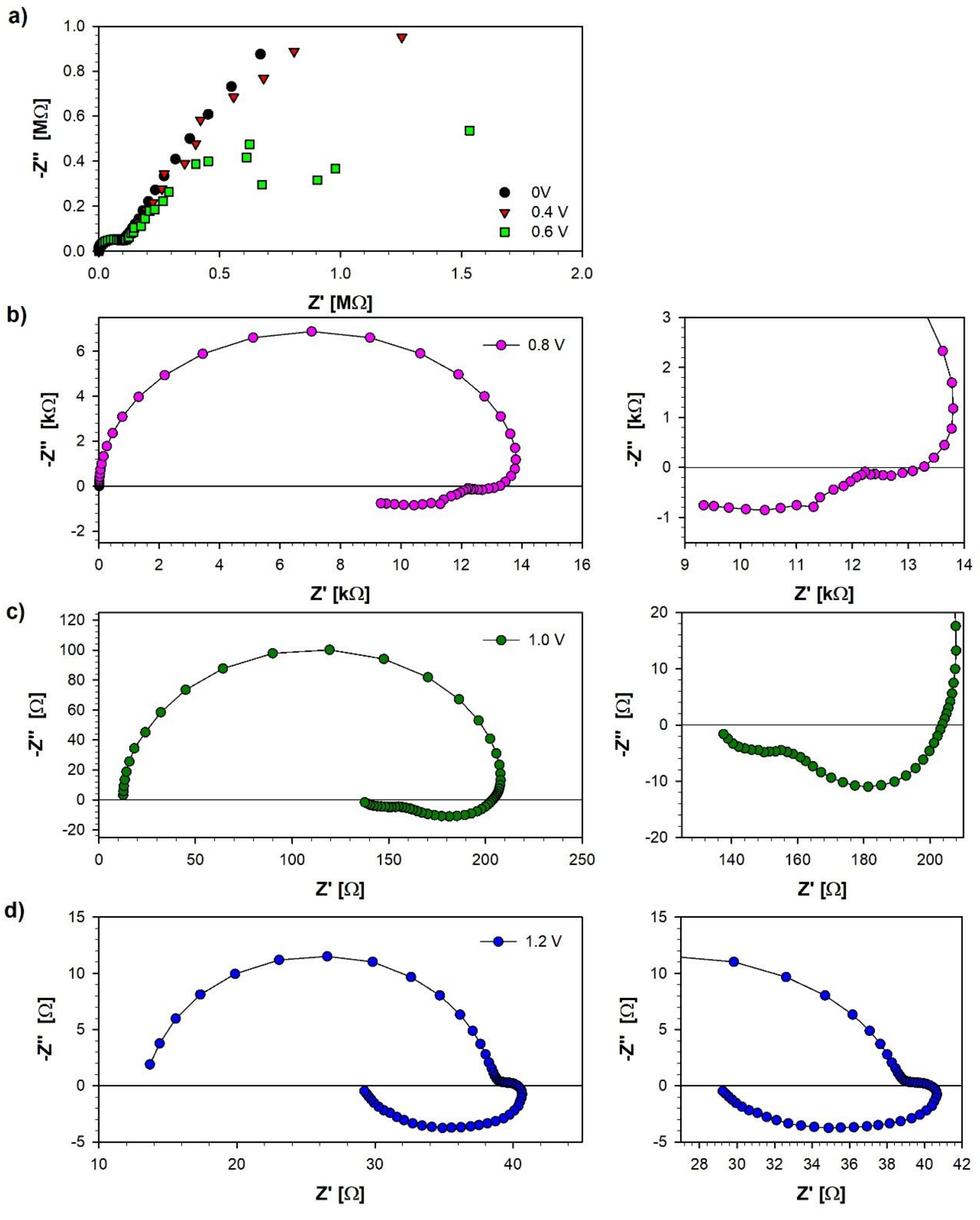

Fig. SI2 Complex impedance plot of measurements of a perovskite solar cell FTO/SnO_2/Perovskite/Spiro-OMeTAD/Au with the SnO_2 layer prepared by atomic layer deposition (ALD) measured in the dark under different DC voltages with. a) V_{DC} = 0 V, 0.4 V and 0.6 V, b) V_{DC} = 0.8 V, c) V_{DC} = 1.0 V and d) V_{DC} = 1.2 V. The insets on the right focus on the inductive features.

3. The limitations of the model and relation with alternative approaches are clearly

discussed.

In the following, we want to take a moment to discuss limitations and improvements of the model. The separation of contact polarization and recombination on the one hand, and bulk charge collection, on the other, is useful in many situations. The ionic charge is mainly located in the surface, affecting both surface recombination and the electrical field driving transport. In the above model we highlighted the direct effect of the electrical field on the photocurrent. This approach assumes that transport and recombination are effectively decoupled, which is a good approximation in many situations.[44] In experimental investigations of IS, halide perovskite solar cells produce varied behaviours, which is very natural considering the enormous diversity of composition, materials, morphology, types of contacts, and external conditions of the solar cells that enable chemical exchanges. In the present paper we have provided an interpretation of the bulk inductor added to the surface recombination components using a single voltage model. In a specific experimental investigation, the impedance can be described using different combinations of series/parallel circuits that distinguish bulk and surface effects,[19,20,34,45] as shown in Fig. SI5.

In addition, more complex situations and mechanisms may arise than a simple series ordering of elements. This is obtained experimentally by correlations of the measured parameters. The surface dipole can extend into the bulk of the perovskite layer. The seminal work of Garcia-Belmonte[46] showed that the low frequency interfacial capacitance includes components from both double layer and ionic transport charging. The transport layers may produce interference with the measured time constants.[47,48] It has also been remarked that the influence of ions in charge collection also affects surface recombination.[8] The buildup of ions can strengthen the internal drift field within the bulk, but it may also create unfavorable reversed electric field regions at the interfaces, where carriers become trapped and subsequently recombine via surface states.[49] These situations can be analyzed using spatially resolved models, [50-54] that describe the internal spatial variation of Fermi levels, using transport equations for all the carriers, the Poisson equation, and charge transfer conditions to the transport layers. These methods provide a consistent solution to the complex questions of ionic-electronic mixed conductance, charge compensation, and their influence on recombination, as discussed in Sec. S4 of the SI. Furthermore, electrochemical and photochemical reaction features, including the complex defect chemistry of halide perovskites[55-57] become necessary to understand the phenomena. These methods become particularly useful for experimental analysis of device degradation.

4. Extended discussion on modelling and interpretation in the SI

S4. Interpretation of voltages and model assumptions

The model of Eqs. (T1-T5) is controlled by a single device voltage. The chemical inductor and variable capacitance terms generate the same type of circuit element: A series connection of a resistor and a reactive element (either inductor of capacitor), as explained in the Appendix.

In our model we have made the definition that both voltage variables equilibrate to the same voltage: $v_s \to V$, $v_b \to V$. This does not mean that both internal voltages coincide with the whole external voltage V. There may be a constant relation, such that $v_s \to V - V_{s0}$, for instance. However, this will only introduce another constant parameter V_{s0} that we cannot measure, since it is absorbed by the prefactor of the capacitance. In the past, we have developed a fully consistent model for dc and ac polarization, based on the band diagram, as shown in the Fig. SI5.A.[19] This model contains the variable v_s both in the capacitance and in the slow recombination term of the current.

However, many devices contain a neat separation between the voltage in the bulk and the voltage in the contact, and this is also expressed in the diagrams of Fig. 2. Then one should distinguish a parallel mode (with the individual branches all controlled by V) or a series model, where a partition $V = V_1 + V_2$ applies to different subcircuits, or a combination of both. We have devoted many discussions to this question.[58] In Fig. S5 we show a range of models that distinguish the bulk and surface effects, with the presence of inductors and capacitors in different places:

A. Impedance model from a surface polarization model. Here all the branches are in parallel.[19]

B. Model and impedance spectra of a MAPbBr perovskite solar cell.[20] The model distinguishes bulk (R_3) and surface elements, where the inductor is for surface recombination.

C. Model and impedance spectra of $FA_{0.85}$ $MA_{0.15}$ Pb(I 0.85 $Br_{0.15})_3$) perovskite solar cell.[34] The model distinguishes bulk and surface elements, where the inductor is in the bulk component.

D. Model and measurement of a carbon-based perovskite solar cell.[45] The model distinguishes bulk (R_3) and surface components. The surface capacitor and inductor are described by **A**. An additional RC branch is added to account for the three observed capacitance plateaus.

**A**

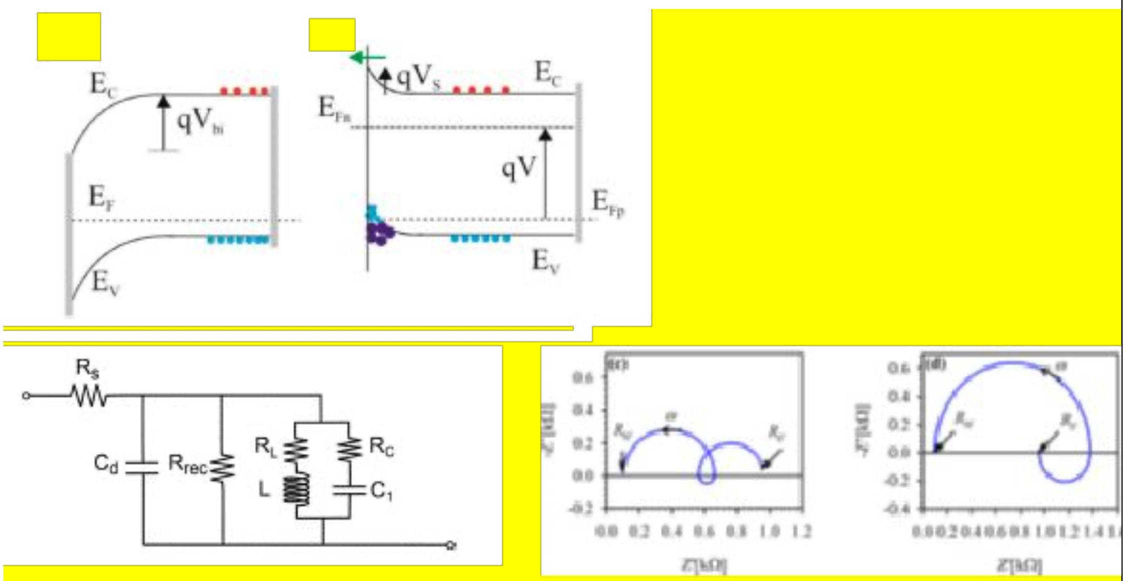
E_C
qV_bi
E_F
E_V
qV_s
E_C
E_Fn
qV
E_Fp
E_V
R_s
C_d
R_rec
R_L
L
R_C
C_1

B

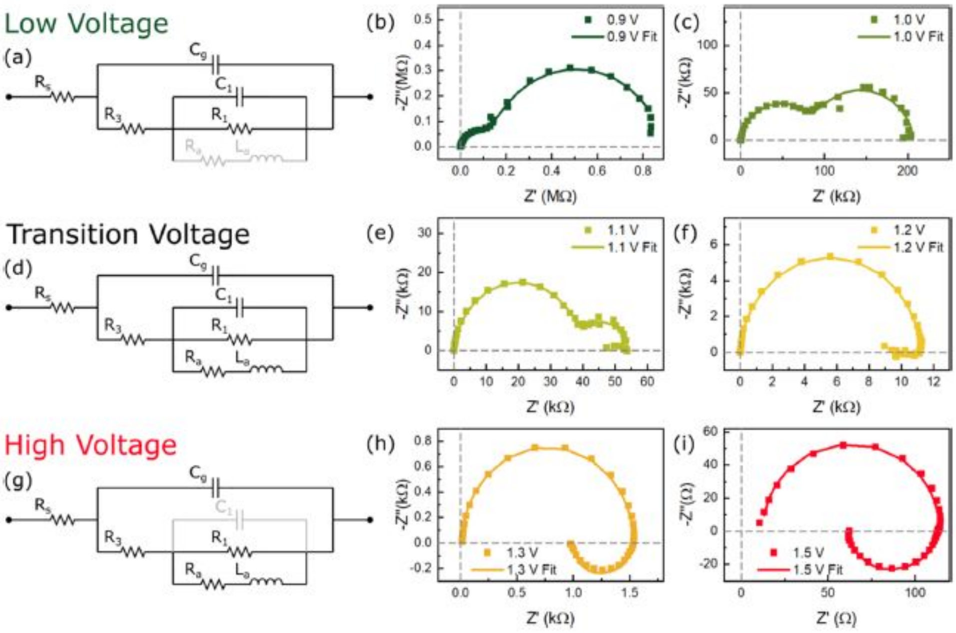
Low Voltage
(a)
Transition Voltage
(d)
High Voltage
(g)
(b)
0.9 V
0.9 V Fit
-Z''(MΩ)
Z' (MΩ)
(c)
1.0 V
1.0 V Fit
-Z''(kΩ)
Z' (kΩ)
(e)
1.1 V
1.1 V Fit
(f)
1.2 V
1.2 V Fit
(h)
1.3 V
1.3 V Fit
(i)
1.5 V
1.5 V Fit
Z' (Ω)

C

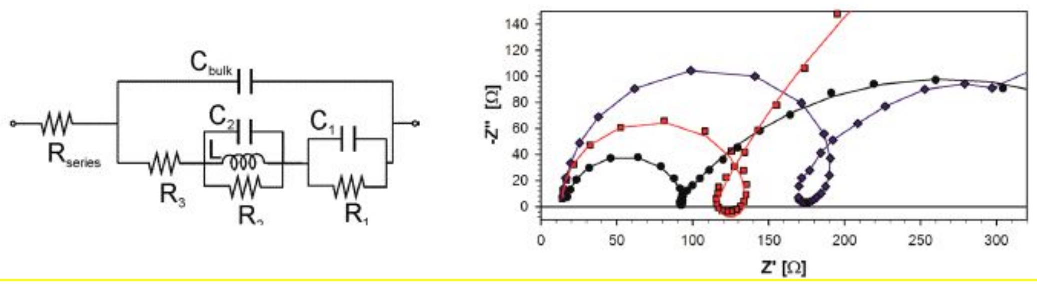

D

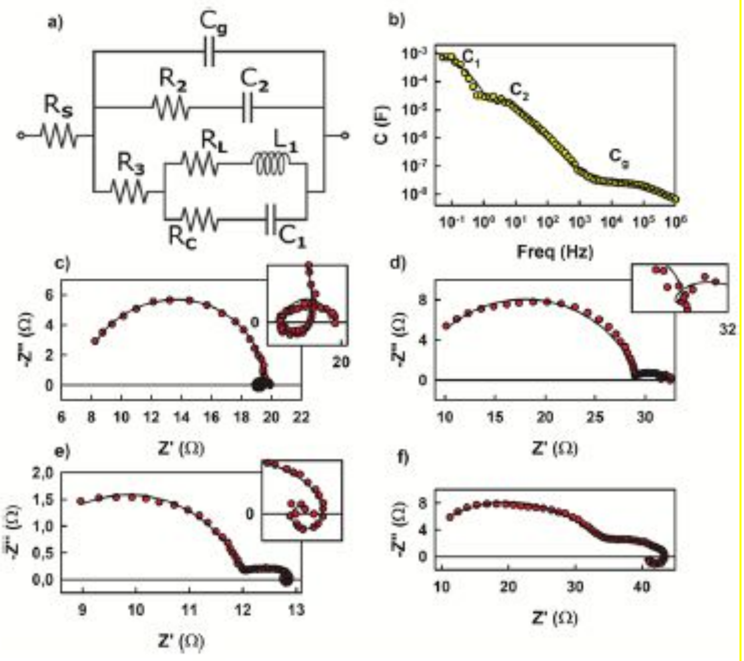

Fig. SI5. **A**. Impedance model from a surface polarization model.[19] **B.** Model and impedance spectra of a MAPbBr perovskite solar cell.[20] **C.** Model and impedance spectra of $FA_{0.85}$ $MA_{0.15}$ $Pb(I\ 0.85\ Br_{0.15})_3)$ perovskite solar cell.[34] D. Model and measurement of a carbon-based perovskite solar cell.[45]

The solar cells in Fig. SI5 produce varied behaviours, which is very natural considering the enormous diversity of composition, materials, and morphology, that can be obtained with halide perovskite solar cells. At present we find more practical for analysis of impedance spectroscopy results to use a simpler expression of the differential equations that goes straight to the impedance parameters. This first order approximation to the problem can be completed with more detailed approaches is the situation allows more work or merits further investigation. In the present paper we have provided an interpretation of the bulk inductor added to the surface recombination components using a single voltage model. In a specific experimental investigation, the impedance can be

investigated using different combinations as shown in Fig. SI5.

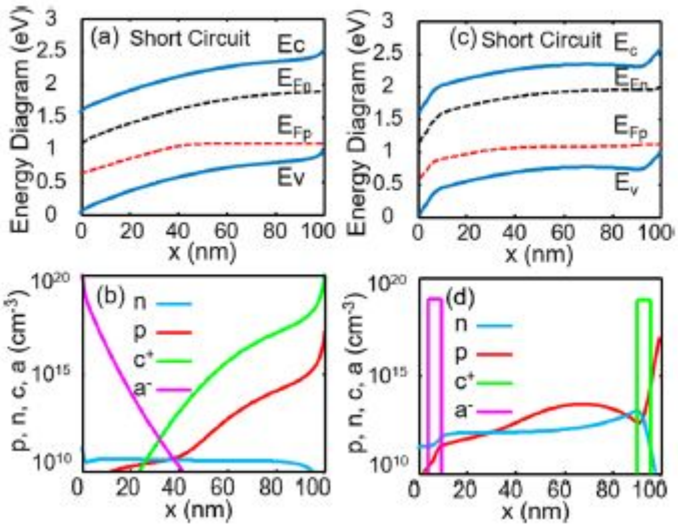

Fig. SI6. Energy band diagram and distributions of ions, electrons, and holes for a p-doped perovskite solar cell (with electron and hole selective contacts in the left and right side, respectively) under illumination in short-circuit steady state, (a, b) including drift-diffusion of ions in the numerical simulation and (c, d) assuming a fixed dipolar-distribution of ions in the numerical simulation.[59]

There is also another effect affecting the voltage distribution, associated to the complex mixed ionic-electronic nature of the halide perovskites. Consider the surface barrier in Fig. SI5A. It is not clear if this barrier is at the contact or penetrates the bulk region. Fig. SI6 shows the complex charge-compensation distribution that may appear (case b) instead of simple ionic-electronic charge accumulation at the interface (case d, corresponding to Fig. 2). A wide variety of situation according to the ionic charge balance may occur.[60] Capacitive methods enable the study of the interface charging.[46,61] But even the simplest Mott-Schottky method to determine ion concentration and flatband potential is fully problematic in perovskite solar cells.[62,63] In general, if there is a considerable number of injected charges, the traditional vision of a surface depletion region (or dipole layer) determining the surface capacitance collapses.[64] One finds a catalogue of complex transients and transport effects involving the ion and electron transport coupling, due to the mobility of the "doping" ions and electrical field screening.[61,65-67] Then it is not easy to define a priori a bulk and a contact potential.

Since many years the separation of bulk and surface impedance (the latter mainly determined by effects of the transport layers) has been studied through modifications of contact layers, device thickness, and light soaking conditions.[34,44,54,68,69] The seminal work of Garcia-Belmonte[46] showed that the low frequency interfacial capacitance includes components from both double layer and ionic transport charging. Recently, new

methodologies have been developed by Ravishankar et al to establish the influence of charge transport layers.[47,48] These works shows limitations of impedance to detect different intrinsic time constants, so that transient decays analysis needs to be combined with the frequency methods.

The models based on a simple set of Eqs. like (T1-T5), only recognize a spatial region with two contacts and one voltage. One can form series models, with two distinct regions, associated to a distinction of bulk and contact impedances, as we have shown in Fig. SI5. However, the reality is that mixed ionic-electronic transport and charge compensation creates a huge variety of situations, as indicated in Fig. SI6.

The questions about ions distribution and their effect on performance are much better answered using a model that considers all the distinct points in the device, using transport equations for all the carriers, the Poisson equation, and charge transfer conditions to the transport layers.[48,70] Recently there is progress in impedance models that are fully spatially resolved.[50-54] The advantage of these methods is that they may provide information of what effects are caused in the impedance by the complicated combinations of carriers and layer distributions. One example is the analysis of large photoinduced capacitances.[71]

The development of simulation models has the mentioned advantages, but also in the present form some important limitations. In our view the aim of the analysis of impedance spectroscopy is not just "explaining" spectra, but tracking the evolution of internal parameters over changing external parameters like current, light, voltage, gasses influx, and device composition or contact layers. Large amount of the data must be fitted and the variation of parameters needs to be understood by materials and geometry modifications, that sometimes produce changes that one does not expect, due to complex internal interactions. So far, the numerical methods are often not used in this way, and they mainly "explain" results of impedance spectra. Maybe these methods can be enhanced in the future into high throughput real data treatment, since techniques are being developed to extract parameters from experimental data.[72] However, the mainstream of impedance spectroscopy in all kinds of research fields uses equivalent circuit for such data treatment, with software that is adapted to the measuring equipment like Zview. The last generation software approaches https://rhd-instruments.de/solutions-and-products/for-eis-data-analysis/relaxis/ continue adopting equivalent circuits as the main tool, and the AI-assisted methods go in the direction of self-fitting.

A practical problem associated with drift-diffusion methods is the multiplicity of solutions. Impedance spectroscopy contains very limited information with respect to all the internal details of orthodox semiconductor modelling, since in the end only a ratio V/I is calculated. Thus, there are a great range of assumptions leading to the same impedance spectra. This is not an intrinsic limitation, it is in the nature of the measuring technique, however it is a warning that one cannot take a specific model as the main explanation.

For example, Refs [51,73] explain that "the switching between electron-limited and hole-limited bulk recombination causes a change in the sign of the LF feature when the cell is otherwise unchanged. This is based on the Equation[73]

$$C_{\mathrm{LF}} = \frac{n_{\mathrm{ap}}(V_{\mathrm{dc}}) j_{\mathrm{rec}}(V_{\mathrm{dc}})}{G_{+} V_T n_{\mathrm{el}} \left(n_{\mathrm{ap}}(V_{\mathrm{dc}}) - n_{\mathrm{el}}\right)} \left(-\frac{dQ_{\mathrm{dc}}}{dV_{\mathrm{dc}}}\right).$$

in which the low frequency capacitance can become negative.

As discussed in the main text, strictly negative capacitances have not been observed to our knowledge. The feature observed in perovskite solar cells and denoted in the popular jargon "negative capacitance" is a positive inductor, not a negative capacitor.

The inductor is explained [51,73] "changing the bulk SRH recombination from electron-limited to hole-limited." This is one mechanism, which is enabled by drift-diffusion equations. In another explanation "the transport layer permittivities have been swapped to be consistent with an inorganic ETL and an organic HTL".[51]

Yet in another paper the authors explain inverted hysteresis (which surely generates an inductor) with a charge imbalance related to ion charge, that generates substantial electrical field.[53] This is obtained by the equations

$$D_P N_0 \frac{\partial^2 \phi}{\partial x^2} = \frac{d_H g_v}{g_v^H} \exp\left(\frac{V(t)-V_{bi}-2\phi}{2V_T} + \frac{E_v - E_v^H}{qV_T}\right) \frac{\partial}{\partial t}\left(\phi - \frac{V(t)}{2}\right) - \frac{\varepsilon_P V_T}{q} \frac{\partial}{\partial t}\left(\frac{\partial^2 \phi}{\partial x^2}\right. \quad \text{(27a)}$$

which couples to the perovskite boundary layer charge densities via the ODEs

$$\frac{dQ_L}{dt} = \frac{qD_P N_0}{V_T} \frac{\partial \phi}{\partial x}\Big|_{x=0^+}, \quad \text{(27b)}$$

$$\frac{dQ_R}{dt} = -\frac{qD_P N_0}{V_T} \frac{\partial \phi}{\partial x}\Big|_{x=b^-}, \quad \text{(27c)}$$

that generates a slow response of the internal electrical field. Whether these mechanisms obtained in the equations corresponds to what happens in reality is not justified in real case experiments, for example "swapping the transport layer permittivities".

Obtaining different models that generate the same impedance response is a positive contribution; however, one may ask what these models have in common? Is there a unifying mechanism behind them? This is answered by the elementary model of the chemical inductor, that establishes fundamental conditions to obtain the inductive response in impedance and hysteresis. However, the model only provides two parameters, a resistance and a relaxation time. Then there is an important issue of obtaining an interpretation of such parameters in terms of specific physico-chemical properties of the device. This is when microscopic-based transport/reaction models become very important tools. We highlight the work by Anta and coworkers that combines full analysis of the experimental data with equivalent circuits under changing materials conditions, and the spatially-resolved simulation for understanding the parameter evolution.[54,74,75]

Another limitation is that the drift-diffusion methods tend to explain spectral shapes with the orthodox semiconductor modelling equation, based on charge compensation, transport etc. However, halide perovskites are unconventional materials from this point

of view that contain other factors such as electrochemical reactions, that are hard to model from the orthodox approach, and are not so far described in those methods as far as we know. These effects are very important for degradation studies. Organic layers may lose performance, contact ions may leak into the perovskite layer.[76-79] Reactivity conditions can be identified in the low frequency region of the capacitance plots.[76]

In summary, the model (T1-T5) is a zero-order model for the halide perovskite solar cells that can be completed in several respects.

(a) Correlation of techniques. Impedance sometimes cannot show relevant information that the system contains. In some cases transport features are hidden,[80,81] and in other cases nonlinear response becomes large and relevant for application.[82] Different small signal modulated techniques can be combined,[80,83] and time domain small or large perturbation methods provide essential information.[5-7,84-86]

(b) Transport layers and charge compensation. Among the many different aspects of the impedance of perovskite solar cells, we find special complexity in the analysis of transport layers and large charge densities close to them, as commented above. Here the neuron-style models give a first guidance of the observations, but other methods are needed.

(b1) Experimental methods can be developed that probe specifically interfacial ionic-electronic charging[46,61,83,87,88] and the effective time constants of the transport layers. [47,48]

(b2) Under strong inhomogeneous conditions the previous methods need to be complemented with detailed spatially resolved modelling techniques, possibly combined with electrochemical and photochemical reaction features.[50-54,74,75]

(b3) Electrochemical and photochemical reactions and complex defect chemistry[89,90] form a substantial part of ionic-electronic dynamics in halide perovskites.[90] These phenomena need to be considered in the interpretation of experiments.[91,92]

5. Discussion on drift and diffusion transport in the SI

S5. Diffusion and drift transport

Electrons and ions in a perovskite solar cell are transported by drift in the electrical field and diffusion, according to concentration gradients. In this paper we have selected only variations of drift transport to describe charge transport limitations across the bulk layer, Eq. (T2). The electrical field modification under bias is reflected in a tilt of the semiconductor bands that modifies the charge velocity. This is observed in many cases, in the form of a linear variation of the *IV* curve around short circuit voltages, Fig. 2, and the associated effects of field screening by mobile ions have been amply discussed.

The observation of diffusion by impedance spectroscopy by transmission line methods

is more reliable if one carrier dominates in the mixed ion-electronic conduction process. When diffusion is the dominant transport mechanism it shows a well-known impedance pattern formed by a transmission line model.[93] When there are two carriers with varying concentration or mobilities the impedances escalate into very complex patterns.[94] Nontrivial boundary conditions introduce a terminal impedance.[95,96] The boundary can have enormous effect on the impedance if it allows partial transmission of charge into the transport layer at the contact. The boundary impedance is neither in series nor parallel with the bulk transmission line.

The electronic transport by diffusion was easy to observe in dye-sensitized solar cells by transmission line methods,[97] due to slow electron transport in mesoporous TiO_2. In contrast, electron mobility in halide perovskite solar cells is much higher, and the diffusion resistance becomes small. It has been possible to observe electron diffusion by IMPS, using very thick samples and non-uniform charge generation.[98] It occurs in the very high frequency domain, by a crossover to the negative Q' axis, as shown in Fig. SI7.[99,100]

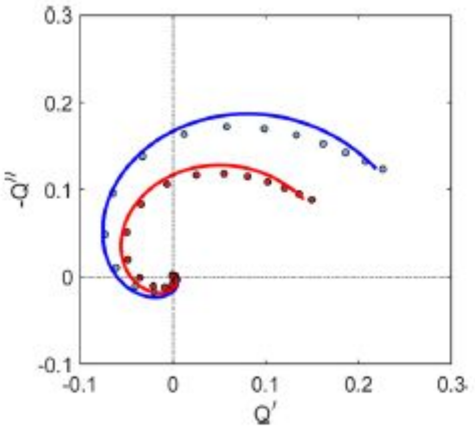

Fig. SI7. IMPS data (point) and fit (line) for a perovskite cell of 5.3 μm thickness illuminated with blue and red light, with an estimated absorption length of 40 and 140 nm, respectively.[98]

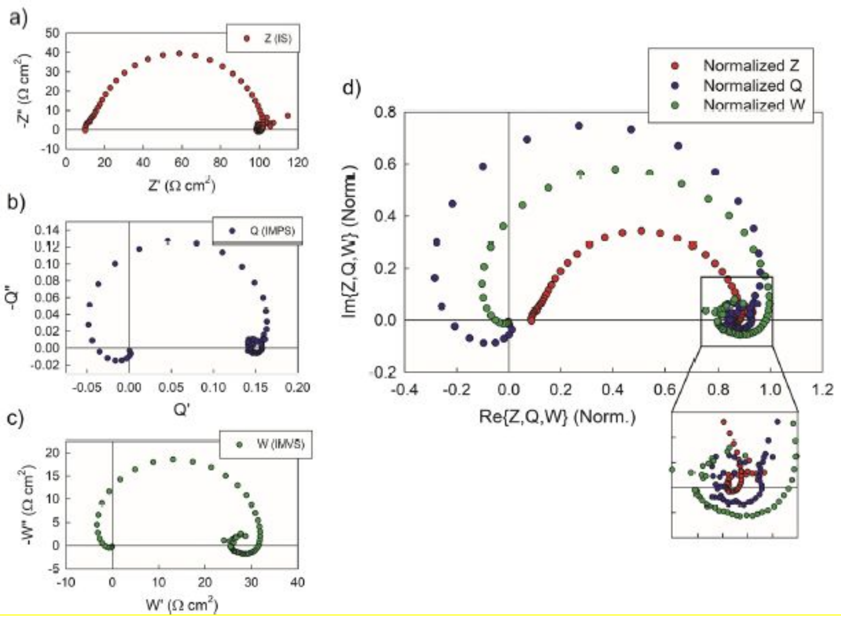

Fig. SI8. Experimental complex plane plots of (a) IS, (b) IMPS, and (c) IMVS for a carbon-based perovskite solar cell measured with illumination of 0,11 sun and at open circuit voltage (V = 0.85 V). (d) shows the three normalized spectra together.[80]

Further studies have correlated the different techniques.[80] It is confirmed that the high frequency electron diffusion feature is not observed in impedance spectroscopy, while it is detected both in IMPS and IMVS. We conclude that diffusion is perovskite solar cells cannot be normally observed in impedance spectroscopy results.

Point-by-point response

Reviewer: 1

Comments:
In this manuscript, the authors present a model that incorporates the effects of collection efficiency and different types of recombination in perovskite solar cells. They simulate the device response under both current-voltage scans at varying frequencies and impedance spectroscopy measurements. The model successfully reproduces a wide range of observed features, particularly the double low-frequency inductive loops and intermediate inductive responses.

While the ability to reproduce these features is notable and constitutes a valuable contribution, several key aspects of the model require clarification and refinement before the manuscript can be considered for publication:

Comment 1. Internal Voltages (v_b and v_s):
The model introduces two internal voltages: v_b, associated with bulk ion field screening, and v_s, linked to ionic accumulation at the surface. Both voltages follow similar relaxation dynamics and relax toward the applied voltage. However, this raises significant concerns. From a phenomenological standpoint, it is unclear why two distinct internal voltages would independently relax to the same external value. If v_b and v_s both equal the applied voltage, their distinction becomes ambiguous. A schematic representation of the internal voltage distribution within the device is necessary to clarify how these voltages are defined and how they relate to each other and to the applied bias. Although for impedance analysis the specific relaxation value may not change the equivalent circuit, from a physical modeling perspective, this formulation needs better justification.

Authors Response
Thank you for this important comment. It is commented in Section S4. Interpretation of voltages and model assumptions

Comment 2. Surface Recombination vs. Surface Voltage:
The manuscript title emphasizes the combined effect of collection efficiency and surface recombination. However, the model implements two types of recombination: a fast and a slow component, with the slow one governed by an additional relaxation equation. The connection between this slow recombination process and the previously defined surface voltage v_s is not made clear. If v_s is intended to represent surface phenomena, how does it not couple directly to surface recombination? The model appears to treat v_s as purely capacitive, which contradicts the interpretation that it should modulate recombination processes. The authors should clarify the physical basis for associating the

slow recombination term with surface effects and explicitly relate it to the dynamics of v_s, or revise the terminology if this association is not intended.

Authors Response

Thank you for this important comment. The model starts with the assumption of independent pathways. As the reviewer points out, in many cases the inductive recombination and surface polarization are intimately coupled. This is pointed out in the paper. In many cases, one observes experimentally that the time constants coincide. In other cases they separate, as shown in Fig. SI3.f. Thus, the matter must be decided by experimental observations.

Modification to the paper

In the framework of perovskite solar cells, a surface polarization model can be formulated in which both the capacitive current and the recombination current depend on the surface ionic charge, $Q_s(v_s)$.[19] Then both time constants for the inductor and the capacitor are the same, Fig. SI5, which is observed experimentally,[20] Fig. SI6.

Comment 3. Model Partitioning – Collection vs. Recombination:

The model is partitioned into two separate components: one governing collection efficiencies due to diffusion and transport, and another describing recombination dynamics. While this separation may simplify impedance analysis, it potentially overlooks the fact that collection efficiency inherently depends on recombination processes, particularly through diffusion-limited transport. The manuscript should address how this decoupling is justified, and whether it leads to internal inconsistencies in the physical interpretation of the model.

Authors Response

Yes, this is true, it is a possibility. Diffusion limited recombination is very often used in organic solar cells, but in perovskite solar cells the dominant coupling is normally due to ionic-electronic effects, and the collection and recombination can be separated.[44] We have included a discussion of this assumption

Modification of the main text

In the following, we want to take a moment to discuss limitations and improvements of the model. The separation of contact polarization and recombination on the one hand, and bulk charge collection, on the other, is useful in many situations. The ionic charge is mainly located in the surface, affecting both surface recombination and the electrical field driving transport. In the above model we highlighted the direct effect of the electrical field on the photocurrent. This approach assumes that transport and recombination are

effectively decoupled, which is a good approximation in many situations.[44] In experimental investigations of IS, halide perovskite solar cells produce varied behaviours, which is very natural considering the enormous diversity of composition, materials, morphology, types of contacts, and external conditions of the solar cells that enable chemical exchanges. In the present paper we have provided an interpretation of the bulk inductor added to the surface recombination components using a single voltage model. In a specific experimental investigation, the impedance can be described using different combinations of series/parallel circuits that distinguish bulk and surface effects,[19,20,34,45] as shown in Fig. SI5.

In addition, more complex situations and mechanisms may arise than a simple series ordering of elements. This is obtained experimentally by correlations of the measured parameters. The surface dipole can extend into the bulk of the perovskite layer. The seminal work of Garcia-Belmonte[46] showed that the low frequency interfacial capacitance includes components from both double layer and ionic transport charging. The transport layers may produce interference with the measured time constants.[47,48] It has also been remarked that the influence of ions in charge collection also affects surface recombination.[8] The buildup of ions can strengthen the internal drift field within the bulk, but it may also create unfavorable reversed electric field regions at the interfaces, where carriers become trapped and subsequently recombine via surface states.[49] These situations can be analyzed using spatially resolved models, [50-54] that describe the internal spatial variation of Fermi levels, using transport equations for all the carriers, the Poisson equation, and charge transfer conditions to the transport layers. These methods provide a consistent solution to the complex questions of ionic-electronic mixed conductance, charge compensation, and their influence on recombination, as discussed in Sec. S4 of the SI. Furthermore, electrochemical and photochemical reaction features, including the complex defect chemistry of halide perovskites[55-57] become necessary to understand the phenomena. These methods become particularly useful for experimental analysis of device degradation.

Comment 4. Inductive Features and Illumination Conditions:

One of the inductive features attributed to the bulk voltage appears in the photocurrent term of the model. However, Figure 3a is reproduced from a reference in which the measurements were performed under dark conditions. This raises concerns because the corresponding inductive feature in the model depends on the photocurrent, which would not be present in the dark. Empirically, it is common to observe inductive features in perovskite devices without illumination. Furthermore, the references cited by the authors to support the existence of two inductive features are based on simulations rather than experimental data. While one cited work (https://doi.org/10.1021/acsenergylett.7b00542) does report a double inductive response under illumination, the model should be adapted to account for such features independently of the photocurrent, particularly if it aims to describe dark conditions accurately.

Authors Response

Thank you for pointing out this. To simplify the discussion, we have not separated the dark current and photocurrent, the total current is termed j_{ph}. The drift term is also present in the dark, and the same effect can occur.

Modification of the main text

In a simple model with constant electrical field along the bulk of the absorber layer, as in Fig. 2, we suggest that the transport current j_{ph} can be described, starting from the fundamental basis, as

$$j_{\mathrm{ph}}(V,\Phi) = J_{\mathrm{ph0}}(\Phi)\left[\eta_{\mathrm{d}} + \eta_{\mathrm{f}}\left(\frac{V_0 - V}{V_0}\right)\right] \quad (22)$$

Here, V_0 is an effective built-in voltage under stationary operation. It corresponds to a specific steady state of bias voltage, illumination, etc. This V_0 may further depend nonlinearly on ionic components, as described by Nemnes et al.[101] in a model imported from ferroelectric systems that show large hysteresis effects.[102] $J_{\mathrm{ph0}}(\Phi)$ is the charge generated under the incoming photon flux Φ, η_{d} is the efficiency of charge collection by diffusion, η_{f} is the charge collection efficiency by the drift field. Then, the photocurrent at short circuit is $j_{\mathrm{ph}}(0,\Phi) = J_{\mathrm{ph0}}(\Phi)(\eta_{\mathrm{d}} + \eta_{\mathrm{f}})$ where $0 \leq \eta_{\mathrm{d}} + \eta_{\mathrm{f}} \leq 1$. In the dark one expects $V_0(\Phi = 0) = 0$, unless some persistent polarization occurs, and $J_{\mathrm{ph0}}(\Phi = 0)$ is a constant that depends on the mobility and the dark carrier density.

5. Novelty and Prior Work:

While the model presents novel aspects, the manuscript cites two prior works as experimental evidence when they are in fact theoretical studies that have already reproduced similar features. The authors should clearly delineate what is new in their approach compared to the models in https://doi.org/10.1002/aenm.202400955 and https://doi.org/10.1021/acs.jpclett.4c02343. This includes highlighting either improved physical insight, broader feature reproduction, or experimental validation.

Authors Response

Thank you, the references were incorrectly cited at that point. All the references have been revised and previous experimental evidences are cited accordingly. We have made an extended discussion of the different approaches, including drift-diffusion methods, in Section S4. Interpretation of voltages and model assumptions

Modification of the main text

These situations can be analyzed using spatially resolved models, [50-54] that describe the internal spatial variation of Fermi levels, using transport equations for all the carriers, the Poisson equation, and charge transfer conditions to the transport layers. These methods provide a consistent solution to the complex questions of ionic-electronic mixed

conductance, charge compensation, and their influence on recombination, as discussed in Sec. S4 of the SI.

Reviewer: 2

Comment 1. What is the major advance reported in the paper?
This manuscript provides a mathematical model to explain an unusual experimental observation: two inductor-like features at low frequencies in perovskite devices. The model proposes that these inductive characteristics are the result of two distinct physical processes occurring within the system: ion-mediated charge recombination and electric field screening by those same charges, claiming the utility of the findings as a tool to identify degradation processes in perovskite solar cells.

Comment 2. What is the immediate significance of this advance?
This referee recognizes the importance of clarifying the various impedance features observed in perovskite solar cells, especially those related to ionic motion, given their critical role in degradation mechanisms.
However, while the mathematical model presented is robust, there's a notable lack of experimental evidence to sustain the findings. Simulations are an excellent tool for predicting behaviors, but without experimental confirmation, the reliability of the claims is not high enough.

Authors Response
Thank you, we agree that interpretation of impedance spectra requires dedicated experimental investigation. In this paper we present the tools for such investigation, and now we present convincing experimental data. These measurements do now "demonstrate" the model suggested in this paper, which requires a more extensive ongoing experimentation, with variation of the conditions of the solar cells in the usual manner. However, the data show a strong connection of inverted hysteresis at low voltage, with tilted current, and the observation of the double inductor, which makes a strong motivation to use the suggested model as a tool for interpretation.

Modification of the main text and SI:

Under illumination (main text)

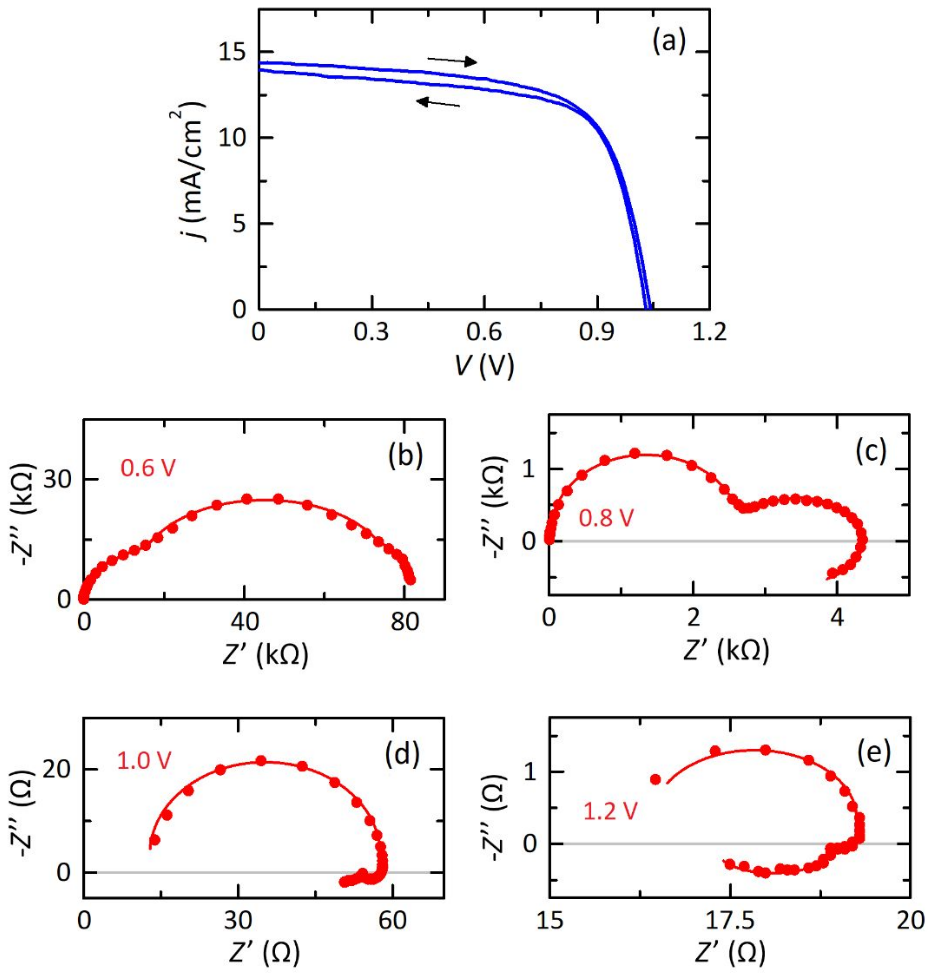

Fig. 1. Experimental responses of an inverted perovskite solar cells with a device layer architecture consisting of FTO/NiO/MeO-2PACz/perovskite ($Cs_{0.05}FA_{0.8}MA_{0.15}PbI_{2.75}Br_{0.25}$)/PCBM/BCP/Au/Ag. (a) Current-voltage curve obtained under a scan rate of 100 mV/s. Impedance spectra at (b) 0.6 V, (c) 0.8 V, (d) 1.0 V, and (e) 1.2 V under a frequency range from 1 MHz to 100 mHz.

In dark (SI)

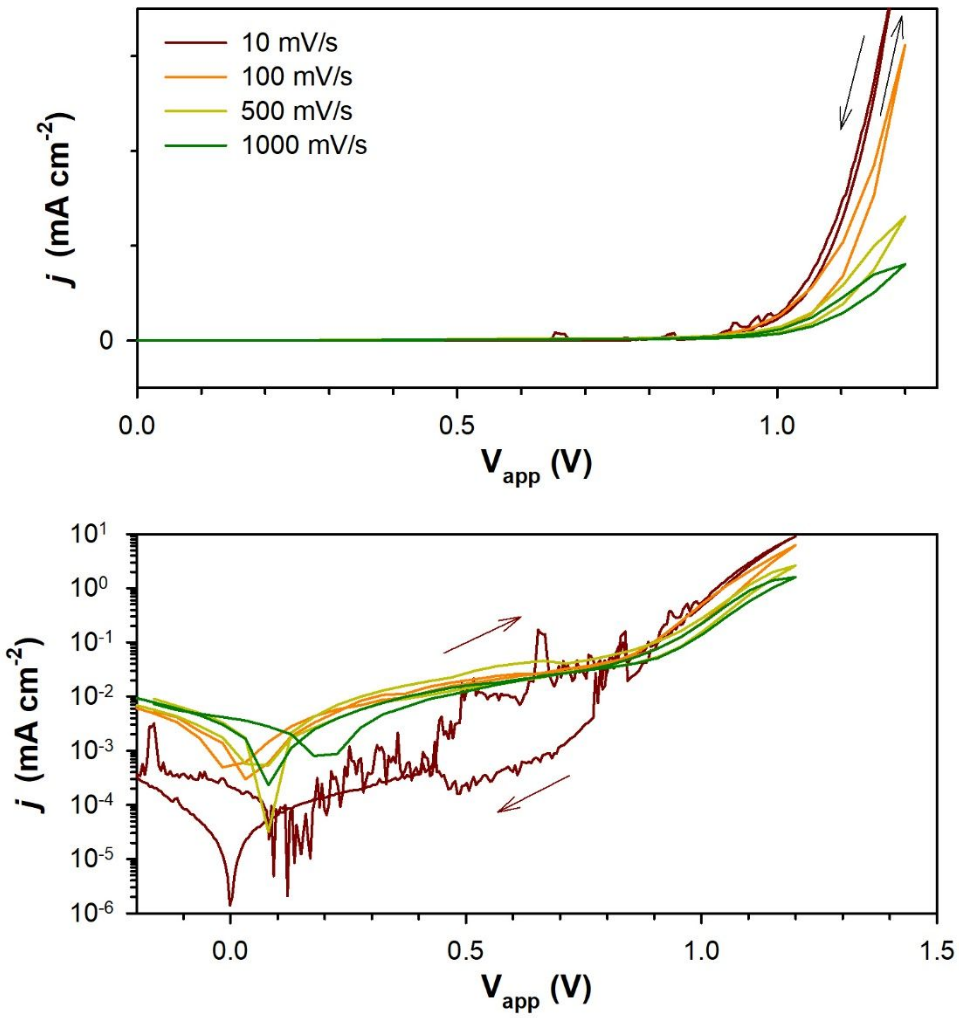

Fig. SI1. *J-V* response of a perovskite solar cell FTO/SnO_2/Perovskite/Spiro-OMeTAD/Au with the SnO_2 layer prepared by atomic layer deposition (ALD) measured under dark conditions and as a function of the scan rate. Linear (top) and semi-log (bottom) scale graphs are represented for clarity.

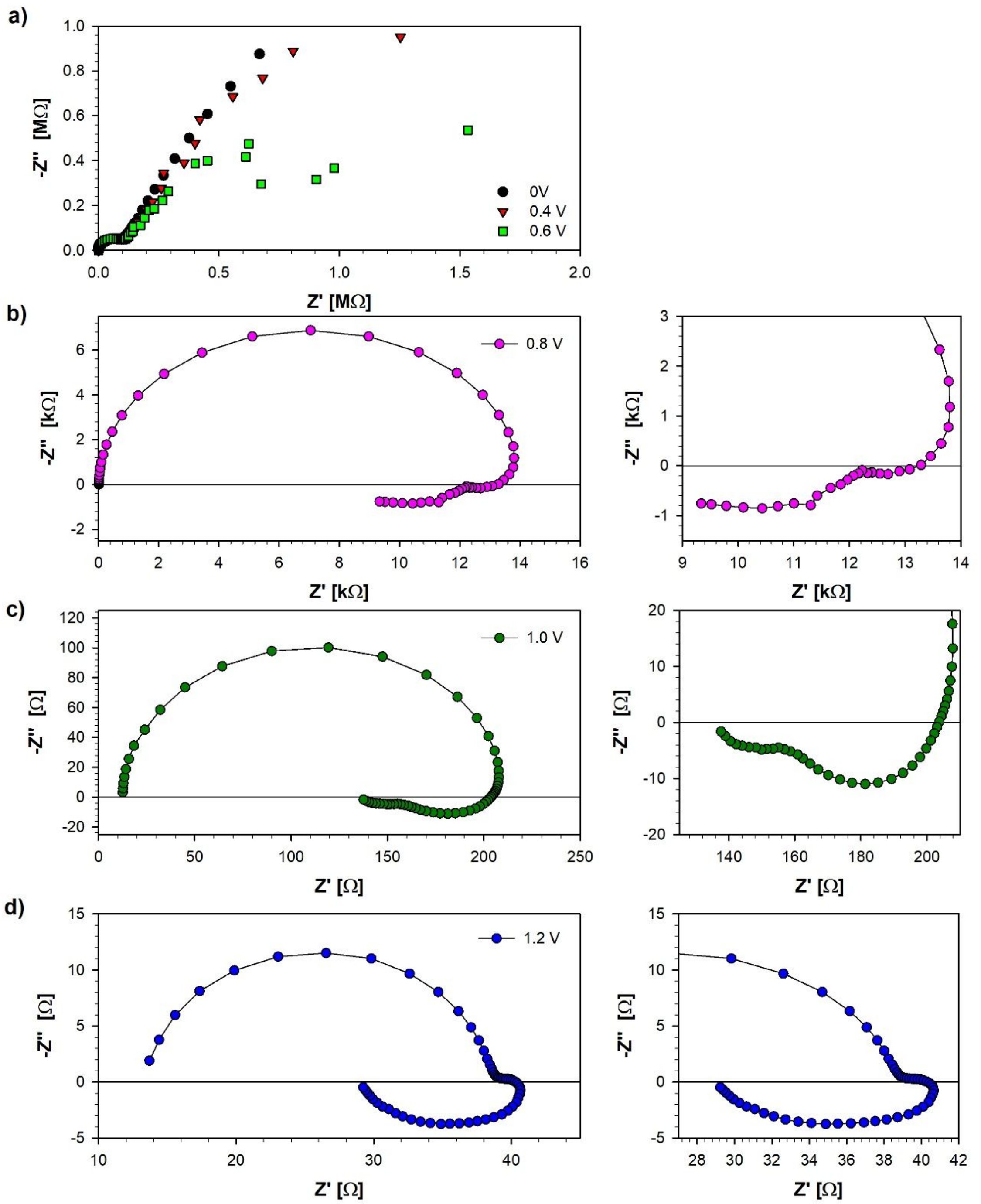

Fig. SI2 Complex impedance plot of measurements of a perovskite solar cell FTO/SnO_2/Perovskite/Spiro-OMeTAD/Au with the SnO_2 layer prepared by atomic layer deposition (ALD) measured in the dark under different DC voltages with. a) V_{DC} = 0 V, 0.4 V and 0.6 V, b) V_{DC} = 0.8 V, c) V_{DC} = 1.0 V and d) V_{DC} = 1.2 V. The insets on the right focus on the inductive features.

Comment 3. Technical suggestions
I suggest the authors confirm their findings with targeted experiments. Alternatively, they could review the existing literature to see how ionic effects in perovskite solar cells have been mitigated through additives or passivation strategies, and how this has manifested in distinct impedance responses.

Authors Response
Thank you, we have revised the literature and show the previous observations. As said above we give significant evidence of the experimental behaviour of hysteresis in relation to impedance measurements.

Additionally, some highly pertinent studies relevant to this context are missing from the references. This reviewer believes their inclusion is essential.

Authors Response
Thank you, we already have 80 references and more in the SI, but if we missed some important contribution we will be happy to include it.

Overall, this referee considers the proposed model interesting and promising, but related experimental evidence supporting the findings needs to be provided prior to publication.

Authors Response
Thank you, we hope the version of the paper is found acceptable.

Reviewer: 3

Recommendation: This paper may be publishable, but major revision is needed; I would like to be invited to review any future revision.

Comments:
The electro-ionic response of the perovskite solar cell is analyzed by proposing a unified model of recombination, polarization, electric field screening and charge collection effects. The unified model allows to calculate the impedance response to explain the low-frequency double inductor feature, linked separately to ion driven recombination and electric field screening effects. Interestingly, by comparing with impedance spectroscopy experimental data, this compact model can provide insights into the physical/degradation mechanisms hindering device performance. Due to the importance of the work, I highly recommend the publication of this work in JPCL after major and minor modifications.

Comment 1. The definition of V0 is quite interesting. The manuscript refers to it both as “a constant related to the built-in voltage” and as “the effective built-in voltage under

stationary operation." Could the authors please clarify what is meant by "stationary condition" in this context? Specifically, when is V0 defined—under equilibrium, in the dark, and at zero applied bias?

Authors Response

Thank you, we have provided a more clear statement. have expanded the explanation in the main text.

Modification of the main text

Here, V_0 is an effective built-in voltage under stationary operation. It corresponds to a specific steady state of bias voltage, illumination, etc. This V_0 may further depend nonlinearly on ionic components, as described by Nemnes et al [101] in a model imported from ferroelectric systems that show large hysteresis effects.[102]

Comment 2. Interpretation of Eq. (3): "Here, the internal voltage vb is the instantaneous bulk voltage that will equilibrate as vb→V in the long time, according to the equation." Based on this formulation, it appears that the voltage drop across the transport layers (TLs) is considered negligible. Is this assumption correct?

If so, I would suggest making this assumption explicit in the main text. In particular, under conditions where the transport layers are lightly doped and the ionic concentration in the perovskite is high, the voltage drop across the TLs can be non-negligible.

Authors response

Thank you, this is an important aspect of impedance modelling, we have provided an extended discussion in S4. Interpretation of voltages and model assumptions.

Modification of the main text

The seminal work of Garcia-Belmonte[46] showed that the low frequency interfacial capacitance includes components from both double layer and ionic transport charging. The transport layers may produce interference with the measured time constants.[47,48] It has also been remarked that the influence of ions in charge collection also affects surface recombination.[8] The buildup of ions can strengthen the internal drift field within the bulk, but it may also create unfavorable reversed electric field regions at the interfaces, where carriers become trapped and subsequently recombine via surface states.[49]

Comment 3. In the Table 1, I was surprised by the value of the geometrical capacitance Cg = 10-2 F. Could the authors please provide more detail on how this value was determined?

Authors response

Thank you. It was a mistake of units. It has been corrected.

Comment 4. In the conclusions, there is an interesting claim: “mobile ions in perovskite solar cells can lead to two primary effects affecting the stationary performance: an increase of recombination (lowering photovoltage) …”. I agree that, mobile ions screen the total electric field—comprising both internal and externally applied components—and can indeed lead to increased recombination and reduced photovoltage. However, this is not always the case. There are conditions, as when the TLs are lowly doped, in which ions can increase the quasi-fermi level splitting increasing the open circuit voltage. (for example: https://doi.org/10.1002/solr.202101087)

Authors response
This is very interesting, and we commented it.

Modification of the main text
By detailed interfacial models it is possible to establish the influence of ions on surface recombination, which can be beneficial for performance in some circumstances.[103,104]

Comment 5. In Figure 1. Please, check the format of the variables; current density and voltage which should be in italic.

Authors response
Thank you. It has been corrected

Comment 6. Figure 2.(b), there is also (a) inside the figure.

Authors response
Thank you. It has been corrected

Comment 7. In the abstract you claim that : “We show that the ordering of characteristic capacitive and inductive relaxation times provides a convenient criterion to classify impedance spectra and hysteresis effects.” Where is in the text the classification between IS and hysteresis effect?

Authors response
Thank you for this important comment. We have developed a new fundamental classification of capacitive and inductive hysteresis, based on the form of the current. This is presented in the Appendix. Furthermore we give specific examples of the classification of time constants in S3. Voltage cycling and hysteresis

Comment 8. "In Figure 2c we show that removing one time, τ → 0, the corresponding spectral feature disappears while the DC resistance is the same, since the resistors occur equally, although they turn into a fast response, when the respective τ can be neglected." What do you mean with "they turn into a fast response"?

Authors response

This explanation was deficient. It has been changed.

Modification of the main text

When the voltage is changed, parameters are modified and spectra can change drastically. If $L_b = 0$ and only a resistor remains so that τ→0. For the capacitor branch, usually interpreted as ionic conductance and polarization, $\tau_s = g_C^{-1}C_s$, so that τ→0 is obtained by large conductance or small capacitance.

Comment 9. In equation T11, Is the current Js the actual current Jd in T7?

Authors response

Thank you, this remark is correct.

Comment 10. At the end of the manuscript, there is an interesting analysis of the impact of the order of the different characteristic times. τg will be always smaller than τs. However, how can you distinguish the order between τd and τb? Both mechanisms, recombination and charge extraction, are slow. Or even, if you have only one inductive mechanism, how can you distinguish if it is coming from τd or τb?

Authors response

This is a very good question. It cannot be answered by modelling only. In order to identify the origin of experimental features, it is necessary to make measurement under different conditions of geometry, contacts, etc. For example, this is how the two standard impedance arcs of perovskite solar cells where interpreted long time ago.[44] This is how the correlation of surface inductor and capacitor was discovered as shown in the part B of the following figure.[20]

**A**

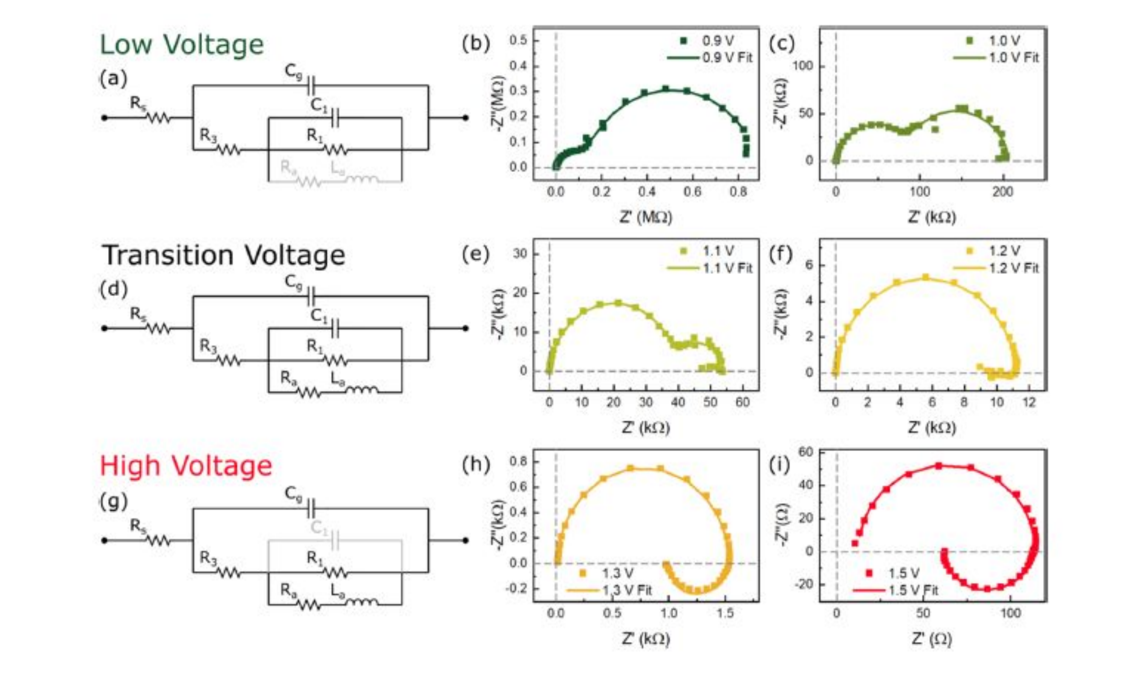

B

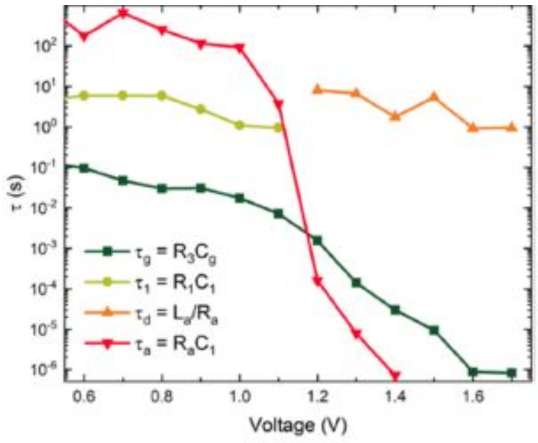

In this paper we provide reasonable tools for the two inductor phenomena, and the tilted jV curves, and some preliminary evidence, but detailed experimentation in the future is necessary.

(1) Ebadi, F.; Taghavinia, N.; Mohammadpour, R.; Hagfeldt, A.; Tress, W. Origin of apparent light-enhanced and negative capacitance in perovskite solar cells, *Nat. Commun.* **2019**, *10*, 1574.

(2) Khan, M. T.; Huang, P.; Almohammedi, A.; Kazim, S.; Ahmad, S. Mechanistic origin and unlocking of negative capacitance in perovskites solar cells, *iScience* **2021**, *24*, 102024.

(3) Alvarez, A. O.; Arcas, R.; Aranda, C. A.; Bethencourt, L.; Mas-Marzá, E.; Saliba, M.; Fabregat-Santiago, F. Negative Capacitance and Inverted Hysteresis: Matching Features in Perovskite Solar Cells, *J. Phys. Chem. Lett.* **2020**, *11*, 8417–8423.

(4) Bisquert, J. Hysteresis, Impedance, and Transients Effects in Halide Perovskite Solar Cells and Memory Devices Analysis by Neuron-Style Models, *Adv. Energy Mater.* **2024**, *n/a*, 2400442.

(5) Balaguera, E. H.; Bisquert, J. Evolution of Performance Parameters of Perovskite Solar Cells with Current–Voltage Scan Frequency, *Energy & Fuels* **2025**, *39*, 3638–3648.

(6) H. Balaguera, E.; Bisquert, J. Mapping of Internal Ionic/Electronic Transient Dynamics in Current–Voltage Operation of Perovskite Solar Cells, *Small* **2024**, *21*, 2409534.

(7) Balaguera, E. H.; Bisquert, J. Accelerating the Assessment of Hysteresis in Perovskite Solar Cells, *ACS Energy Lett.* **2024**, *9*, 478–486.

(8) Nicolae Filipoiu, A. T. P., Dragos-Victor Anghel, Roxana Patru, Rachel Elizabeth Brophy, Movaffaq Kateb, Cristina Besleaga, Andrei Gabriel Tomulescu, Ioana Pintilie, Andrei Manolescu, and George Alexandru Nemnes. Capacitive and inductive effects in perovskite solar cells: The different roles of ionic current and ionic charge accumulation, *Phys. Rev. Appl.* **2022**, *18*, 064087.

(9) Aranda, C. A.; Alvarez, A. O.; Chivrony, V. S.; Das, C.; Rai, M.; Saliba, M. Overcoming ionic migration in perovskite solar cells through alkali metals, *Joule* **2024**, *8*, 241–254.

(10) Pandey, S. V.; Prochowicz, D.; Mahapatra, A.; Pandiaraj, S.; Alodhayb, A.; Akin, S.; Yadav, P. The circuitry landscape of perovskite solar cells: An in-depth analysis, *Journal of Energy Chemistry* **2024**, *94*, 393–413.

(11) Wu, J.; Yang, C.; Luo, Z.; Wang, X.; Zheng, F.; Zhao, Z.; Hu, Z. Unified model for describing the evolution of negative capacitance in perovskite solar cells, *Phys. Rev. Appl.* **2024**, *22*, 024041.

(12) Hodgkin, A. L.; Huxley, A. F. A quantitative description of membrane current and its application to conduction and excitation in nerve, *J Physiol* **1952**, *117*,

500–544.

(13) Izhikevich, E. M. *Dynamical Systems in Neuroscience*; MIT Press, 2007.

(14) Izhikevich, E. M. Which model to use for cortical spiking neurons?, *IEEE Transactions on Neural Networks* **2004**, *15*, 1063–1070.

(15) Bisquert, J. Hysteresis, Rectification and Relaxation Times of Nanofluidic Pores for Neuromorphic Circuit Applications, *Advanced Physics Research* **2024**, *3*, 2400029.

(16) Chua, L. O.; Sung Mo, K. Memristive devices and systems, *Proceedings of the IEEE* **1976**, *64*, 209–223.

(17) Pershin, Y. V.; Di Ventra, M. Memory effects in complex materials and nanoscale systems, *Adv. Phys.* **2011**, *60*, 145–227.

(18) Bisquert, J.; Guerrero, A. Chemical Inductor, *J. Am. Chem. Soc.* **2022**, *144*, 5996–6009.

(19) Ghahremanirad, E.; Bou, A.; Olyaee, S.; Bisquert, J. Inductive Loop in the Impedance Response of Perovskite Solar Cells Explained by Surface Polarization Model, *J. Phys. Chem. Lett.* **2017**, *8*, 1402–1406.

(20) Gonzales, C.; Guerrero, A.; Bisquert, J. Transition from capacitive to inductive hysteresis: A neuron-style model to correlate I-V curves to impedances of metal halide perovskites, *J. Phys. Chem. C* **2022**, *126*, 13560–13578.

(21) Bisquert, J. Inductive and capacitive hysteresis of current-voltage curves. A unified structural dynamics in solar energy devices, memristors, ionic transistors and bioelectronics., *PRX Energy* **2023**, *3*, 011001.

(22) Chen, B.; Yang, M.; Zheng, X.; Wu, C.; Li, W.; Yan, Y.; Bisquert, J.; Garcia-Belmonte, G.; Zhu, K.; Priya, S. Impact of Capacitive Effect and Ion Migration on the Hysteretic Behavior of Perovskite Solar Cells, *J. Phys. Chem. Lett.* **2015**, *6*, 4693–4700.

(23) Kim, H.-S.; Jang, I.-H.; Ahn, N.; Choi, M.; Guerrero, A.; Bisquert, J.; Park, N.-G. Control of I-V Hysteresis in CH3NH3PbI3 Perovskite Solar Cell, *J. Phys. Chem. Lett.* **2015**, *6*, 4633–4639.

(24) Alam, M. A.; Si, M.; Ye, P. D. A critical review of recent progress on negative capacitance field-effect transistors, *Appl. Phys. Lett.* **2019**, *114*, 090401.

(25) Hernández-Balaguera, E.; Bisquert, J. Negative Transient Spikes in Halide Perovskites, *ACS Energy Lett.* **2022**, 2602–2610.

(26) Alvarez, A. O.; de Boer, J. J.; Sonneveld, L.; Bleiji, Y.; Alarcón-Lladó, E.; Ehrler, B. Hysteresis in Perovskite Devices: Understanding the Abrupt Resistive Switching Mechanism, *ACS Energy Lett.* **2025**, *10*, 3983–3992.

(27) Rong, Y. G.; Hu, Y.; Ravishankar, S.; Liu, H. W.; Hou, X. M.; Sheng, Y.

S.; Mei, A. Y.; Wang, Q. F.; Li, D. Y.; Xu, M.; Bisquert, J.; Han, H. W. Tunable hysteresis effect for perovskite solar cells, *Energy Environ. Sci.* **2017**, *10*, 2383–2391.

(28) Ravishankar, S.; Almora, O.; Echeverría-Arrondo, C.; Ghahremanirad, E.; Aranda, C.; Guerrero, A.; Fabregat-Santiago, F.; Zaban, A.; Garcia-Belmonte, G.; Bisquert, J. Surface Polarization Model for the Dynamic Hysteresis of Perovskite Solar Cells, *J. Phys. Chem. Lett.* **2017**, 915–921.

(29) Moia, D.; Gelmetti, I.; Calado, P.; Fisher, W.; Stringer, M.; Game, O.; Hu, Y.; Docampo, P.; Lidzey, D.; Palomares, E.; Nelson, J.; Barnes, P. R. F. Ionic-to-electronic current amplification in hybrid perovskite solar cells: ionically gated transistor-interface circuit model explains hysteresis and impedance of mixed conducting devices, *Energy Environ. Sci.* **2019**, *12*, 1296–1308.

(30) Torres, J.; Zarazua, I.; Esparza, D.; Rivas, J. M.; Saliba, M.; Mora-Seró, I.; Turren-Cruz, S.-H.; Abate, A. Degradation Analysis of Triple-Cation Perovskite Solar Cells by Electrochemical Impedance Spectroscopy, *ACS Appl. Ener. Mater.* **2022**, *5*, 12545–12552.

(31) Belisle, R. A.; Nguyen, W. H.; Bowring, A. R.; Calado, P.; Li, X.; Irvine, S. J. C.; McGehee, M. D.; Barnes, P. R. F.; O'Regan, B. C. Interpretation of inverted photocurrent transients in organic lead halide perovskite solar cells: proof of the field screening by mobile ions and determination of the space charge layer widths, *Energy Environ. Sci.* **2017**, *10*, 192–204.

(32) Tress, W.; Marinova, N.; Moehl, T.; Zakeeruddin, S. M.; Nazeeruddin, M. K.; Gratzel, M. Understanding the rate-dependent J-V hysteresis, slow time component, and aging in CH3NH3PbI3 perovskite solar cells: the role of a compensated electric field, *Energy Environ. Sci.* **2015**, *8*, 995–1004.

(33) Fabregat-Santiago, F.; Kulbak, M.; Zohar, A.; Vallés-Pelarda, M.; Hodes, G.; Cahen, D.; Mora-Seró, I. Deleterious Effect of Negative Capacitance on the Performance of Halide Perovskite Solar Cells, *ACS Energy Lett.* **2017**, *2*, 2007–2013.

(34) Guerrero, A.; Garcia-Belmonte, G.; Mora-Sero, I.; Bisquert, J.; Kang, Y. S.; Jacobsson, T. J.; Correa-Baena, J.-P.; Hagfeldt, A. Properties of Contact and Bulk Impedances in Hybrid Lead Halide Perovskite Solar Cells Including Inductive Loop Elements, *J. Phys. Chem. C* **2016**, *120*, 8023–8032.

(35) García-Rodríguez, R.; Riquelme, A. J.; Cowley, M.; Valadez-Villalobos, K.; Oskam, G.; Bennett, L. J.; Wolf, M. J.; Contreras-Bernal, L.; Cameron, P. J.; Walker, A. B.; Anta, J. A. Inverted Hysteresis in n–i–p and p–i–n Perovskite Solar Cells, *Energy Technology* **2022**, *10*, 2200507.

(36) Azpiroz, J. M.; Mosconi, E.; Bisquert, J.; De Angelis, F. Defect migration in methylammonium lead iodide and its role in perovskite solar cell operation, *Energy*

Environ. Sci. **2015**, *8*, 2118–2127.

(37) Wu, F.; Bahrami, B.; Chen, K.; Mabrouk, S.; Pathak, R.; Tong, Y.; Li, X.; Zhang, T.; Jian, R.; Qiao, Q. Bias-Dependent Normal and Inverted J–V Hysteresis in Perovskite Solar Cells, *ACS Appl. Mat. Int.* **2018**, *10*, 25604–25613.

(38) Kumar, A. Numerical modelling of ion-migration caused hysteresis in perovskite solar cells, *Optical and Quantum Electronics* **2021**, *53*, 166.

(39) Richardson, G.; O'Kane, S. E. J.; Niemann, R. G.; Peltola, T. A.; Foster, J. M.; Cameron, P. J.; Walker, A. B. Can slow-moving ions explain hysteresis in the current-voltage curves of perovskite solar cells?, *Energy Environ. Sci.* **2016**, *9*, 1476–1485.

(40) Boix, P. P.; Lee, Y. H.; Fabregat-Santiago, F.; Im, S. H.; Mora-Sero, I.; Bisquert, J.; Seok, S. I. From Flat to Nanostructured Photovoltaics: Balance between Thickness of the Absorber and Charge Screening in Sensitized Solar Cells, *ACS Nano* **2012**, *6*, 873–880.

(41) Kim, C. H.; Yaghmazadeh, O.; Bonnassieux, Y.; Horowitz, G. Modeling the low-voltage regime of organic diodes: Origin of the ideality factor, *J. Appl. Phys.* **2011**, *110*, 093722.

(42) Zhao, Y.; Liang, C.; Zhang, H.; Li, D.; Tian, D.; Li, G.; Jing, X.; Zhang, W.; Xiao, W.; Liu, Q.; Zhang, F.; He, Z. Anomalously large interface charge in polarity-switchable photovoltaic devices: an indication of mobile ions in organic–inorganic halide perovskites, *Energy Environ. Sci.* **2015**, *8*, 1256–1260.

(43) Gottesman, R.; Lopez-Varo, P.; Gouda, L.; Jimenez-Tejada, J. A.; Hu, J.; Tirosh, S.; Zaban, A.; Bisquert, J. Dynamic phenomena at perovskite/electron-selective contact interface as interpreted from photovoltage decays, *Chem* **2016**, *1*, 776–789.

(44) Zarazua, I.; Han, G.; Boix, P. P.; Mhaisalkar, S.; Fabregat-Santiago, F.; Mora-Seró, I.; Bisquert, J.; Garcia-Belmonte, G. Surface Recombination and Collection Efficiency in Perovskite Solar Cells from Impedance Analysis, *J. Phys. Chem. Lett.* **2016**, *7*, 5105–5113.

(45) Bou, A.; Pockett, A.; Raptis, D.; Watson, T.; Carnie, M. J.; Bisquert, J. Beyond Impedance Spectroscopy of Perovskite Solar Cells: Insights from the Spectral Correlation of the Electrooptical Frequency Techniques, *J. Phys. Chem. Lett.* **2020**, *11*, 8654–8659.

(46) Almora, O.; Guerrero, A.; Garcia-Belmonte, G. Ionic charging by local imbalance at interfaces in hybrid lead halide perovskites, *Appl. Phys. Lett.* **2016**, *108*, 043903.

(47) Ravishankar, S.; Kruppa, L.; Jenatsch, S.; Yan, G.; Wang, Y. Discerning rise time constants to quantify charge carrier extraction in perovskite solar cells, *Energy Environ. Sci.* **2024**, *17*, 1229–1243.

(48) Ravishankar, S.; Liu, Z.; Wang, Y.; Kirchartz, T.; Rau, U. How Charge Carrier Exchange between Absorber and Contact Influences Time Constants in the Frequency Domain Response of Perovskite Solar Cells, *PRX Energy* **2023**, *2*, 033006.

(49) Shen, H.; Jacobs, D. A.; Wu, Y.; Duong, T.; Peng, J.; Wen, X.; Fu, X.; Karuturi, S. K.; White, T. P.; Weber, K.; Catchpole, K. R. Inverted Hysteresis in CH3NH3PbI3 Solar Cells: Role of Stoichiometry and Band Alignment, *J. Phys. Chem. Lett.* **2017**, *8*, 2672–2680.

(50) Neukom, M. T.; Schiller, A.; Züfle, S.; Knapp, E.; Ávila, J.; Pérez-del-Rey, D.; Dreessen, C.; Zanoni, K. P. S.; Sessolo, M.; Bolink, H. J.; Ruhstaller, B. Consistent Device Simulation Model Describing Perovskite Solar Cells in Steady-State, Transient, and Frequency Domain, *ACS Appl. Mat. Int.* **2019**, *11*, 23320–23328.

(51) Clarke, W.; Richardson, G.; Cameron, P. Understanding the Full Zoo of Perovskite Solar Cell Impedance Spectra with the Standard Drift-Diffusion Model, *Adv. Energy Mater.* **2024**, *14*, 2400955.

(52) Clarke, W.; Cameron, P.; Richardson, G. Predicting Long-Term Stability from Short-Term Measurement: Insights from Modeling Degradation in Perovskite Solar Cells during Voltage Scans and Impedance Spectroscopy, *J. Phys. Chem. Lett.* **2024**, *15*, 11730–11736.

(53) Clarke, W.; Cowley, M. V.; Wolf, M. J.; Cameron, P.; Walker, A.; Richardson, G. Inverted hysteresis as a diagnostic tool for perovskite solar cells: Insights from the drift-diffusion model, *J. Appl. Phys.* **2023**, *133*, 095001.

(54) Riquelme, A. J.; Valadez-Villalobos, K.; Boix, P. P.; Oskam, G.; Mora-Seró, I.; Anta, J. A. Understanding equivalent circuits in perovskite solar cells. Insights from drift-diffusion simulation, *Phys. Chem. Chem. Phys.* **2022**, *24*, 15657–15671.

(55) Martani, S.; Zhou, Y.; Poli, I.; Aktas, E.; Meggiolaro, D.; Jiménez-López, J.; Wong, E. L.; Gregori, L.; Prato, M.; Di Girolamo, D.; Abate, A.; De Angelis, F.; Petrozza, A. Defect Engineering to Achieve Photostable Wide Bandgap Metal Halide Perovskites, *ACS Energy Lett.* **2023**, *8*, 2801–2808.

(56) Wang, Z. S.; An, Y.; Ren, X.; Zhang, H.; Huang, Z.; Yip, H.-L.; Huang, Z.; Choy, W. C. H. Device deficiency and degradation diagnosis model of Perovskite solar cells through hysteresis analysis, *Nat. Commun.* **2024**, *15*, 9647.

(57) Domanski, K.; Roose, B.; Matsui, T.; Saliba, M.; Turren-Cruz, S.-H.; Correa-Baena, J.-P.; Carmona, C. R.; Richardson, G.; Foster, J. M.; De Angelis, F.; Ball, J. M.; Petrozza, A.; Mine, N.; Nazeeruddin, M. K.; Tress, W.; Grätzel, M.; Steiner, U.; Hagfeldt, A.; Abate, A. Migration of cations induces reversible performance losses over day/night cycling in perovskite solar cells, *Energy Environ. Sci.* **2017**, *10*, 604–613.

(58) Guerrero, A.; Bisquert, J.; Garcia-Belmonte, G. Impedance spectroscopy

of metal halide perovskite solar cells from the perspective of equivalent circuits, *Chemical Reviews* **2021**, *121*, 14430–14484.

(59) Lopez-Varo, P.; Jiménez-Tejada, J. A.; García-Rosell, M.; Anta, J. A.; Ravishankar, S.; Bou, A.; Bisquert, J. Effects of Ion Distributions on Charge Collection in Perovskite Solar Cells, *ACS Energy Lett.* **2017**, *2*, 1450–1453.

(60) Sivadas, D.; Singareddy, A.; Vinod, C. G.; Nair, P. R. Ionic Charge Imbalance in Perovskite Solar Cells, *J. Phys. Chem. C* **2023**, *127*, 22766–22774.

(61) Diethelm, M.; Lukas, T.; Smith, J.; Dasgupta, A.; Caprioglio, P.; Futscher, M.; Hany, R.; Snaith, H. J. Probing ionic conductivity and electric field screening in perovskite solar cells: a novel exploration through ion drift currents, *Energy Environ. Sci.* **2025**, *18*, 1385–1397.

(62) Almora, O.; Aranda, C.; Garcia-Belmonte, G. Do Capacitance Measurements Reveal Light-Induced Bulk Dielectric Changes in Photovoltaic Perovskites?, *J. Phys. Chem. C* **2018**, *122*, 13450–13454.

(63) Almora, O.; Aranda, C.; Mas-Marzá, E.; Garcia-Belmonte, G. On Mott-Schottky analysis interpretation of capacitance measurements in organometal perovskite solar cells, *Appl. Phys. Lett.* **2016**, *109*, 173903.

(64) Ravishankar, S.; Bisquert, J.; Kirchartz, T. Interpretation of Mott–Schottky plots of photoanodes for water splitting, *Chemical Science* **2022**, *13*, 4828–4837.

(65) García-Batlle, M.; Mayén Guillén, J.; Chapran, M.; Baussens, O.; Zaccaro, J.; Verilhac, J.-M.; Gros-Daillon, E.; Guerrero, A.; Almora, O.; Garcia-Belmonte, G. Coupling between Ion Drift and Kinetics of Electronic Current Transients in MAPbBr3 Single Crystals, *ACS Energy Lett.* **2022**, *7*, 946–951.

(66) Almora, O.; Matt, G. J.; These, A.; Kanak, A.; Levchuk, I.; Shrestha, S.; Osvet, A.; Brabec, C. J.; Garcia-Belmonte, G. Surface versus Bulk Currents and Ionic Space-Charge Effects in CsPbBr3 Single Crystals, *J. Phys. Chem. Lett.* **2022**, 3824–3830.

(67) Almora, O.; Lopez-Varo, P.; Cho, K. T.; Aghazada, S.; Meng, W.; Hou, Y.; Echeverría-Arrondo, C.; Zimmermann, I.; Matt, G. J.; Jiménez-Tejada, J. A.; Brabec, C. J.; Nazeeruddin, M. K.; Garcia-Belmonte, G. Ionic dipolar switching hinders charge collection in perovskite solar cells with normal and inverted hysteresis, *Sol. En. Mater. Sol. Cell* **2019**, *195*, 291–298.

(68) Correa-Baena, J.-P.; Turren-Cruz, S.-H.; Tress, W.; Hagfeldt, A.; Aranda, C.; Shooshtari, L.; Bisquert, J.; Guerrero, A. Changes from Bulk to Surface Recombination Mechanisms between Pristine and Cycled Perovskite Solar Cells, *ACS Energy Lett.* **2017**, 681–688.

(69) Yoo, S.-M.; Yoon, S. J.; Anta, J. A.; Lee, H. J.; Boix, P. P.; Mora-Seró, I. An Equivalent Circuit for Perovskite Solar Cell Bridging Sensitized to Thin Film

Architectures, *Joule* **2019**, *3*, 2535–2549.

(70) Nandal, V.; Nair, P. R. Predictive Modeling of Ion Migration Induced Degradation in Perovskite Solar Cells, *ACS Nano* **2017**, *11*, 11505–11512.

(71) Schiller, A.; Jenatsch, S.; Blülle, B.; Torre Cachafeiro, M. A.; Ebadi, F.; Kabir, N.; Othman, M.; Wolff, C. M.; Hessler-Wyser, A.; Ballif, C.; Tress, W.; Ruhstaller, B. Assessing the Influence of Illumination on Ion Conductivity in Perovskite Solar Cells, *J. Phys. Chem. Lett.* **2024**, *15*, 11252–11258.

(72) Zhan, H.; Ahmad, V.; Mayon, A.; Dansoa Tabi, G.; Bui, A. D.; Li, Z.; Walter, D.; Nguyen, H.; Weber, K.; White, T.; Catchpole, K. Physics-based extraction of material parameters from perovskite experiments via Bayesian optimization, *Energy Environ. Sci.* **2024**, *17*, 4735–4745.

(73) Bennett, L. J.; Riquelme, A. J.; Anta, J. A.; Courtier, N. E.; Richardson, G. Avoiding Ionic Interference in Computing the Ideality Factor for Perovskite Solar Cells and an Analytical Theory of Their Impedance-Spectroscopy Response, *Phys. Rev. Appl.* **2023**, *19*, 014061.

(74) Riquelme, A.; Bennett, L. J.; Courtier, N. E.; Wolf, M. J.; Contreras-Bernal, L.; Walker, A. B.; Richardson, G.; Anta, J. A. Identification of recombination losses and charge collection efficiency in a perovskite solar cell by comparing impedance response to a drift-diffusion model, *Nanoscale* **2020**, *12*, 17385–17398.

(75) Almora, O.; López-Varo, P.; Escalante, R.; Mohanraj, J.; Marsal, L. F.; Olthof, S.; Anta, J. A. Instability analysis of perovskite solar cells via short-circuit impedance spectroscopy: A case study on NiOx passivation, *J. Appl. Phys.* **2024**, *136*, 094502.

(76) Aranda, C.; Bisquert, J.; Guerrero, A. Impedance spectroscopy of perovskite/contact interface: Beneficial chemical reactivity effect, *J. Chem. Phys.* **2019**, *151*, 124201.

(77) Zohar, A.; Kedem, N.; Levine, I.; Zohar, D.; Vilan, A.; Ehre, D.; Hodes, G.; Cahen, D. Impedance Spectroscopic Indication for Solid State Electrochemical Reaction in (CH3NH3)PbI3 Films, *J. Phys. Chem. Lett.* **2016**, *7*, 191–197.

(78) Guerrero, A.; You, J.; Aranda, C.; Kang, Y. S.; Garcia-Belmonte, G.; Zhou, H.; Bisquert, J.; Yang, Y. Interfacial degradation of planar lead halide perovskite solar cells, *ACS Nano* **2016**, *10*, 218–224.

(79) Domanski, K.; Correa-Baena, J.-P.; Mine, N.; Nazeeruddin, M. K.; Abate, A.; Saliba, M.; Tress, W.; Hagfeldt, A.; Grätzel, M. Not All That Glitters Is Gold: Metal-Migration-Induced Degradation in Perovskite Solar Cells, *ACS Nano* **2016**, *10*, 6306–6314.

(80) Bou, A.; Pockett, A.; Cruanyes, H.; Raptis, D.; Watson, T.; Carnie, M. J.;

Bisquert, J. Limited information of impedance spectroscopy about electronic diffusion transport: The case of perovskite solar cells, *APL Materials* **2022**, *10*, 051104.

(81) Ravishankar, S.; Aranda, C.; Sanchez, S.; Bisquert, J.; Saliba, M.; Garcia-Belmonte, G. Perovskite Solar Cell Modeling Using Light and Voltage Modulated Techniques, *J. Phys. Chem. C* **2019**, *123*, 6444–6449.

(82) Lopez-Richard, V.; Pradhan, S.; Wengenroth Silva, R. S.; Lipan, O.; Castelano, L. K.; Höfling, S.; Hartmann, F. Beyond equivalent circuit representations in nonlinear systems with inherent memory, *J. Appl. Phys.* **2024**, *136*, 165103.

(83) Gillespie, S. C.; Alvarez, A. O.; Thiesbrummel, J.; Gevaerts, V. S.; Geerligs, L. J.; Ehrler, B.; Coletti, G.; Garnett, E. C. Intensity-Modulated Photoluminescence Spectroscopy for Revealing Ionic Processes in Halide Perovskites, *ACS Energy Lett.* **2025**, *10*, 3122–3131.

(84) Hernandez-Balaguera, E.; Bisquert, J. Time Transients with Inductive Loop Traces in Metal Halide Perovskites, *Adv. Func. Mater.* **2023**, *34*, 2308678.

(85) Thiesbrummel, J.; Shah, S.; Gutierrez-Partida, E.; Zu, F.; Peña-Camargo, F.; Zeiske, S.; Diekmann, J.; Ye, F.; Peters, K. P.; Brinkmann, K. O.; Caprioglio, P.; Dasgupta, A.; Seo, S.; Adeleye, F. A.; Warby, J.; Jeangros, Q.; Lang, F.; Zhang, S.; Albrecht, S.; Riedl, T.; Armin, A.; Neher, D.; Koch, N.; Wu, Y.; Le Corre, V. M.; Snaith, H.; Stolterfoht, M. Ion-induced field screening as a dominant factor in perovskite solar cell operational stability, *Nat. Energy* **2024**, *9*, 664–676.

(86) Le Corre, V. M.; Diekmann, J.; Peña-Camargo, F.; Thiesbrummel, J.; Tokmoldin, N.; Gutierrez-Partida, E.; Peters, K. P.; Perdigón-Toro, L.; Futscher, M. H.; Lang, F.; Warby, J.; Snaith, H. J.; Neher, D.; Stolterfoht, M. Quantification of Efficiency Losses Due to Mobile Ions in Perovskite Solar Cells via Fast Hysteresis Measurements, *Solar RRL* **2022**, *6*, 2100772.

(87) Schmidt, M. C.; Ehrler, B. How Many Mobile Ions Can Electrical Measurements Detect in Perovskite Solar Cells?, *ACS Energy Lett.* **2025**, *10*, 2457–2460.

(88) Schmidt, M. C.; Alvarez, A. O.; de Boer, J. J.; van de Ven, L. J. M.; Ehrler, B. Consistent Interpretation of Time- and Frequency-Domain Traces of Ion Migration in Perovskite Semiconductors, *ACS Energy Lett.* **2024**, 5850–5858.

(89) Meggiolaro, D.; Mosconi, E.; De Angelis, F. Modeling the Interaction of Molecular Iodine with MAPbI3: A Probe of Lead-Halide Perovskites Defect Chemistry, *ACS Energy Lett.* **2018**, *3*, 447–451.

(90) Meggiolaro, D.; De Angelis, F. First-Principles Modeling of Defects in Lead Halide Perovskites: Best Practices and Open Issues, *ACS Energy Lett.* **2018**, *3*, 2206–2222.

(91) Kim, G. Y.; Senocrate, A.; Wang, Y.-R.; Moia, D.; Maier, J. Photo-Effect

on Ion Transport in Mixed Cation and Halide Perovskites and Implications for Photo-Demixing, *Angew. Chem. Int. Ed.* **2021**, *60*, 820–826.

(92) Senocrate, A.; Moudrakovski, I.; Kim, G. Y.; Yang, T.-Y.; Gregori, G.; Grätzel, M.; Maier, J. The Nature of Ion Conduction in Methylammonium Lead Iodide: A Multimethod Approach, *Angew. Chem. Int. Ed.* **2017**, *56*, 7755–7759.

(93) Bisquert, J. Theory of the impedance of electron diffusion and recombination in a thin layer, *J. Phys. Chem. B* **2002**, *106*, 325–333.

(94) Garcia-Belmonte, G.; Bisquert, J.; Pereira, E. C.; Fabregat-Santiago, F. Switching behaviour in lightly doped polymeric porous film electrodes. Improving distributed impedance models for mixed conduction conditions., *J. Electroanal. Chem.* **2001**, *508*, 48–58.

(95) Bisquert, J. Influence of the boundaries in the impedance of porous film electrodes, *Phys. Chem. Chem. Phys.* **2000**, *2*, 4185–4192.

(96) Bisquert, J.; Garcia-Belmonte, G.; Fabregat-Santiago, F.; Bueno, P. R. Theoretical models for ac impedance of diffusion layers exhibiting low frequency dispersion, *J. Electroanal. Chem.* **1999**, *475*, 152.

(97) Wang, Q.; Ito, S.; Grätzel, M.; Fabregat-Santiago, F.; Mora-Seró, I.; Bisquert, J.; Bessho, T.; Imai, H. Characteristics of high efficiency dye-sensitized solar cells, *J. Phys. Chem. B* **2006**, *110*, 19406–19411.

(98) Bou, A.; Āboliņš, H.; Ashoka, A.; Cruanyes, H.; Guerrero, A.; Deschler, F.; Bisquert, J. Extracting in Situ Charge Carrier Diffusion Parameters in Perovskite Solar Cells with Light Modulated Techniques, *ACS Energy Lett.* **2021**, 2248–2255.

(99) Pockett, A.; Spence, M.; Thomas, S. K.; Raptis, D.; Watson, T.; Carnie, M. J. Beyond the First Quadrant: Origin of the High Frequency Intensity-Modulated Photocurrent/Photovoltage Spectroscopy Response of Perovskite Solar Cells, *Solar RRL* **2021**, *5*, 2100159.

(100) Laird, J. S.; Ravishankar, S.; Rietwyk, K. J.; Mao, W.; Bach, U.; Smith, T. A. Intensity Modulated Photocurrent Microspectrosopy for Next Generation Photovoltaics, *Small Methods* **2022**, *6*, 2200493.

(101) Nemnes, G. A.; Besleaga, C.; Tomulescu, A. G.; Pintilie, I.; Pintilie, L.; Torfason, K.; Manolescu, A. Dynamic electrical behavior of halide perovskite based solar cells, *Sol. En. Mater. Sol. Cell* **2017**, *159*, 197–203.

(102) Pintilie, L.; Boldyreva, K.; Alexe, M.; Hesse, D. Capacitance tuning in antiferroelectric–ferroelectric PbZrO3–Pb(Zr0.8Ti0.2)O3 epitaxial multilayers, *New Journal of Physics* **2008**, *10*, 013003.

(103) Yekani, R.; Wang, H.; Bessette, S.; Gauvin, R.; Demopoulos, G. Synergetic interfacial conductivity modulation dictating hysteresis evolution in perovskite

solar cells under operation, *Phys. Chem. Chem. Phys.* **2024**, *26*, 8366–8379.

(104) Mozaffari, N.; Walter, D.; White, T. P.; Bui, A. D.; Tabi, G. D.; Weber, K.; Catchpole, K. R. Unraveling the Role of Energy Band Alignment and Mobile Ions on Interfacial Recombination in Perovskite Solar Cells, *Solar RRL* **2022**, *6*, 2101087.

jz-2025-01916q.R2

Name: Peer Review Information for "Dynamic Screening and the Chemical Inductor of Perovskite Solar Cells: From J–V Transients to Impedance Spectroscopy"

Second Round of Reviewer Comments

Reviewer: 2

Comments to the Author
The authors have satisfactorily addressed the requested changes in this revised manuscript. I recommend it for publication.

Reviewer: 1

Comments to the Author
The authors have provided an extensive response and have modified the manuscript accordingly to the requirements of the reviewers. Therefore, I recommend the publication of this article after some minor revisions are made.
1. The new experimental data that the authors present show inverted hysteresis at low voltages but not inductive features at such voltages. That seems surprising since they have been linked before, and in the manuscript, but appear now at different voltages. Is the model able to reproduce the impedance spectra at different voltages accordingly?
2. Figure 2c aims to illustrate the behavior of the electric field in transient conditions, but it is difficult to understand what happens with ions and how they provoke this.
3. ref 35 shows not inverted hysteresis but normal, according to the hysteresis indexes and the curves (which are really misleading). How unique is the inverted hysteresis at low voltages shown in here?
4. Although I have enjoyed reading through the discussion, there are several writing mistakes (missing words or half written phrases). Please, adequately and thoroughly revise the writing of the manuscript, since it makes losing focus on the discussion. In fact, the response to the reviewers starts with a wrong title of the manuscript. Please, be thorough in the revision.

Reviewer: 3

Comments to the Author
The authors have responded thoroughly to all questions raised by the reviewers. I appreciate the effort of the authors for the significant improvements made to the manuscript.
However, there are still some important modifications that need to be addressed before final acceptance.
Major Comments:

1.Figure 2 (a–c): The current depiction is confusing, and I suggest some modifications: (1) There should be a quasi-Fermi level splitting when an external voltage is applied. (2) It is not clear the selectivity in the contacts. (3) In panel (b), it is unclear how an electron appears in the conduction band if the work function of the left metal aligns closely with the valence band of the perovskite. Is this due to illumination? The pink metal is the "ETL-n-type contact" and the grey metal is the "HTL-p-type contact".
2. "The internal voltage vb is the instantaneous bulk voltage that will equilibrate as vb -> V in the long time", however, if I understood correctly, you also consider that the surface voltage vs Eq.T5 also will equilibrate to V, won't it?. Shouldn't be: vs + vb = V ? What is the relation between vs, vb and the applied voltage V?

Minor Comments:
3. "When the device is biased out of equilibrium, a drift current occurs, Fig. 2b." and also another current will come from diffusion, right?. To have only drift current, you need to surpass the built-in potential.
4. Figure 1. Please, could you include the full protocol to measure the JV curve in the Figure 1? What was the initial voltage?

Author's Response to Peer Review Comments:

We sincerely thank the reviewers for their thoughtful evaluation of our manuscript. We are very grateful for the insightful comments and constructive suggestions, which have significantly improved the clarity, rigor, and overall quality of our work.

Below, we describe the main modifications of the manuscript, and provide a detailed, point-by-point response to each reviewer comment. Reviewer comments appear in standard font, our replies are shown in blue, and modifications to the manuscript are highlighted in yellow.

Reviewer: 2

The authors have satisfactorily addressed the requested changes in this revised manuscript. I recommend it for publication.

Thank you

Reviewer: 1

Recommendation: This paper is publishable subject to minor revisions noted. Further review is not needed.

Comments:

The authors have provided an extensive response and have modified the manuscript accordingly to the requirements of the reviewers. Therefore, I recommend the publication of this article after some minor revisions are made.

Comment 1. The new experimental data that the authors present show inverted hysteresis at low voltages but not inductive features at such voltages. That seems surprising since they have been linked before, and in the manuscript, but appear now at different voltages. Is the model able to reproduce the impedance spectra at different voltages accordingly?

Authors response

There is a practical problem here, that we have explained:

Modification of the paper

We remark that impedance of perovskite solar cells is rarely reported below 0.5 V, since the recombination vanishes and the recombination resistance is huge. In Fig. 1, the inductor is observed at 0.8 V, before the onset of large recombination. The same in Fig. SI2. In both cases the inductor feature in the impedance corresponds to the region of hysteresis of the photocurrent. Obtaining the inductor at lower voltages may require specific experimental methods adapted to the purpose.

Comment 2. Figure 2c aims to illustrate the behavior of the electric field in transient conditions, but it is difficult to understand what happens with ions and how they provoke this.

Authors response

Thank you, we acknowledge the complexity of the response, hence we have made a more complete model, explained in the Section 4 of the SI.

Modification of the paper

In the model, mobile ions in mixed ionic–electronic solar cells accumulate at the selective contacts and form Helmholtz double layers, which act as nanocapacitors storing part of the built-in potential. This electrostatic screening reduces the fraction of the voltage that drops across the semiconductor bulk, weakening the internal field and enhancing recombination. Under steady bias the voltage is shared between semiconductor and Helmholtz layers according to their capacitances: if ions are mobile, the Helmholtz voltage decreases proportionally with applied forward bias (relaxed case); if ions are immobile, the Helmholtz voltage remains fixed and the semiconductor takes the full change (frozen case). In the latter situation, recombination grows more rapidly and the net photocurrent is reduced. This is shown in Fig. SI4.

After a sudden voltage step, the difference between these two regimes becomes explicit. Immediately, the ionic configuration is frozen, so the semiconductor experiences the entire perturbation and the current drops below its steady-state value. With time, ions redistribute, the Helmholtz layers (HL) readjust their voltage, and the current recovers. This transient dip and recovery are not explained by conventional RC charging but correspond to the chemical inductor effect:[1] Ionic motion modulates recombination with a delay, producing an inductive-like signature in impedance spectroscopy and anomalous current transients. These results highlight the central role of Helmholtz screening and ion dynamics in interpreting jV and impedance measurements of perovskites and related semiconductors.

Comment 3. ref 35 shows not inverted hysteresis but normal, according to the hysteresis indexes and the curves (which are really misleading). How unique is the inverted hysteresis at low voltages shown in here?

Authors response

Thank you, unfortunately the jV curves are not reported, only the PCE hysteresis, so we cannot provide a concrete interpretation.

Comment 4. Although I have enjoyed reading through the discussion, there are several writing mistakes (missing words or half written phrases). Please, adequately and thoroughly revise the writing of the manuscript, since it makes losing focus on the discussion. In fact, the response to the reviewers starts with a wrong title of the manuscript. Please, be thorough in the revision.

Authors response

Thank you, the paper has been revised again by all authors.

Reviewer: 3

Comments:

The authors have responded thoroughly to all questions raised by the reviewers. I appreciate the effort of the authors for the significant improvements made to the manuscript.

However, there are still some important modifications that need to be addressed before final acceptance.

Major Comments:

Comment 1. Figure 2 (a–c): The current depiction is confusing, and I suggest some modifications: (1) There should be a quasi-Fermi level splitting when an external voltage is applied. (2) It is not clear the selectivity in the contacts. (3) In panel (b), it is unclear how an electron appears in the conduction band if the work function of the left metal aligns closely with the valence band of the perovskite. Is this due to illumination? The pink metal is the "ETL-n-type contact" and the grey metal is the "HTL-p-type contact".

Authors response

We are very grateful to the reviewer for rightfully pointing our these limitations and inconsistencies. The Fig. 2 has been remade, and a full drift-diffusion model has been solved in sec. 4 of the SI to produce a consistent picture.

Modification of the paper

This type of hysteresis of the photocurrent is often interpreted according to slow ionic interfacial polarization that affects the bulk electrical field and modifies the photocurrent altering the charge collection. This mechanism has been described many times.[2-8] It is well known that the drift field can be manipulated with slow polarization of ions. One example is the famous "preconditioning" treatment by applying a specific voltage or illumination protocol to the device before measurement, to influence ion distribution.[7]

This can lead to improved performance by enhancing built-in electric fields.[9] In general, the light soaking can modify the ion distribution and change significantly the internal field.[10,11] Here, we provide a summary explanation based on the elementary mechanism of Fig. 2, explained in more detail in Sec. 4 of the SI.

In the model, mobile ions in mixed ionic–electronic solar cells accumulate at the selective contacts and form Helmholtz double layers (HL), which act as nanocapacitors storing part of the built-in potential. The voltage is shared between semiconductor and Helmholtz layers according to their capacitances. This electrostatic screening reduces the fraction of the voltage that drops across the semiconductor bulk, weakening the internal field, which affects charge collection and recombination, provided that the diffusion length L_D is short. The surface ion distribution requires some time τ_k for the excess ions to adapt to the new voltage,[11] and we make a distinction of relaxed and "frozen" ionic states.[12,13] If ions are mobile, the Helmholtz voltage decreases proportionally with applied forward bias (relaxed case); if ions are immobile, the Helmholtz voltage remains fixed and the semiconductor takes the full change (frozen case). This is shown in Fig. SI4.

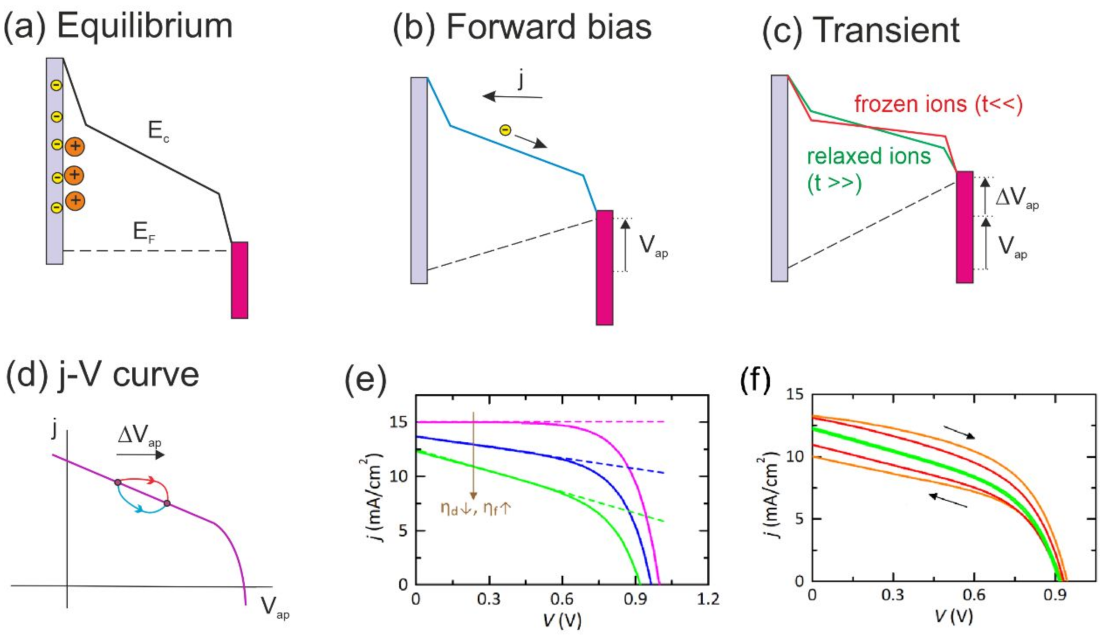

Fig. 2. (a-b) Basic model of a solar cell where carrier transport is driven by drift in the electrical field, with different biasing conditions. The electron selective contact is the pink layer at the right, and the hole selective contact is the grey layer at the left. The valence band is parallel to the conduction band level, and it is not shown. The dipole layer in the hole selective contact is formed by electrons (yellow) and cations (orange). c) Effect of a change of voltage. Initially (red) the dipole layers remain in the previous configuration, since the ion rearrangement is slow. When the ions relax at $t \gg \tau_k$, the dipole layers are

reduced (green), and the current increases. (d). The model generates an undershoot (blue) or an overshoot (red) of photocurrent, according to the dominant effect of charge collection or recombination, respectively. (f, g) Simulation of model curves. (e) Steady state current-voltage characteristics. The vertical arrow indicates the changes of collection efficiencies: $\eta_d = 1, 0.85, 0.7; \eta_f = 0, 0.1, 0.2$, and the dashed straight lines show j_{ph} (V, Φ). (f) Hysteresis in the green curve of (a) by increasing the sweep velocity, $f = 1$ Hz (orange), 0.5 Hz (red). Parameters $V_m = -0.2$ V, $V_p = 1.2$ V.

Comment 2. "The internal voltage vb is the instantaneous bulk voltage that will equilibrate as vb -> V in the long time", however, if I understood correctly, you also consider that the surface voltage vs Eq.T5 also will equilibrate to V, won't it?. Shouldn't be: vs + vb = V ? What is the relation between vs, vb and the applied voltage V?

Authors response

We have explained this point in Sec. 6 of the SI. Additionally, we show in Sec. 4 of the SI a model with the internal voltages separated.

Minor Comments:

Comment 3. "When the device is biased out of equilibrium, a drift current occurs, Fig. 2b." and also another current will come from diffusion, right?. To have only drift current, you need to surpass the built-in potential.

Authors response

Totally right. To solve this important question, we have provided drift-diffusion calculations insect. S4 of the SI, where the problem mentioned by the reviewer is eliminated.

4. Figure 1. Please, could you include the full protocol to measure the JV curve in the Figure 1? What was the initial voltage?

Modification of the paper

Fig. 1. Experimental responses of an inverted perovskite solar cell with a device layer architecture consisting of FTO/NiO/MeO-2PACz/perovskite ($Cs_{0.05}FA_{0.8}MA_{0.15}PbI_{2.75}Br_{0.25}$)/PCBM/BCP/Au/Ag. (a) Current-voltage curve obtained under a scan rate of 100 mV/s. The device was measured from -0.2 V to 1.2 V in forward and reverse directions under AM1.5 G spectrum in ambient conditions (22°C and 30% RH). Impedance spectra at (b) 0.6 V, (c) 0.8 V, (d) 1.0 V, and (e) 1.2 V under a frequency

range from 1 MHz to 100 mHz.

(1) Bisquert, J.; Guerrero, A. Chemical Inductor, *J. Am. Chem. Soc.* **2022**, *144*, 5996–6009.

(2) Azpiroz, J. M.; Mosconi, E.; Bisquert, J.; De Angelis, F. Defect migration in methylammonium lead iodide and its role in perovskite solar cell operation, *Energy Environ. Sci.* **2015**, *8*, 2118–2127.

(3) Wu, F.; Bahrami, B.; Chen, K.; Mabrouk, S.; Pathak, R.; Tong, Y.; Li, X.; Zhang, T.; Jian, R.; Qiao, Q. Bias-Dependent Normal and Inverted J–V Hysteresis in Perovskite Solar Cells, *ACS Appl. Mat. Int.* **2018**, *10*, 25604–25613.

(4) Kumar, A. Numerical modelling of ion-migration caused hysteresis in perovskite solar cells, *Optical and Quantum Electronics* **2021**, *53*, 166.

(5) Richardson, G.; O'Kane, S. E. J.; Niemann, R. G.; Peltola, T. A.; Foster, J. M.; Cameron, P. J.; Walker, A. B. Can slow-moving ions explain hysteresis in the current-voltage curves of perovskite solar cells?, *Energy Environ. Sci.* **2016**, *9*, 1476–1485.

(6) García-Rodríguez, R.; Riquelme, A. J.; Cowley, M.; Valadez-Villalobos, K.; Oskam, G.; Bennett, L. J.; Wolf, M. J.; Contreras-Bernal, L.; Cameron, P. J.; Walker, A. B.; Anta, J. A. Inverted Hysteresis in n–i–p and p–i–n Perovskite Solar Cells, *Energy Technology* **2022**, *10*, 2200507.

(7) Belisle, R. A.; Nguyen, W. H.; Bowring, A. R.; Calado, P.; Li, X.; Irvine, S. J. C.; McGehee, M. D.; Barnes, P. R. F.; O'Regan, B. C. Interpretation of inverted photocurrent transients in organic lead halide perovskite solar cells: proof of the field screening by mobile ions and determination of the space charge layer widths, *Energy Environ. Sci.* **2017**, *10*, 192–204.

(8) Boix, P. P.; Lee, Y. H.; Fabregat-Santiago, F.; Im, S. H.; Mora-Sero, I.; Bisquert, J.; Seok, S. I. From Flat to Nanostructured Photovoltaics: Balance between Thickness of the Absorber and Charge Screening in Sensitized Solar Cells, *ACS Nano* **2012**, *6*, 873–880.

(9) Zhang, Y.; Liu, M.; Eperon, G. E.; Leijtens, T. C.; McMeekin, D.; Saliba, M.; Zhang, W.; de Bastiani, M.; Petrozza, A.; Herz, L. M.; Johnston, M. B.; Lin, H.; Snaith, H. J. Charge selective contacts, mobile ions and anomalous hysteresis in organic–inorganic perovskite solar cells, *Materials Horizons* **2015**, *2*, 315–322.

(10) Deng, X.; Wen, X.; Zheng, J.; Young, T.; Lau, C. F. J.; Kim, J.; Green, M.; Huang, S.; Ho-Baillie, A. Dynamic study of the light soaking effect on perovskite solar

cells by in-situ photoluminescence microscopy, *Nano Energy* **2018**, *46*, 356–364.

(11) Gottesman, R.; Lopez-Varo, P.; Gouda, L.; Jimenez-Tejada, J. A.; Hu, J.; Tirosh, S.; Zaban, A.; Bisquert, J. Dynamic phenomena at perovskite/electron-selective contact interface as interpreted from photovoltage decays, *Chem* **2016**, *1*, 776–789.

(12) Thiesbrummel, J.; Shah, S.; Gutierrez-Partida, E.; Zu, F.; Peña-Camargo, F.; Zeiske, S.; Diekmann, J.; Ye, F.; Peters, K. P.; Brinkmann, K. O.; Caprioglio, P.; Dasgupta, A.; Seo, S.; Adeleye, F. A.; Warby, J.; Jeangros, Q.; Lang, F.; Zhang, S.; Albrecht, S.; Riedl, T.; Armin, A.; Neher, D.; Koch, N.; Wu, Y.; Le Corre, V. M.; Snaith, H.; Stolterfoht, M. Ion-induced field screening as a dominant factor in perovskite solar cell operational stability, *Nat. Energy* **2024**, *9*, 664–676.

(13) H. Balaguera, E.; Marinelli Pra, F. J.; Das, C.; Torresani, L.; Bisquert, J.; Saliba, M. 'Ion-freeze' efficiency in perovskite solar cells: Time scales for ion immobilization, *EES Solar* **2025**.

jz-2025-01916q.R3
Name: Peer Review Information for "Dynamic Screening and the Chemical Inductor of Perovskite Solar Cells: From J–V Transients to Impedance Spectroscopy"

Third Round of Reviewer Comments
Reviewer: 3

Dear Authors,

Thanks for the revision and answering all the questions.

Please, check once again my previous comment 1.

Comment 1. Figure 2 (a–c): The current depiction is confusing, and I suggest some modifications: (1) There should be a quasi-Fermi level splitting when an external voltage is applied. (2) It is not clear the selectivity in the contacts. (3) In panel (b), it is unclear how an electron appears in the conduction band if the work function of the left metal aligns closely with the valence band of the perovskite. Is this due to illumination?

Here, I have just made a schematic to illustrate my comment.

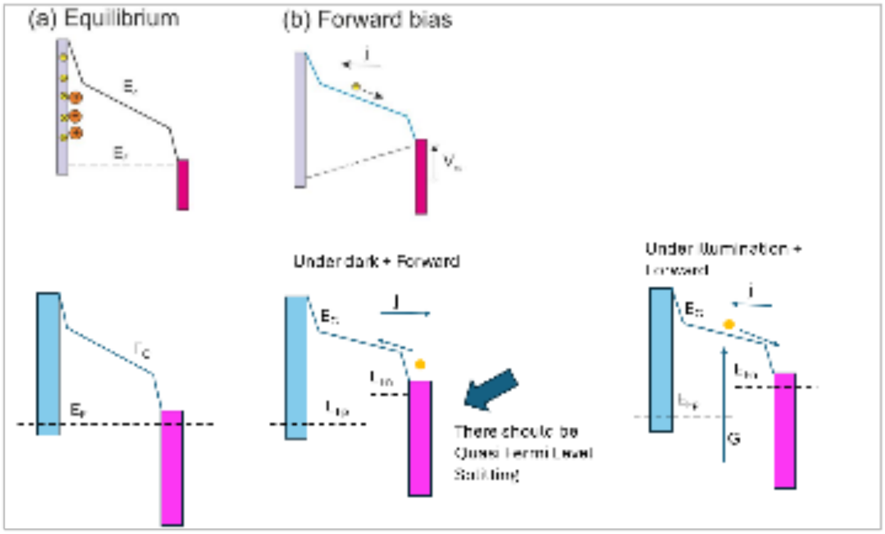

Comment 2. "The internal voltage vb is the instantaneous bulk voltage that will equilibrate as vb -> V in the long time", however, if I understood correctly, you also consider that the surface voltage vs Eq.T5 also will equilibrate to V, won't it?. Shouldn't be: vs + vb = V ? What is the relation between vs, vb and the applied voltage V?

Thanks for the answer to my comment 2.

In the text, you mentioned the following:

In our model we have made the definition that both voltage variables equilibrate to the same voltage: $v_s \to V$, $v_b \to V$. This does not mean that both internal voltages coincide with the whole external voltage V. There may be a constant relation, such that $v_s \to V - V_{s0}$, for

Therefore, if I understood correctly, mathematically, both values can reach the applied voltage V; but I understand that is an approximation. From my point of view, the total voltage in the device could be expressed as: vs + vb = V – Vbi.

Author's Response to Peer Review Comments:

We sincerely thank again the reviewer for their thoughtful evaluation of our manuscript.

Reviewer: 3

Comment 1. Figure 2 (a–c): The current depiction is confusing, and I suggest some modifications: (1) There should be a quasi-Fermi level splitting when an external voltage is applied. (2) It is not clear the selectivity in the contacts. (3) In panel (b), it is unclear how an electron appears in the conduction band if the work function of the left metal aligns closely with the valence band of the perovskite. Is this due to illumination?

Here, I have just made a schematic to illustrate my comment.

Authors response

Thank you, we understand the point. There are many aspects of the model that can be further discussed. However, we have focussed on the critical question here, which is the effect of the slope of the conduction band on the transport, as this is what we are going to compare. The Fermi levels, the valence band, etc has been suppressed. The fact that the model is realistic is shown in the following figures, made by drift-diffusion simulation, and shown in the SI.

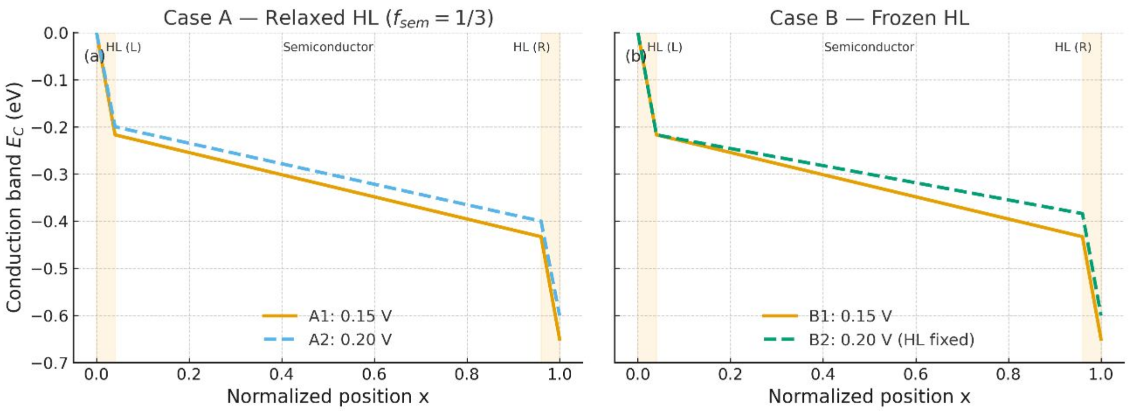

Fig. SI4. Conduction band profiles of a selective-contact solar cell including Helmholtz layers at the interfaces. The horizontal axis shows the normalized device thickness, with shaded regions indicating the left and right Helmholtz layers (HL) and the central region corresponding to the semiconductor bulk. The vertical axis is the conduction band energy E_C (eV), plotted between –1.0 and 0.0 eV. (a) Case A — Relaxed HL (capacitive partition with $f_{sem} = 1/3$): profiles are shown for applied voltages V_{app} = 0.15 V (solid line, A1) and 0.20 V (dashed line, A2). Both the HL and semiconductor drops are reduced proportionally when the applied voltage increases. (b) Case B — Frozen HL (HL potential fixed at its 0.15 V value): profiles are shown for V_{app} = 0.15 V (solid line, B1) and 0.20 V

(dashed line, B2). The HL potential remains constant while the semiconductor absorbs the entire additional applied voltage.

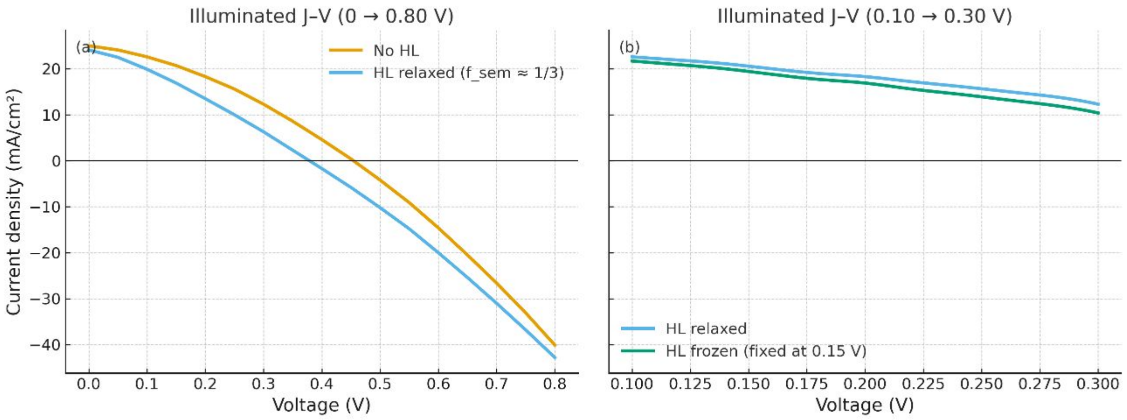

Fig. SI5**.** Illuminated current–voltage characteristics of a selective-contact solar cell under different assumptions for the Helmholtz layers (HL). All currents are given in mA cm^{-2}. (a) Full voltage range (0 ⟶ 0.80 V), comparing the case without Helmholtz layers (orange), in which the full electrostatic drop is inside the semiconductor, and the case with relaxed Helmholtz layers (blue), where the applied bias is shared between the HL and the semiconductor according to capacitive partition ($f_{sem} \approx 1/3$). (b) Zoomed voltage range (0.10 ⟶ 0.30 V), comparing relaxed HL (blue) with frozen HL (green). In the relaxed case, the HL potential adapts at each voltage, while in the frozen case the HL potential is held fixed to its value at 0.15 V, so the semiconductor absorbs the entire additional voltage increment. The curves are smoothed interpolations of the calculated data.

Comment 2. "The internal voltage vb is the instantaneous bulk voltage that will equilibrate as vb -> V in the long time", however, if I understood correctly, you also consider that the surface voltage vs Eq.T5 also will equilibrate to V, won't it?. Shouldn't be: vs + vb = V ? What is the relation between vs, vb and the applied voltage V?

In the text, you mentioned the following:

Therefore, if I understood correctly, mathematically, both values can reach the applied voltage V; but I understand that is an approximation. From my point of view, the total voltage in the device could be expressed as: vs + vb = V – Vbi.

Authors response

Thank you, this point is explained in the Supporting Information:

In our model we have made the definition that both voltage variables equilibrate to the same voltage: $v_s \to V$, $v_b \to V$. This does not mean that both internal voltages coincide with the whole external voltage V. There may be a constant relation, such that $v_s \to V - V_{s0}$, for instance. However, this will only introduce another constant parameter V_{s0} that we cannot measure, since it is absorbed by the prefactor of the capacitance.
